# The First Chalcone Derivatives of Valine‐Based Spiro‐Cyclotriphosphazenes: In Vitro Cytotoxic Properties, Molecular Docking and DNA Damage Mechanism Studies

**DOI:** 10.1002/jbt.70233

**Published:** 2025-03-24

**Authors:** Yunus Yücel, Ferhan Sultan Şeker, Büşra Aksoy Erden, Mücahit Özdemir, Çiğdem Tekin, Eray Çalışkan, Suat Tekin, Kenan Koran, Fatih Biryan

**Affiliations:** ^1^ Faculty of Science, Department of Chemistry Fırat University Elazig Turkey; ^2^ Department of Biotechnology, Faculty of Science Bartin University Bartin Turkey; ^3^ Department of Chemistry Marmara University Kadikoy Istanbul Turkey; ^4^ İnönü Üniversitesi, Tıp Fakültesi, Halk Sağlığı A.D. Malatya Turkey; ^5^ Department of Chemistry Bingol University Bingöl Turkey; ^6^ İnönü Üniversitesi, Tıp Fakültesi, Fizyoloji Malatya Turkey

**Keywords:** cyclotriphosphazene, cytotoxicity, genotoxicity, molecular docking, TUNEL analysis

## Abstract

Cancer treatment requires novel compounds with potent cytotoxic and genotoxic properties to effectively target cancer cells. In this study, new hybrid cyclotriphosphazene compounds were synthesized, characterized, and evaluated for their biological activity. Cytotoxicity against A2780 and Caco‐2 cancer cell lines was assessed via the MTT assay, while genotoxic effects at 60–70% cell viability were examined using the Comet assay. Apoptotic cells were identified through TUNEL analyses, and reactive oxygen species levels were measured. Results showed that these compounds significantly reduced cell viability through DNA damage mechanisms. At high doses (50–100 µM), BV, BVK1, BVK2, and BVK4 decreased A2780 cell viability by 30–65%, whereas VPA had a milder effect (15–25%). In Caco‐2 cells, viability was reduced by 10–35%. The compounds exhibited varying cytotoxicity across different cancer cell lines, reflecting cancer cell heterogeneity. Significant DNA damage, including changes in tail length, tail density, and tail moment, was observed in A2780 cells, confirming cell death via DNA damage. Molecular docking analyses further supported the potential of cyclotriphosphazene compounds (BV and BVK2) as targeted cancer inhibitors. Molecular docking revealed BVK2's high selectivity for Bcl‐2, mutant p53, and VEGFR2. BVK2 and BV demonstrate strong binding affinities with key cancer‐related targets, indicating their potential as multi‐targeted inhibitors that regulate apoptosis, cell cycle control, and angiogenesis, making them promising candidates for targeted cancer therapy.

## Graphical abstract

This study synthesizes and evaluates hybrid cyclotriphosphazene compounds for cancer treatment. The compounds exhibited significant cytotoxicity and genotoxicity against A2780 and Caco‐2 cell lines, inducing DNA damage and apoptosis, with molecular docking suggesting their potential as targeted inhibitors for cancer therapy.

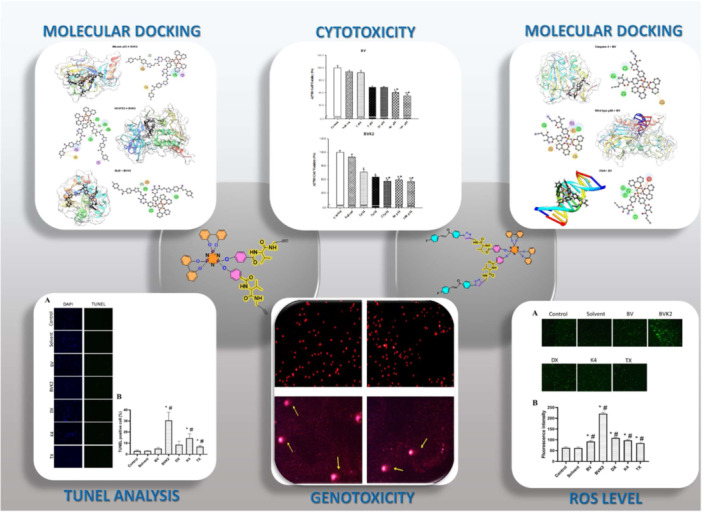

## Introduction

Cancer is one of the most common and deadly diseases worldwide, affecting millions of people every year, according to the World Health Organization [1, 2]. Although traditional methods such as chemotherapy, radiotherapy, and surgery are widely used in cancer treatment, the need for new treatment approaches continues due to the side effects of these treatments and their limited efficacy in some cancer types [3, 4, 5]. Developments in chemistry and biotechnology hold promise for the development of more targeted and effective cancer treatment methods. In this context, hybrid structures of organic and inorganic compounds attract attention thanks to the advantages they offer in their interaction with biological systems. Cyclotriphosphazenes are compounds formed by the combination of phosphorus and nitrogen atoms in a ring structure [6, 7] and the fact that the chemical structures of these compounds can be easily modified makes them attractive in drug development studies [8, 9, 10, 11, 12, 13]

Amino acids are the basic building blocks of cells; they are the molecules that form the structure of proteins and play critical roles in biological processes. The high bioavailability of amino acids and their compatibility with biological systems make them important components in drug delivery systems and therapeutic agents [14, 15, 16, 17, 18]. Chalcones are natural compounds belonging to the flavonoid family and are known for their broad spectrum of biological activities. Their antioxidant, anti‐inflammatory, antimicrobial, and anticancer properties highlight chalcones as potential therapeutic agents [19, 20, 21, 22]. Combining the chemical structures of amino acids and chalcones with cyclotriphosphazene rings can synergistically enhance the biological activities of these compounds and optimize their efficacy in targeted therapeutic approaches [23]. In particular, the combination of amino acid and chalcone groups on cyclotriphosphazenes has the potential to exert specific effects at the cellular level, which offers a great opportunity for developing new hybrid compounds [10, 12, 24].

**Scheme 1:**
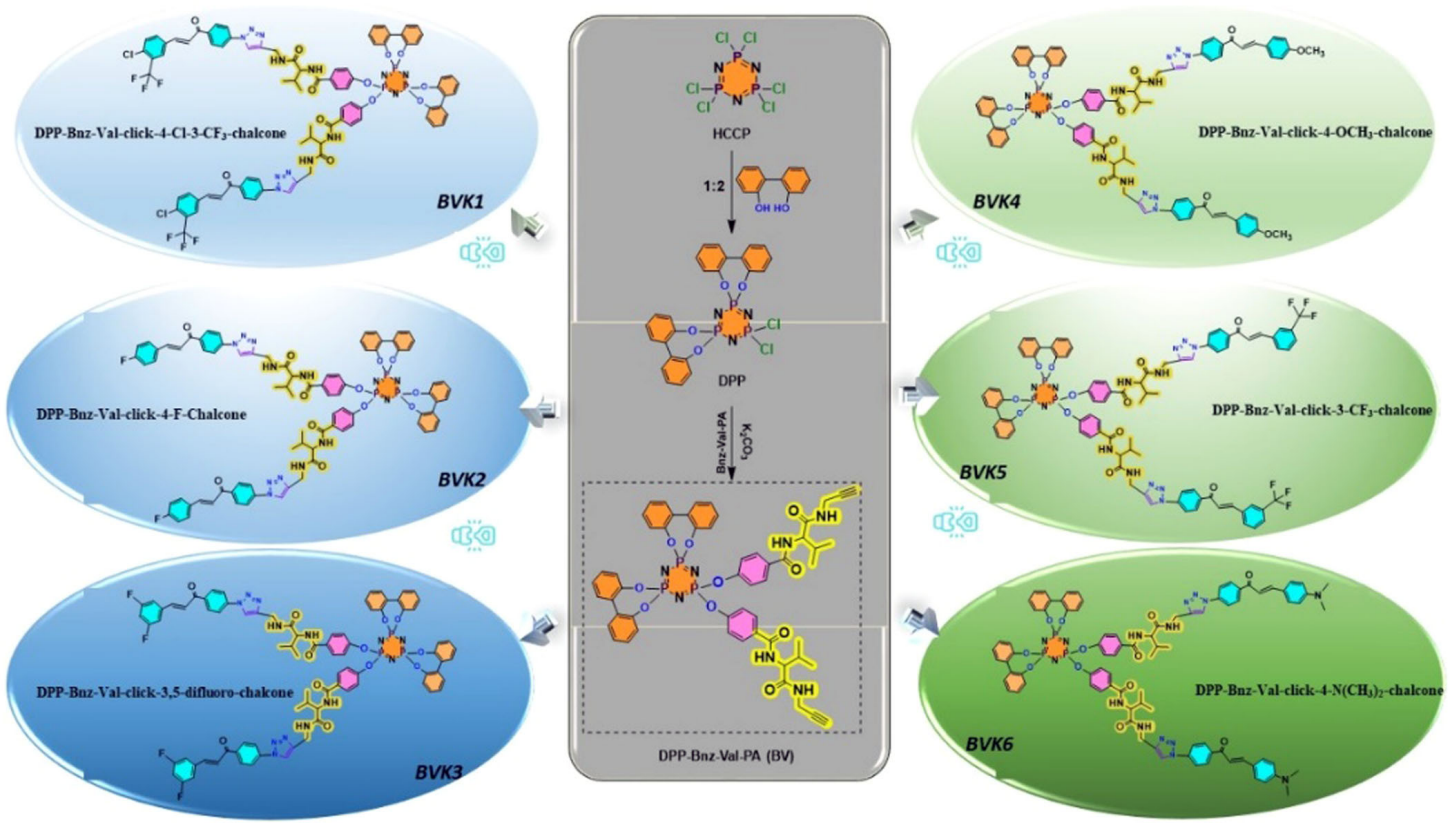
General illustration of synthetic route.

This study aims to synthesize and characterize novel hybrid cyclotriphosphazene derivatives and evaluate their cytotoxic, genotoxic, and apoptotic effects on cancer cells, along with molecular docking analyses to explore their potential as targeted anticancer agents. In vitro, cytotoxicity studies were carried out against A2780 and Caco2 cancer cell lines by the MTT assay method. In addition, the compounds’ genotoxicity effects at doses providing 60‐70% viability were evaluated by the Comet assay method. TUNEL analyses were performed to determine apoptotic cells, and reactive oxygen species (ROS) analyses were also performed, and the results were examined comparatively. Molecular docking analyses reveal that BVK2 exhibits high selectivity and strong binding affinities with key cancer‐related targets, including Bcl‐2 (−11.4 kcal/mol), mutant p53 (−9.2 kcal/mol), and VEGFR2 (−11.9 kcal/mol), suggesting its potential as a multi‐targeted inhibitor. BVK2 effectively interacts with Bcl‐2 through hydrogen bonds (Gly142, Asn140, Arg143) and π‐anion interactions (Asp137), contributing to apoptosis regulation. Its binding with mutant p53 involves hydrogen bonding (Thr150, Ser227) and π‐based interactions (Asp228, Val147, Cys220), indicating its role in cell cycle control. Additionally, BVK2's strong interactions with VEGFR2 via hydrogen bonds (Arg840, Gly839, Arg1030, Arg1064) and π‐sulfur interactions (Cys917) highlight its potential to inhibit angiogenesis and tumor growth. Similarly, the BV compound exhibits high binding affinity with Caspase‐3 (−10.1 kcal/mol), wild‐type p53 (−9.3 kcal/mol), and DNA (−8.4 kcal/mol), suggesting its role in apoptosis induction and tumor suppression. Comparative analysis with reference drugs, such as docetaxel and tamoxifen, demonstrates that BV and BVK2 exhibit superior binding affinities, supporting their promise as targeted cancer therapeutics with potentially higher efficacy and lower toxicity. These findings suggest that cyclotriphosphazene compounds can be evaluated as potential new drug candidates in cancer treatment (Scheme 1).

## 1 Materials and Methods

### 1.1 Chemistry

Hexachlorocyclotriphosphazene (trimer) was purchased from Alfa Aesar and the starting compounds were crystallized in n‐hexane before the synthesis of DPP. Triethylamine (Et_3_N), N,N’‐Dicyclohexylcarbodiimide (DCC), sodium carbonate (NaHCO_3_), chloroform, tetrahydrofuran, acetonitrile, anhydrous magnesium carbonate (MgSO_4_), potassium carbonate (K_2_CO_3_) and tetrahydrofuran (THF) were provided by Merck. K_2_CO_3_ was kept overnight in an oven before use. Sodium hydroxide (NaOH), n‐hexane, ethyl acetate (EtOAc), dimethyl sulphoxide (%99.9), ethyl alcohol was obtained from TEKKİM; glycine, valine, alanine, phenyl alanine and leucine methyl esters, Boc‐l‐tyrosine and 2‐Chloro‐4,6‐dimethoxy‐1,3,5‐triazine (CDMT) were obtained from Chem‐Impex INT'L INC; 4‐Methylmorpholine (NMM) was obtained from ROTH Chemicals. DMSO‐d_6_ and CDCl_3_ used as deuterium solvents for NMR studies were obtained from Merck, Euroisotop and Across.

### 1.2 General Procedure for Synthesis of N_3_‐Acetophenone (**N**_3_ **‐AF**)

For N_3_‐acetophenone compound [25, 26] 6 g of 4‐amino acetophenone was dissolved in 3 M HCl in a reaction flask and the temperature of the mixture was brought between 0°C and 5°C. 3.68 g NaNO_2_ aqueous solution was added slowly to this mixture and stirred for 30 min. 5.77 g NaN_3_ was dissolved in water and added dropwise to the mixture and the reaction was continued at room temperature for 3 h. After extraction with diethyl ether, the solvent of the organic part was removed to obtain N_3_‐AF compound [25]. FT‐IR (cm‐^1^, ATR) (Figure S1): **ν**_**C‐H(Aromatic)**_:3060, 3003; **ν**_**C‐H(Aliphatic)**_: 2964; **ν**_**N3**_: 2093**; ν**_**C═O**_: 1678 (C═O); **ν**_**C═C**_: 1594 (C═C). ^1^H NMR (400 MHz, CDCl_3_‐d, δ)(Figure S2): 7.98 (d, *J* = 8.7 Hz, 2H, H^3^), 7.10 (d, *J* = 8.6 Hz, 2H, H^2^), 2.60 (s, 3H, H^6^). ^13^C NMR (CDCl_3_‐d, δ) (Figure S3): 26.54 C^6^, 118.98 C^3^, 130.30 C^4^, 133.79 C^4^, 144.91 C^1^, 196.65 C^5^.

### 1.3 General Procedure for Synthesis of Azido Chalcones (**K4** and **K6**)

All Chalcone compounds were prepared according to the Claisen‐Schmidt condensation [27, 28] *4‐Cl‐3‐CF*_*3*_ *‐chalcone‐N*_*3*_ (***K1***): Yield %68. FT‐IR (cm‐^1^, ATR) (Figure S4): **ν**_**C‐H(Aromatic)**_: 3106, 3075, 3043; **ν ν**_**N3**_: 2115, 2096**; ν**_**C═O**_: 1656 (C═O); **ν**_**C═C**_: 1596, 1572 (C═C). ^1^H NMR (400 MHz, DMSO‐*d*_6_) (Figure S5): δ 8.41 (s, 1H), 8.26 (t, *J* = 8.9 Hz, 3H), 8.15 (d, *J* = 15.7 Hz, 1H), 7.87–7.78 (m, 2H), 7.31 (d, *J* = 8.7 Hz, 2H). ^13^C NMR (101 MHz, DMSO) (Figure S5): δ 116.3, 116.5, 119.8, 122.2, 127.6, 131.1, 131.8, 131.9, 134.6, 143.2, 144.8, 162.7, 165.1, 187.9.

*4‐F‐chalcone‐N*_*3*_ (***K2***): Yield 86%. FT‐IR (cm‐^1^, ATR) (Figure S6): **ν**_**C‐H(Aromatic)**_: 3109, 3042; **ν ν**_**N3**_: 2104**; ν**_**C═O**_: 1654 (C═O); **ν**_**C═C**_: 1595 (C═C aromatic). ^1^H NMR (400 MHz, DMSO‐*d*_6_) (Figure S7): δ 8.23 (d, *J* = 8.6 Hz, 2H), 7.99 (dd, *J* = 8.7, 5.7 Hz, 2H), 7.93 (d, *J* = 15.6 Hz, 1H), 7.76 (d, *J* = 15.6 Hz, 1H), 7.36–7.26 (m, 4H). ^13^C NMR (101 MHz, DMSO) (Figure S8): δ 116.5, 119.8, 122.2, 131.1, 131.9, 134.6, 143.2, 144.8, 162.7, 165.1, 189.0.

*3,5‐DiF‐chalcone‐N*_*3*_ (***K3***): Yield 86%. FT‐IR FT‐IR (cm‐^1^, ATR) (Figure 9): **ν**_**C‐H(Aromatic)**_: 3060, 3083; **ν**_**N3**_: 2108, 2085**; ν**_**C═O**_: 1665 (C═O); **ν**_**C═C**_: 1587, 1596 (C═C aromatic). ^1^H NMR (400 MHz, DMSO‐*d*_6_) (Figure S10): δ 8.26 (d, *J* = 8.6 Hz, 2H), 8.10 (d, *J* = 15.5 Hz, 1H), 7.82–7.67 (m, 3H), 7.42–7.28 (m, 3H). ^13^C NMR (101 MHz, DMSO) (Figure S11): δ 106.1, 112.4, 119.9, 124.9, 131.3, 134.3, 139.0, 141.8, 145.2, 161.8, 164.4, 187.8.

*4‐OCH*_*3*_ *‐chalcone‐N*_*3*_ *(**K4**):* Yield %75. FT‐IR (cm‐^1^, ATR) (Figure S12): **ν**_**C‐H(Aromatic)**_: 3106, 3075, 3043; **ν ν**_**N3**_: 2115, 2096**; ν**_**C═O**_: 1656 (C═O); **ν**_**C═C**_: 1596, 1572 (C═C). ^1^H NMR (400 MHz, DMSO‐d6, δ) (Figure S13): 8.41 (s, 1H, H^9^), 8.26 (t, *J* = 8.9 Hz, 3H, H^3^ ve H^7^), 8.15 (d, *J* = 15.7 Hz, 1H, H^6^), 7.87–7.78 (m, 2H, H^12^ ve H^13^), 7.31 (d, *J* = 8.7 Hz, 2H, H^2^). ^13^C NMR (DMSO‐d_6_, δ) (Figure S14): 116.30 C^6^, 116.52 C^9^, 119.84 C^2^, 122.19 C^12^, 127.64 C^14^, 131.13 C^3^, 131.81 C^13^, 131.86 C^1^°, 134.64 C^11^, 143.23 C^7^, 144.84 C^8^, 162.67 C^4^, 165.15 C^1^, 187.98 C^5^.

*3‐CF*_*3*_ *‐chalcone‐N*_*3*_ (***K5***): Yield %78. FT‐IR (cm‐^1^, ATR) (Figure S15): **ν**_**C‐H(Aromatic)**_: 3089, 3066, 3043; **ν ν**_**N3**_: 2117, 2096**; ν**_**C═O**_: 1654 (C═O); **ν**_**C═C**_: 1592, 1571 (C═C). ^1^H NMR (400 MHz, DMSO‐*d*_6_) (Figure S16): δ 8.41–8.34 (m, 1H), 8.28 (d, *J* = 8.7 Hz, 2H), 8.24–8.12 (m, 2H), 7.89–7.79 (m, 2H), 7.71 (t, *J* = 7.8 Hz, 1H), 7.32 (d, *J* = 8.6 Hz, 2H). ^13^C NMR (101 MHz, DMSO) (Figure S17): δ 119.9, 124.2, 125.6, 125.9, 127.2, 130.1, 130.4, 131.3, 133.4, 134.4, 136.3, 142.6, 145.1, 188.0.

*4‐N(CH*_*3*_ *)*_*2*_ *‐chalcone‐N*_*3*_ *(**K6**):* Yield %75. FT‐IR (cm‐^1^, ATR) (Figure S18): **ν**_**C‐H(Aromatic)**_: 3066, 3014; **ν ν**_**N3**_: 2108, 2087**; ν**_**C═O**_: 1653 (C═O); **ν**_**C═C**_: 1596, 1583 (C═C). ^1^H NMR (400 MHz, DMSO‐d6, δ) (Figure S19): 8.20 (d, *J* = 7.9 Hz, 2H, H^3^), 7.94–7.78 (m, 3H, H^9^ and H^7^), 7.72 (d, *J* = 15.3 Hz, 1H, H^6^), 7.27 (d, *J* = 7.9 Hz, 2H, H^2^), 7.02 (d, *J* = 7.9 Hz, 2H, H^10^), 3.82 (s, 3H, H^12^). ^13^C NMR (DMSO‐d6, δ) (Figure S20): 55.84 C^12^, 114.86 C^10^, 119.66 C^6^, 119.76 C^2^, 127.78 C^8^, 130.98 C^3^, 131.32 C^9^, 134.93 C^4^, 144.47 C^7^, 144.54 C^1^, 161.86 C^11^, 187.84 C^5^.

### 1.4 General Procedure for Synthesis of OH‐Bnz‐Val‐OCH_3_

4 g of 4‐hydroxy benzoic acid was dissolved in acetonitrile (MeCN) followed by the addition of 4.95 grams (1 eq) l‐valine methyl ester hydrochloride and 4.87 mL (1.20 eq) triethylamine (Et_3_N) and the reaction continued at this temperature for about 20 min after the temperature was cooled to 0°C. To the mixture was added 7.24 grams (1.20 eq) of *N,N’*‐Dicyclohexylcarbodiimide (DCC) dissolved in MeCN [29]. The reaction was followed with a thin layer (EtOAc:Hexane 3:5). After completion of the reaction, the mixture was vacuum filtered with silica gel to remove the by‐product dicyclohexylurea (DCU). The filtrate was completely evaporated and the remaining oily fraction was dissolved in ethyl acetate (EtOAc) and extracted with 10% citric acid, 5% sodium carbonate (NaCO_3_) and distilled water, respectively. The organic part was dried with MgSO_4_ and the solvent was evaporated. The residue was dissolved in chloroform and precipitated in n‐hexane. The precipitated solid was filtered and dried. 4‐Hydroxy benzoyl phenylalanine methyl ester compound (HO‐Bnz‐Val‐OMe) was obtained as a white solid yield 75%. FT‐IR (cm‐^1^, ATR) (Figure S21):: **ν**_**NH‐OH**_: 3380, 3320, 3145 **ν**_**C‐H(Aromatic)**_: 3062, 3092; **ν**_**C═O**_: 1627, 1739 (C═O); **ν**_**C═C**_: 1543, 1584 (C═C). ^1^H NMR (400 MHz, DMSO‐d6, δ) (Figure S22): 0.99 (6H, s, H^24^), 2.13–2.22 (1H, m, H^23^), 3.65 (3H, s, H^26^), 4.24–4.28 (1H, H^22^), 6.81–6.83 (2H, d, J = 8.8 Hz, H^17^), 7.77–7.79 (2H, d, J = 8.4 Hz, H^18^), 8.30–8.31 (1H, d, H^21^ (‐NH)), 10.02 (1H, s, H^27^ (‐Ph‐OH)). ^13^C NMR (Figure S23): 160.70 C^16^, 058.69 C^22^, 115.15 C^17^, 029.98 C^23^, 130.02 C^18^, 019.80 C^24^, 125.27 C^19^, 173.81 C^25^, 166.93 C^20^, 054.73 C^26^.

### 1.5 General Procedure for Synthesis of OH‐Bnz‐Val‐OH

To break the ester group of HO‐Bnz‐Val‐OCH_3_ compound, it was reacted with 8% NaOH solution in methanol in cold medium [30, 31, 32]. The reaction was stopped by monitoring with TLC. The compound HO‐Bnz‐Val‐OH was obtained by breaking the ester group. Yield is 82%. FT‐IR (cm‐^1^, ATR) (Figure S24): **ν**_**NH‐OH**_: 3303, 3402; **ν**_**C‐H(Aromatic)**_: 3030; **ν**_**C═O**_: 1605, 1619, 1701 (C═O); **ν**_**C═C**_: 1517, 1543, 1583 (C═C). ^1^H NMR (400 MHz, DMSO‐d6, δ) (Figure S25): 0.94–0.98 (6H, s, H^24^), 2.14–2.19 (1H, m, H^23^), 4.23–4.26 (1H, H^22^), 6.80–6.82 (2H, d, J = 8.4 Hz, H^17^), 7.77–7.79 (2H, d, J = 8.4 Hz, H^18^), 8.32–8.34 (1H, d, H^21^ (‐NH)), 10.01 (1H, s, H^27^ (‐Ph‐OH)), 12.58 (1H, s, H^26^ (‐CO‐OH)). ^13^C NMR (Figure S26): 160.70 C^16^, 058.71 C^22^, 115.15 C^17^, 029.66 C^23^, 130.04 C^18^, 019.32 C^24^, 125.24 C^19^, 173.85 C^25^, 166.94 C^20^.

### 1.6 General Procedure for Synthesis of OH‐Bnz‐Val‐PA (**VPA**)

3.51 mmol HO‐Bnz‐Val‐OH, 4.21 mmol 2‐Chloro‐4,6‐dimethoxy‐1,3,5‐triazine (CDMT), and 3.51 mmol propargyl amine compounds were dissolved in acetonitrile and *N‐*Methylmorpholine (NMM) (8.76 mmol, 2.5 eq.) was slowly added dropwise to the resulting suspension and stirred at room temperature. After the TLC, half of the solvent was evaporated and precipitated in water. Yield is 72%. FT‐IR (cm‐^1^, ATR) (Figure S27): **ν**_**NH‐OH**_: 3276, 3322; **ν**_**C‐H(Aromatic)**_: 3071; **ν**_**C═O**_: 1622, 1663 (C═O); **ν**_**C═C**_: 1502, 1543 (C═C). ^1^H NMR (400 MHz, DMSO‐d6, δ) (Figure S28): 0.90–0.92 (6H, s, H^24^), 2.07–2.13 (1H, m, H^23^), 3.11–3.12 (1H, s, H^29^), 3.81–3.94 (2H, m, H^27^), 4.21–4.26 (1H, d, H^22^), 6.79–6.81 (2H, d, *J* = 8 Hz, H^17^), 7.76–7.79 (2H, d, J = 8.4 Hz, H^18^), 8.01–8.03 (1H, t, H^26^ (‐NH)), 8.49–8.50 (1H, d, H^21^ (‐NH)), 9.99 (1H, s, H^30^ (‐Ph‐OH)). ^13^C NMR (Figure S29): 160.97 C^16^, 030.67 C^23^, 115.20 C^17^, 019.47 C^24^, 129.98 C^18^, 171.77 C^25^, 125.02 C^19^, 028.23 C^27^, 166.51 C^20^, 081.54 C^28^, 059.22 C^22^, 073.37 C^29^.

### 1.7 General Procedure for Synthesis of **DPP**

After preparation of the argon airless reaction system, 144.80 mmol K_2_CO_3_ was added to a three‐mouth flask containing 150 mL acetone. 57.49 mmol of 2,2’‐biphenol was added and the reaction medium was cooled to 0°C with ice [6, 33]. The mixture was stirred for 5–10 min and the reaction was initiated by adding 29.38 mmol hexachlorocyclotrifosphazene (HCCP). The reaction was continued at room temperature and argon atmosphere for 2 h and stopped. The solvent of the reaction, acetone, was removed by a rotary evaporator under vacuum. The solid remaining in the flask was extracted with dichloromethane (5 × 50 mL). Dichloromethane was removed by rotary evaporator and white solid was obtained. After crystallization of the DPP compound in acetone, 14.32 g compound was obtained (yield 85%) [34]. The FT‐IR, ^31^P‐NMR, ^1^H‐NMR and ^13^C‐NMR spectra were given Figure S30–33.

### 1.8 General Procedure for Synthesis of DPP‐Bnz‐Val‐PA (**BV**)

80 mL acetone was added to a 250 mL reaction flask under argon atmosphere and stirred. In the presence of argon gas, 0.3 g (0.52 mmol) DPP compound was added and stirred until dissolution. 0.25 g (1.83 mmol) K_2_CO_3_ was added and stirred for 10 min. Finally, 0.41 g (1.57 mmol) OH‐Bnz‐Val‐PA was added to the reaction mixture and stirred at room temperature for 5 min. The reaction mixture was heated at the boiling point of acetone and stirred overnight. The reaction was monitored with a thin layer and stopped after about 24 h. After the reaction was stopped, the salts formed in the mixture were separated by filtration. The solvent of the remaining filtrate was evaporated in acetone rotary evaporator. The solid remaining in the flask was dissolved in chloroform (insoluble filtered) and precipitated in n‐hexane. The precipitated white colored solid was obtained as pure DPP‐Bnz‐Val‐PA. White solid, 0.45 g, 82%. FT‐IR (cm‐^1^, ATR) (Figure S34): **ν**_**N‐H**_: 3295 **ν**_**C‐H(Aromatic)**_: 3066; **ν**_**C═O**_: 1636 (C═O); **ν**_**C═C**_: 1518 (C═C); **ν**_**P=N**_: 1163, 1232. ^1^H NMR (400 MHz, DMSO‐d6, δ) (Figure S36): δ: 0.93 (12H, s, H^24^), 2.07–2.15 (2H, m, H^23^), 3.10 (2H, s, H^29^), 3.83–3.94 (4H, m, H^27^), 4.24–4.31 (2H, m, H^22^), 7.16–7.21 (4H, t, H^37^), 7.43–7.47 (8H, m, H^17^, H^39^), 7.51–7.55 (4H, t, H^38^), 7.68–7.70 (4H, d, J = 8.4 Hz, H^36^), 8.08–8.10 (4H, d, J = 7.6 Hz, H^18^), 8.41–8.44 (2H, d, H^21^ (‐NH)), 8.52‐8.54 (2H, t, H^26^ (‐NH)). The ^31^P‐NMR, ^13^C‐NMR and MALDI‐TOF MS spectra were given Figure S35,37 and 38.

### 1.9 General Procedure for Synthesis of Click Compounds

Detailed synthesis methods are given for compound DPP‐Bnz‐Val‐click‐4‐Cl‐3‐CF_3_‐chalcone. Similar procedure was used in other compounds.

*DPP‐Bnz‐Val‐click‐4‐Cl‐3‐CF*_*3*_ *‐chalcone (**BVK1**)*: DPP‐Bnz‐Val‐PA compound and 4‐Cl‐3‐CF_3_‐Chalcone compound were dissolved in 1:1 THF‐water using CuSO_4_.5H_2_O‐Sodium ascorbate catalyst system in 1:2 mole ratio and stirred for 24 h at room temperature with the help of magnetic stirrer. The reaction was monitored by FT‐IR spectrum and terminated. After the reaction was stopped, the precipitate was separated by filtration and the filtrate was concentrated and precipitated in cold water. The precipitated solid was filtered and dried at room temperature to give a pure yellow colored product. Yield was 83%. FT‐IR (cm‐^1^, ATR): **ν**_**C‐H(Aromatic)**_: 3072; **ν**_**C═O**_: 1646 (C═O); **ν**_**C═C**_: 1603 (C═C). ^1^H NMR (400 MHz, DMSO‐d6, δ) (Figure S40): 8.81 (d, *J* = 3.1 Hz, 1H), 8.69 (d, *J* = 6.2 Hz, 2H), 8.43 (q, *J* = 10.1, 8.3 Hz, 4H), 8.25 (d, *J* = 9.1 Hz, 2H), 8.19–7.95 (m, 8H), 7.84 (m, 4H), 7.67 (dd, *J* = 20.7, 8.9 Hz, 6H), 7.58–7.26 (m, 14H), 7.24–7.09 (m, 4H), 6.82 (d, *J* = 8.6 Hz, 1H), 4.52–4.43 (m, 2H), 4.32 (dt, *J* = 18.1, 8.5 Hz, 4H), 2.14 (s, 2H), 0.94 (q, *J* = 9.5, 8.2 Hz, 12H). ^13^C NMR (101 MHz, DMSO) (Figure S41) δ: 19.42, 19.84, 30.44, 59.85, 115.20, 120.03, 121.01, 122.08, 124.71, 127.15, 128.28, 130.29, 132.14, 132.64, 134.45, 134.93, 137.17, 140.19, 141.99, 147.56, 152.59, 160.73, 166.18, 171.73, 188.26. ^31^P NMR (DMSO‐*d*6) (Figure S39): δ = 24.81–25.39 (2 P, d, PA), 9.12–10.28 (1 P, t, PB) A_2_B spin system. MALDI‐TOF MS (m/z) (Figure S42): theoritical 1753.38 m/z, found [M^+^]: 1753.46 m/z.

*DPP‐Bnz‐Val‐click‐4‐F‐Chalcone (**BVK2**)*: This compound was produced following the above‐described general approach and obtained as a white solid with a yield 65%. FT‐IR (cm‐^1^, ATR) (Figure S43): ν_C‐H(Aromatic)_: 3069; ν_C═O_: 1650 (C═O); ν_C═C_: 1597 (C═C). ^1^H NMR (400 MHz, DMSO‐d6, δ) (Figure S45): 8.80 (s, 1H), 8.70 (d, *J* = 7.1 Hz, 1H), 8.50–8.34 (m, 3H), 8.18–7.91 (m, 8H), 7.84–7.77 (m, 1H), 7.67 (dd, *J* = 13.1, 5.6 Hz, 3H), 7.59–7.25 (m, 10H), 7.16 (q, *J* = 8.8, 7.2 Hz, 3H), 4.48 (t, *J* = 5.4 Hz, 2H), 4.35 (t, *J* = 8.2 Hz, 2H), 2.26–2.05 (m, 2H), 0.93 (dd, *J* = 7.4, 3.7 Hz, 7H). ^13^C NMR (101 MHz, DMSO‐d6, δ) (Figure S46) 19.44, 19.84, 30.45, 34.60, 59.84, 116.33, 116.54, 120.03, 121.02, 122.11, 127.17, 128.27, 130.29, 130.98, 131.79, 131.93, 132.15, 137.47, 140.03, 143.76, 146.82, 147.57, 152.59, 162.75, 166.17, 171.72, 188.31. ^31^P NMR (DMSO‐*d*6) (Figure S44): δ = 24.81–25.38 (2 P, d, PA), 9.13–10.28 (1 P, t, PB) A_2_B spin system. MALDI‐TOF MS (m/z) (Figure S47): theoretical 1584.48 m/z, found [M+Na]: 1604.92 m/z.

*DPP‐Bnz‐Val‐click‐3,5‐difluoro‐chalcone (**BVK3**)*: This compound was produced following the above‐described general approach and obtained as a white solid with a yield 72%. FT‐IR (cm‐^1^, ATR) (Figure S48): ν_C‐H(Aromatic)_: 3071; ν_C═O_: 1644 (C═O); ν_C═C_: 1603 (C═C). ^1^H NMR (400 MHz, DMSO‐*d*_6_) (Figure S50) δ 8.76 (d, *J* = 48.5 Hz, 2H), 8.43 (dd, *J* = 24.5, 8.4 Hz, 2H), 8.10 (d, *J* = 9.0 Hz, 4H), 7.76 (d, *J* = 11.4 Hz, 2H), 7.64 (d, *J* = 7.5 Hz, 2H), 7.55–7.31 (m, 6H), 7.14 (s, 3H), 4.45 (s, 2H), 4.32 (s, 1H), 2.16–2.05 (m, 1H), 0.94–0.86 (m, 6H). ^13^C NMR (101 MHz, DMSO) (Figure S51): δ 19.40, 19.81, 30.45, 34.55, 59.77, 120.05, 121.00, 122.05, 124.87, 127.14, 128.26, 130.27, 130.38, 130.71, 131.15, 137.10, 138.88, 140.21, 142.22, 146.78, 147.60, 152.56, 161.95, 166.14, 171.68, 188.20. ^31^P NMR (DMSO‐*d*6) (Figure S49): δ = 24.79–25.37 (2 P, d, PA), 9.12–10.26 (1 P, t, PB) A_2_B spin system. MALDI‐TOF MS (m/z) (Figure S52): theoretical 1620.46 m/z, found **[**M]:1620.46 m/z.

*DPP‐Bnz‐Val‐click‐4‐OCH*_*3*_ *‐chalcone (**BVK4**)*: This compound was produced following the above‐described general approach and obtained as a light yellow solid with a yield 75%. FT‐IR (cm‐^1^, ATR) (Figure S53): ν_C‐H(Aromatic)_: 3069; ν_C═O_: 1644 (C═O); ν_C═C_: 1603 (C═C). ^1^H NMR (400 MHz, DMSO‐*d*_6_) (Figure S55): δ 8.87–8.61 (m, 2H), 8.47 (d, *J* = 8.3 Hz, 1H), 8.35 (d, *J* = 8.6 Hz, 1H), 8.20–7.96 (m, 4H), 7.93–7.82 (m, 2H), 7.76 (d, *J* = 15.4 Hz, 1H), 7.63 (d, *J* = 8.2 Hz, 2H), 7.43 (m, 6H), 7.07 (dd, *J* = 41.5, 10.8 Hz, 4H), 4.45 (t, *J* = 5.1 Hz, 2H), 4.30 (d, *J* = 8.3 Hz, 1H), 3.82 (d, *J* = 1.1 Hz, 3H), 2.17–2.09 (m, 1H), 0.91 (d, *J* = 7.1 Hz, 6H). ^13^C NMR (101 MHz, DMSO) (Figure S56): δ 19.42, 19.82, 30.43, 34.58, 55.86, 59.81, 114.14, 114.90, 120.00, 121.05, 122.04, 127.13, 127.71, 128.26, 130.27, 130.33, 130.77, 130.81, 130.85, 131.44, 132.12, 132.21, 137.79, 139.84, 145.02, 146.77, 147.55, 152.49, 152.57, 161.98, 166.14, 171.69, 188.21. ^31^P NMR (DMSO‐*d*6) (Figure S54): δ = 24.78–25.35 (2 P, d, PA), 9.11–10.26 (1 P, t, PB) A_2_B spin system. MALDI‐TOF MS (m/z) (Figure S57): theoretical 1608.55 m/z, found **[**M]:1608.50 m/z.

*DPP‐Bnz‐Val‐click‐3‐CF*_*3*_ *‐chalcone (**BVK5**)*: This compound was produced following the above‐described general approach and obtained as a grey‐colored solid with a yield 60%. FT‐IR (cm‐^1^, ATR) (Figure S58): ν_C‐H(Aromatic)_: 3071; ν_C═O_: 1641 (C═O); ν_C═C_: 1606 (C═C). ^1^H NMR (400 MHz, DMSO‐*d*_6_) (Figure S60) δ 8.76 (d, *J* = 45.2 Hz, 2H), 8.41 (dd, *J* = 18.7, 10.8 Hz, 3H), 8.30–7.93 (m, 5H), 7.94–7.76 (m, 2H), 7.67 (m, 3H), 7.57–7.28 (m, 6H), 7.15 (s, 3H), 4.45 (s, 2H), 4.35–4.22 (m, 1H), 2.13 (s, 1H), 0.97–0.89 (m, 7H). ^13^C NMR (101 MHz, DMSO) (Figure S61): δ 19.41, 28.28, 34.60, 59.82, 120.07, 122.08, 127.18, 128.27, 130.44, 137.25, 138.87, 140.16, 146.81, 147.61, 152.52, 166.17, 171.72, 188.36. ^31^P NMR (DMSO‐*d*6) (Figure S59): δ = 24.78–25.35 (2 P, d, PA), 9.08–10.24 (1 P, t, PB) A_2_B spin system. MALDI‐TOF MS (m/z) (Figure S62): theoretical 1684.50 m/z, found [M]:1684.37 m/z.

*DPP‐Bnz‐Val‐click‐4‐N(CH*_*3*_ *)*_*2*_ *‐chalcone (**BVK6**)*: This compound was produced following the above‐described general approach and obtained as a brown solid with a yield 77%. FT‐IR (cm‐^1^, ATR) (Figure S63): ν_C‐H(Aromatic)_: 3071; ν_C═O_: 1641 (C═O); ν_C═C_: 1606 (C═C). ^1^H NMR (400 MHz, DMSO‐*d*_6_) (Figure S65) δ 8.77 (s, 1H), 8.31 (d, *J* = 8.4 Hz, 1H), 8.07 (dd, *J* = 24.4, 8.5 Hz, 3H), 7.79–7.67 (m, 3H), 7.63 (d, *J* = 8.4 Hz, 2H), 7.44 (m, 5H), 7.14 (t, *J* = 5.6 Hz, 2H), 6.74 (d, *J* = 8.6 Hz, 2H), 4.45 (t, *J* = 5.6 Hz, 2H), 4.31 (s, 1H), 3.34 (d, *J* = 9.4 Hz, 6H), 2.18–2.09 (m, 1H), 0.92 (t, *J* = 7.3 Hz, 6H). ^13^C NMR (101 MHz, DMSO) (Figure S66) δ 19.45, 19.83, 30.40, 34.58, 40.43, 59.82, 112.17, 115.59, 116.09, 119.92, 121.02, 122.06, 122.35, 127.14, 128.25, 130.29, 130.39, 130.57, 130.72, 131.48, 132.10, 138.34, 139.55, 146.23, 146.74, 147.58, 152.56, 166.14, 171.70, 187.71. ^31^P NMR (DMSO‐*d*6) (Figure S64): δ = 24.78–25.35 (2 P, d, PA), 9.13–10.28 (1 P, t, PB) A_2_B spin system. MALDI‐TOF MS (m/z) (Figure S67): theoretical 1634.64 m/z, found **[**M]:1634.01 m/z.

### 1.10 Biological Evaluation

#### 1.10.1 In Vitro Cytotoxicity Studies

The human ovarian cancer cell line (A2780) and human colon cancer cell line (Caco‐2) were used in our study. Cells were fed with RPMI‐1640 and DMEM medium (A2780 and Caco‐2, respectively) and cultured in an incubator containing 5% CO_2_ at 37°C in a humidified environment. When the cells were confluent, they were detached from the flasks with trypsin‐EDTA solution and used in 3‐[4,5‐dimethylthiazol‐2‐yl]‐2,5‐diphenyltetrazolium bromide (MTT) analyses [35, 36, 37, 38] Cells were seeded in 96‐well plates (approximately 10.000 cells per well) for analysis. The following day, compounds at concentrations of 1–100 μM, prepared in 1% DMSO and 99% medium, were administered and incubated for 24 h. At the end of incubation, the wells were aspirated, and 0.5 mg/ml MTT solution was added to each well and incubated for 3 h. Afterwards, the optical densities of the cells were measured at 570 nm wavelength in ELISA device [39]. The average of the absorbance values of the control wells was accepted as 100% viable cells. The percentage viability was calculated by proportioning the absorbance values obtained from the solvent and agent‐treated wells to the control absorbance value. Statistical analyses were performed with IBM SPSS Statistics 22.0 software and *p* < 0.05 was considered statistically significant. LogIC50 values were calculated by Graphpad Prism 6 program according to MTT results [40, 41].

#### 1.10.2 In Vitro Genotoxicity (DNA Damage) Studies

The comet assay method was used in in vitro DNA damage (genotoxicity) studies. This method, also known as single‐cell gel electrophoresis, is widely used to determine DNA damage in mammalian cells. In this study, minor modifications were made to the neutral comet assay technique described by Devlin et al. [42, 43, 44]. Firstly, the slides were coated with 0.65% high‐melting agarose (HMA) and allowed to dry in the dark for 1 day. Cultured A2780 and Caco‐2 cells were incubated with compounds at a concentration of 100 µM for 24 h. After incubation, the cells were mixed with %1 low‐melting agarose at 42°C and spread on HMA‐coated slides, and the slides were kept at +4°C in the dark until solidified. The slides were then placed in freshly prepared cold lysis solution (2.5 M NaCl, 0.1 M EDTA, 0.01 M Tris base, %1 Triton‐X 100 and % 10 DMSO, pH:10) kept at +4°C in the dark for 1 h. After lysis, the slides were placed in a horizontal electrophoresis tank containing a cold neutral electrophoresis buffer (0.4 M Tris base, pH: 7.5) in the same orientation. The voltage of the tank was fixed at 25 V, the amperage was 300 mA, and electrophoresis was performed for 20 min. After electrophoresis, the slides were neutralized with buffer 3 times for 5 min at +4°C. The slides were stained with ethidium bromide and kept in the dark at +4°C for 20–30 min. The degree of DNA damage was assessed using a fluorescence microscope (Leica) and Comet IV software [45]. At least 25 cells were randomly counted from each slide, and changes in tail length, tail intensity, olive tail intensity, olive tail intensity, head length, and head intensity were determined.

#### 1.10.3 Tunel and ROS Studies

TUNEL In Situ Apoptosis Kit (Catalogue No: E‐CK‐A320, Elabscience, China) was used to determine apoptotic cells. Approximately 40.000 cells were seeded on 8‐well culture flasks and incubated for 24 h. Cells were treated with the compounds determined after MTT analyses for 24 h. At the end of the time, the medium was withdrawn, washed briefly with PBS and 4% paraformaldehyde prepared in PBS was added to each well and fixed at room temperature for 20 min. After fixation, the wells were aspirated and washed 3 times for 5 min with PBS. To increase cell permeability, 0.2% Triton‐X solution prepared in PBS was added and kept at 37°C for 10 min and washed with PBS 3 times for 5 min. TUNEL reagent prepared according to the kit protocol was pipetted into the wells and incubated at 37°C for 60 min. After incubation, the wells were aspirated and washed, then DAPI solution was added to stain the cell nuclei. After the protocol was completed, the slides were covered with coverslips and imaged under a ZEISS/Axio Scope.A1 (Germany) fluorescence microscope. The number of apoptotic cells was expressed as % in the images and differences between groups were analyzed using independent sample t‐test.

The level of intracellular reactive oxygen species (ROS) after treatments was determined using 2′,7′‐Dichlorofluorescein diacetate (DCFDA; D D6883, Sigma‐Aldrich). DCFDA is a cell‐permeable, nonfluorescent probe that is esterified intracellularly and converted to highly fluorescent 2′,7′‐dichlorofluorescein upon oxidation. In the study, approximately 40.000 cells for each cell were seeded into 8‐well cell slides and incubated for 24 h. After incubation, the wells were aspirated and 400 µL of cell feeding medium was added. Cells were treated with the compounds determined after MTT analyses for 2 h. The change in cellular ROS level was visualized under a fluorescence microscope (Leica DMi8, Germany) after incubation with 25 μM DCFDA for 30 min at 37°C [46]. Fluorescence intensities of the images were analyzed using Image software and independent sample t test was used for pairwise comparisons.

## 2 Results and Discussion

### 2.1 Chemistry

**Figure 1:**
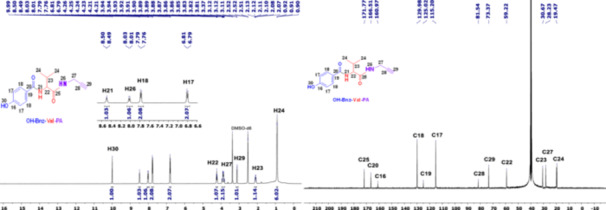
^1^H and ^13^C NMR spectra of OH‐Bnz‐Val‐PA (BV).

The singlet peak belonging to the ‐CO‐OH group at 12.58 ppm of the OH‐Bnz‐Val‐OH compound disappeared with the binding of the propargyl amine compound to the structure, in addition, the triplet resonance of the new ‐NH proton at 8.01–8.03 ppm is detected in the ^1^H‐NMR spectrum of the OH‐Bnz‐Val‐PA compound in Figure 1. In addition, the aliphatic proton peaks at 3.11–3.12 (1H, s, H29) and 3.81–3.94 (2H, m, H27) ppm in the structure of the formed propargyl amine compound can be given as other evidence that the compound is formed. The matching of the integral heights and the observation of the expected peaks indicate that the structure is formed. Similar conditions are also observed in the carbon peaks of the protons mentioned above in the ^13^C‐NMR spectrum. The aliphatic carbons 27, 28 and 29 in the structure of the propargyl amine compound are observed at 28.23 (C27), 81.54 (C28) and 73.37 (C29) ppm, respectively.

**Figure 2:**
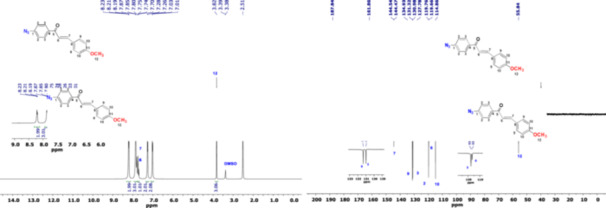
^1^H and ^13^C NMR spectra of *4‐OCH*_*3*_ *‐chalcone‐N*_*3*_ *(**K4**)*.

The most characteristic peak seen in the ^1^H NMR of the 4‐OCH_3_‐N_3_‐chalcone compound is the methoxy (‐OCH_3_) singlet peak at 3.82 ppm, and the presence of the peak at 7.72 ppm belonging to proton 6 among the olefinic peaks shows that the structure is formed in Figure 2. In addition, this proton with a J value of 15.3 Hz indicates that the structure is trans‐isomerized.

**Figure 3:**
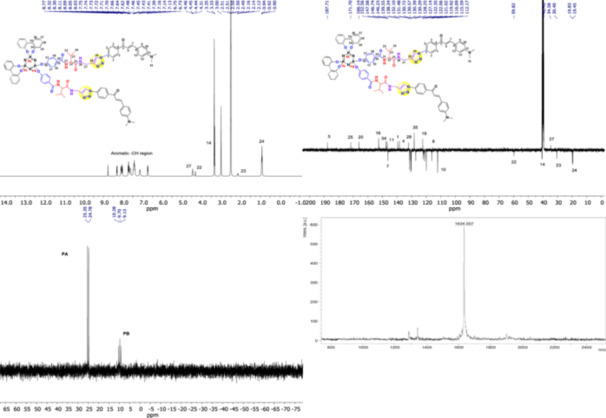
^1^H, ^13^C, ^31^P NMR and MALDI‐TOF MS spectra of *DPP‐Bnz‐Val‐click‐4‐N(CH*_*3*_ *)*_*2*_ *‐chalcone (**BVK6**)*.

The ^31^P‐NMR spectrum of the starting material DPP compound displayed that the peak at 28.71–29.69 ppm (1 P, t, PB(PCl_2_)), which was seen as a triple peak with P‐Cl groups in the DPP compound, shifted to 9.13–10.28 ppm (1 P, t, PB((C_23_H_34_O_7_N_3_)_2_) with the binding of ***BVK6*** to the phosphazene ring. The phosphorus peak with P‐Cl groups was not observed at ^31^P‐NMR spectrum in Figure 3. The phosphorus peaks associated with biphenyl groups (PA) were shifted from 19.22 to 19.71 ppm in the DPP compound (2 P, d, PA((‐C_12_H_8_O_2_)_2_) In the ***BVK6*** compound, it was shifted to 24.78–25.35 ppm (2 P, d, PA((‐C_12_H_8_O_2_)_2_) by chalcone bonding as a result of click reaction to the phosphazene ring. The peaks belonging to the phosphors in the DPP compound were not observed in the ^31^P‐NMR spectrum of DPP‐Bnz‐Val‐click‐4‐N(CH_3_)_2_‐chalcone. When the ^1^H NMR spectrum of DPP‐Bnz‐Val‐click‐4‐N(CH_3_)_2_‐chalcone is examined, aliphatic peaks belonging to ‐CH_3_, ‐CH (6H at 0.92 ppm, ‐CH at 2.14 ppm, ‐CH at 4.31 ppm) and ‐CH_2_ protons belonging to the propargyl moiety of valine amino acid (‐CH_2_ at around 4.45 ppm) are clearly distinguishable. The number of other olefinic and aromatic protons in the compound is consistent with its structure. The ^13^C NMR spectrum of the compound shows the chalcone structure at 187.71 ppm, the amide carbonyl carbon peak at 171.70 ppm, the ‐CH_2_ carbon of the propargyl group at 34.58 ppm, and the ‐CH3 carbon peaks of the valine amino acid at 19.83 and 19.45 ppm. The number of other aromatic ‐C and ‐CH carbons is consistent with the structure. The theoretically calculated molecular weight of the DPP‐Bnz‐Val‐click‐4‐N(CH_3_)_2_‐chalcone compound is 1634.64 g/mol. In the MALDI‐TOF MS spectrum, it corresponds to 1634.00 [M]. This is one of the other evidence showing that the compound was formed.

### 2.2 Biological Activity

It is known that the groups bound to phosphazene compounds exhibit different physical, chemical and biological properties depending on their properties. In the literature, various studies have been carried out to determine the biological activities of phosphazene compounds in anticancer, antibacterial and DNA research [10, 47, 48, 49]. For example, in one study, a biodegradable phosphazene trimer‐doxorubicin conjugate was synthesized by grafting cyclotriphosphazene methoxy‐poly(ethylene glycol) and a tumor‐specific tetrapeptide and was reported to exhibit high in vitro cytotoxicity against the leukemia L1210 cell line [50]. In another study, hexachlorocyclotriphosphazene and thiosemicarbazone compounds were synthesized and found to exhibit moderate to high antiproliferative activities against human breast and ovarian cancer cell lines [50]. In a 2015 study, reactions of chalcone compounds with phosphazene compounds were carried out using aldehydes with different side groups and these compounds were found to exhibit high cytotoxic activity against prostate cancer cell lines [47]. In the same year, cyclotriphosphazene ligands bearing 3‐oxypyridine groups and Ru (II) complexes were obtained and these complexes were found to exhibit high cytotoxic and antimicrobial activities against cancer cell lines [51]. In a 2019 study, it was reported that coumarin‐phosphazene compounds have antitumour activity against human breast cancer cell lines [52]. In a 2022 study, the structural, steoregenic and biological activities of phosphazene derivatives bearing morpholine, piperidine and pyrolidine groups were investigated and these compounds were found to be particularly effective against P. aeruginosa [53]. In 2022, phosphazene compounds bearing novel anthraquinone groups were shown to selectively reduce cancer cell viability and induce apoptosis against non‐small cell lung carcinoma cell lines [54]. Finally, in 2023, phosphazene derivatives containing dipeptide structures were synthesized and found to cause cell death through DNA damage and exhibit remarkable effects, especially in A2780 cell lines.

### 2.3 In Silico Analysis

A molecular docking study was conducted to investigate the biological behavior and potential enzyme‐substrate interactions of cyclotriphosphazene derivative drug candidates. The optimized structures of cyclotriphosphazene derivatives were optimized using the B3LYP/6‐31 G(d,p) basis set with the density functional theory method in Gaussian16 software [Reference]. Crystal structures of the enzymes were obtained from the Protein Data Bank [55]. Bcl‐2 (PDB: 4LVT) promotes tumor growth by inhibiting apoptosis [56]. p53 initiates apoptosis in response to DNA damage; its mutant form (PDB: 5O1F) loses its function and contributes to tumor development, while wild type p53 (PDB: 2AC0) suppresses cancer [57, 58]. Caspase‐3 (PDB: 1RE1) cleaves cells during the final stage of apoptosis [59]. VEGFR2 (PDB: 1YWN) nourishes tumors by forming blood vessels [60]. Compounds that bind to DNA (PDB: 1FJ5) inhibit tumor growth by triggering apoptosis [61, 62]. Proteins were optimized with AutoDock Tools 1.5.6 software, and Kollman charges were added after adding polar hydrogens [63]. Molecular docking was performed with AutoDock Vina [64] and the results were visualized with UCSF Chimera software [65].

In this study, the potential of **BV** and **BVK2** molecules as targeted inhibitors in cancer treatment has been comprehensively evaluated using molecular docking analyses. The characteristic properties of cancer cells, such as their ability to grow more uncontrollably than healthy cells and their ability to evade apoptosis by desensitizing to environmental signals, result from various molecular mechanisms triggered by genetic mutations. These changes include overexpression of the Bcl‐2 protein, which suppresses cell death, loss of function of the tumor suppressor p53 gene, and activation of the VEGFR2 receptor, which initiates the angiogenesis process to meet the oxygen and nutrient requirements of tumors. The activation of VEGFR2, in particular, plays a critical role in enabling tumors to grow by forming new blood vessels. This *in silico* analysis reveals how **BV** and **BVK2** interact with these proteins and how their structural features make them potential therapeutic agents.

**Figure 4:**
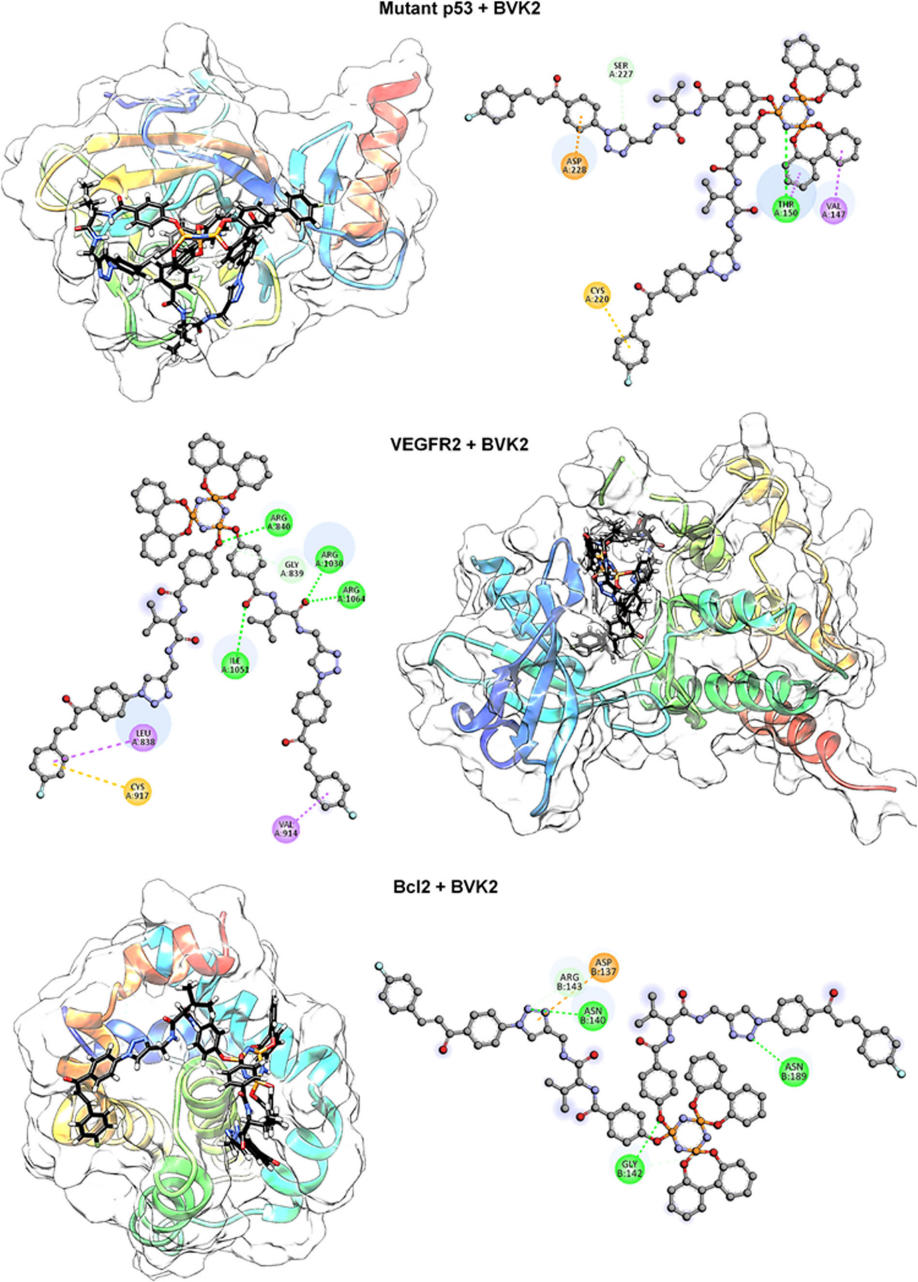
Appearance of **BVK2** bound to mutant p53, VEGFR2 and Bcl‐2 and its intermolecular interactions with the residues.

Molecular docking analyses conducted in this context have revealed that **BVK2** exhibits high selectivity against target proteins such as Bcl‐2, mutant p53, and VEGFR2 in Figure 4. The large volume, numerous rotatable bonds, and diversity of functional groups possessed by **BVK2** make it a molecule with high binding affinity. In particular, the p‐fluorobenzene group in the chemical structure of **BVK2** fits perfectly into the hydrophobic pockets of proteins, while the nitrogen and oxygen atoms on the phosphazene group form strong bonds with hydrogen bond donor amino acids.

The high binding score of −11.4 kcal/mol between **BVK2** and the Bcl‐2 protein indicates that it can act as a selective inhibitor on this protein. **BVK2** forms hydrogen bonds with Gly142, Asn140, and Arg143 amino acids on the Bcl‐2 protein while forming π‐anion interactions with Asp137 and multiple interactions such as P–O···Gly142 with triazole groups. The combination of these hydrophobic and hydrophilic interactions enhances the potential of **BVK2** to block the antiapoptotic effect of Bcl‐2 and promote apoptosis in cells. In particular, the binding of triazole rings to amino acids in the active sites of Bcl‐2 allows for versatile interactions due to the structural flexibility of **BVK2**, thus contributing to the regulation of apoptosis. The binding affinities of **BV**, **BVK2,** Docetaxel, and Tamoxifen to Bcl‐2, Caspase‐3, Mutant p53, Wild type p53, VEGFR2, and DNA are summarized in Table 1.

**Table 1:** Binding affinities of BV, BVK2, Docetaxel, and Tamoxifen to Bcl‐2, Caspase‐3, Mutant p53, Wild type p53, VEGFR2, and DNA.

	Binding affinity (kcal/mol)			
	BV	BVK2	Docetaxel	Tamoxifen
Bcl‐2	−8.4	−11.4	−7.3	−7.4
Caspase‐3	−10.1	−10.0	−8.2	−6.6
Mutant p53	−8.4	−9.2	−6.7	−9.1
Wild type p53	−9.3	−7.9	−7.2	−8.2
VEGFR2	−9.7	−11.9	−7.9	−7.0
DNA	−8.4	−7.3	−7.7	−6.6

Mutant p53 protein is a common mutation in cancer cells and plays a critical role in tumor growth and metastasis. **BVK2** exhibits strong interactions with this protein with a binding score of −9.2 kcal/mol. **BVK2** forms hydrogen bonds with Thr150 and Ser227 amino acids, while forming π‐anion interactions with Asp228 and π‐sigma interactions with Val147, ensuring structural integrity. In addition, the p‐fluorobenzene group interacts with Cys220 through π‐sulfur interactions, adapting to the binding pockets of mutant p53. Such diverse and strong interactions of **BVK2** on mutant p53 indicate the potential to restructure cell cycle control and apoptosis mechanisms, allowing it to be considered as a targeted inhibitor in cancer therapy.

The very high binding score of −11.9 kcal/mol between **BVK2** and the VEGFR2 protein suggests that it can exert a strong inhibitory effect on this receptor. **BVK2** forms hydrogen bonds with Arg840, Gly839, Arg1030, and Arg1064 amino acids on VEGFR2, while forming π‐sigma interactions with hydrophobic amino acids such as Val914 and Leu838. Moreover, the p‐fluorobenzene group strongly binds to the active sites of VEGFR2 through π‐sulfur interactions with Cys917. This binding mode enhances the ability of **BVK2** to inhibit angiogenesis in tumors, limiting the access of tumors to oxygen and nutrients. Such binding models of **BVK2** indicate that it can function as a versatile agent in cancer therapy, slowing tumor growth and preventing metastasis.

**Figure 5:**
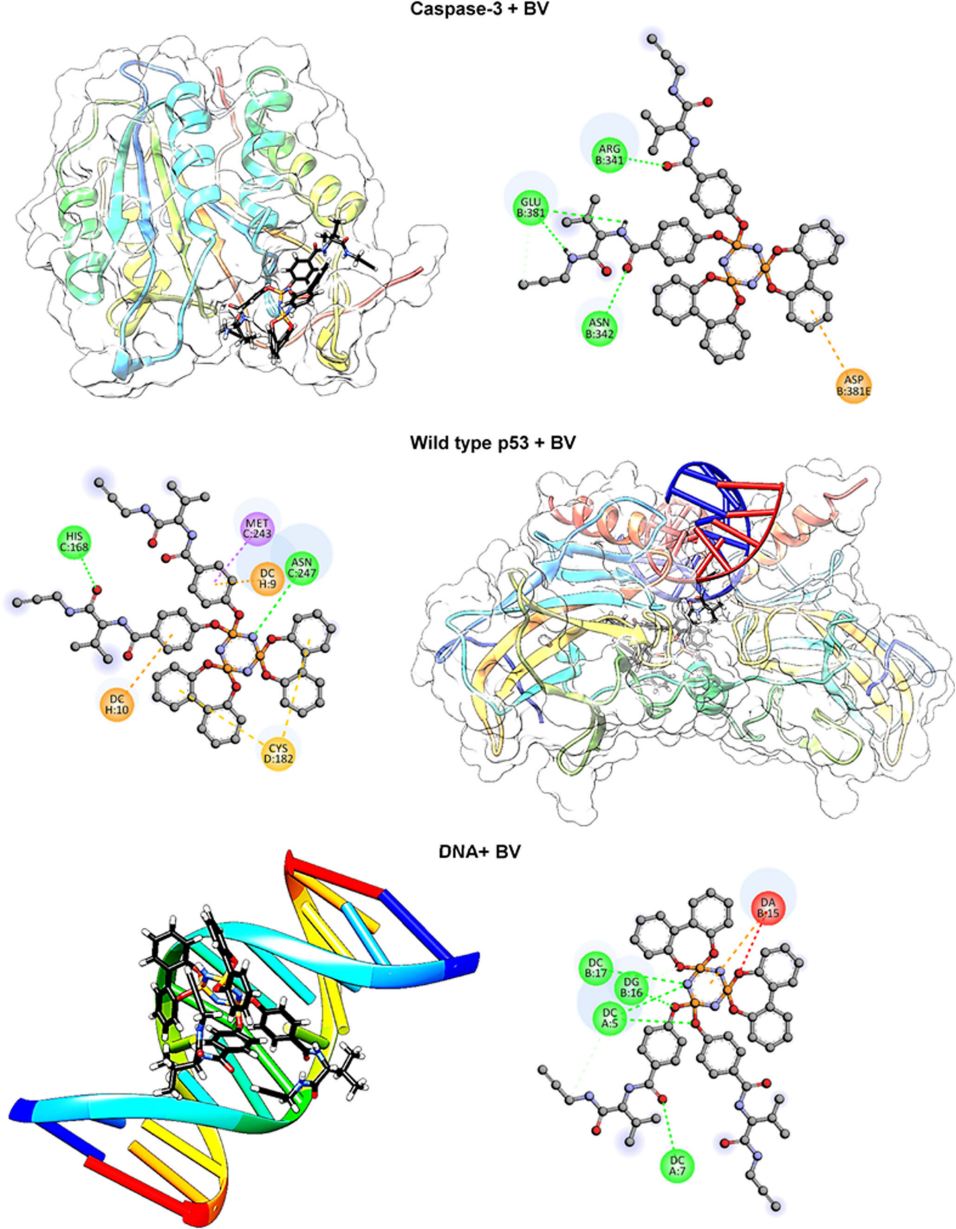
Appearance of **BV** bound to mutant caspase‐3, wild type‐p53 and DNA and its intermolecular interactions with the residues.

Molecular docking analyses of the **BV** molecule have revealed strong binding scores and significant amino acid interactions with cancer‐related proteins such as Caspase‐3, wild‐type p53, and DNA in Figure 5. With a binding score of −10.1 kcal/mol against the Caspase‐3 protein, **BV** exhibits the potential to trigger the apoptosis mechanism through specific interactions established with this protein. While forming hydrogen bonds with Arg341 and Asn342 amino acids in the Caspase‐3 protein, its C═O···π‐anion interaction with Glu381 reveals a high‐affinity binding model for **BV** to induce apoptosis. These interactions provide a significant clue that **BV** can suppress tumor growth by promoting programmed cell death in cancer cells.

The fact that **BV** shows a binding score of −9.3 kcal/mol with the wild type p53 protein suggests that it may also be effective in the tumor suppressor p53 pathway. **BV** forms hydrogen bonds with Asn247 and His168 amino acids on p53 while forming π‐sigma interactions with Met243 and π‐sulfur interactions with Cys182. These interactions highlight the potential of **BV** to interfere with the p53 pathway, arrest the cell cycle in cancer cells, and achieve tumor suppression.

Furthermore, the fact that **BV** has a binding score of −8.4 kcal/mol with DNA indicates that it has the potential to damage genetic material in cancer cells by directly interacting with DNA. The **BV** molecule forms hydrogen bonds with bases such as P–O···DC5 and P–N···DC5 on DNA while forming attractive electrostatic interactions with DA15 and DC9 bases. The fact that the lengths of these bonds are between 1.93 and 2.89 Å suggests that **BV** can bind tightly to the DNA double helix and trigger structural changes. These interactions emphasize that **BV** can induce apoptosis in cancer cells by directly damaging genetic material.

When compared to reference drugs such as docetaxel and tamoxifen, **BV** and **BVK2** have been shown to exhibit higher binding affinities. While tamoxifen's high selectivity for mutant p53 supports the possibility that this drug could be an effective treatment for cancer types with frequent p53 mutations, docetaxel's selective interactions with Caspase‐3, in particular, can be considered as an important finding in terms of inducing apoptosis. However, the structural advantages and high binding scores of **BV** and **BVK2** make them more promising targeted treatment options than existing therapeutic agents, with potentially higher efficacy and lower toxicity.

In conclusion, the in vitro and in silico results obtained for **BV** and **BVK2** indicate that they are promising candidates as targeted inhibitors with multifaceted effects in cancer treatment. In particular, **BVK2**'s large volume, flexible structure, and rich functional group diversity give it the potential to affect two fundamental mechanisms of cancer: apoptosis and angiogenesis. The strong binding affinity of BV to Caspase‐3 suggests its role in activating apoptotic pathways, which aligns with the observed reduction in cancer cell viability. Additionally, its interaction with wild‐type p53 indicates potential involvement in cell cycle arrest, further supporting the cytotoxic effects seen in treated cancer cells. Strong binding to Bcl‐2 and mutant p53 could suggest the compounds’ potential to inhibit apoptosis regulation, while binding to VEGFR2 may support their ability to hinder angiogenesis. These findings support the efficacy of **BV** and **BVK2** against biological targets and suggest that these molecules should be evaluated in more detail in preclinical studies.

#### 2.3.1 In Vitro Cytotoxicity

The % changes in cell viability of the compounds against ovarian (A2780) and colon (Caco‐2) cancer cell lines were determined by MTT assay. The % changes in cell viability rates caused by 1, 5, 25, 50 and 100 μM concentrations of the compounds were examined and % cell viability results and LogIC_50_/IC_50_ values are given in Table 2.

**Table 2:** % cell viability and LogIC50 (concentration causing 50% inhibition) values (µM) of the compounds in A2780, Caco‐2 cell lines (Bold: *p < 0.05 compared to control group, #*p* < 0.05 compared to solvent group) reference drug: TX: Tamoxifen, DX: Docetaxel.

	Control	Solvent	1 µM	5 µM	25 µM	50 µM	100 µM	IC_50_/LogIC_50_ (µM)
**% Cell Viability in A2780 Cell Lines**								
**BV**	100 ± 3.829	93.25 ± 2.681	92.15 ± 3.529	68.89 ± 1.735	68.59 ± 1.206	**59.77** ± 2.409*#	**54.99** ± 2.629*#	75,94/1,88
**BVK1**	100 ± 2.573	92.95 ± 3.956	**64.72** ± 2.883*	**59.91** ± 1.791*	**54.82** ± 3.007*	**51.79** ± 2.034*#	**36.02** ± 0.989*#	34,25/1,535
**BVK2**	100 ± 2.573	92.95 ± 3.956	**70.84** ± 3.217*	**63.24** ± 2.267*	**57.39** ± 2.229*#	**59.05** ± 2.301*#	**56.85** ± 2.287*#	61,98/1,792
**BVK3**	100 ± 3.816	92.15 ± 2.28	103.1 ± 4.763	102 ± 2.861	95.94 ± 2.246	96.96 ± 1.614	93.32 ± 4.196	—
**BVK4**	100 ± 3.816	92.15 ± 2.28	**77.45** ± 1.088*	**60.46** ± 2.094*	**55.99** ± 3.207*	**50.75** ± 1.808*#	**44.76** ± 2.141*#	40,79/1,611
**BVK5**	100 ± 5.409	96.79 ± 4.557	98.67 ± 8.481	94.72 ± 5.11	105.2 ± 2.172	95.09 ± 2.592	101.6 ± 4.358	—
**BVK6**	100 ± 5.409	96.79 ± 4.557	101.8 ± 2.231	101.9 ± 4.18	91.87 ± 3.963	93.92 ± 3.492	97.07 ± 3.522	—
**K4**	100 ± 4.199	101.3 ± 2.585	95.68 ± 1.701	90.08 ± 2.684	81.29 ± 2.684	**68.11** ± 2.684*#	**60.78** ± 2.684*#	125,8/2,1
**K6**	100 ± 7.575	98.45 ± 5.315	98.2 ± 2.265	97.97 ± 4.534	86.3 ± 4.789	88.19 ± 2.113	89.63 ± 3.933	—
**TX**	100 ± 3.478	96.93 ± 3.802	87.32 ± 3.478	88.27 ± 2.852	82.5 ± 5.46	**78.6** ± 5.392*	**38.91** ± 6.322*#	98,96/1,995
**DX**	100 ± 4.199	101.3 ± 2.585	98.54 ± 4.199	99.85 ± 4.052	**79.35** ± 4.052*	**70.67** ± 1.113*#	**56.7** ± 2.443*#	122/2,086
**% Cell Viability in Coco‐2 Cell Lines**								
**BV**	100 ± 2.186	96.42 ± 1.62	96.97 ± 2.104	79.83 ± 2.712	**74.6** ± 2.323*	**68.29** ± 2.622*#	**66.35** ± 0.678*#	127,2/2,104
**BVK1**	100 ± 2.293	96.79 ± 1.517	94.49 ± 2.39	91.42 ± 1.863	90.67 ± 2.351	87.91 ± 3.022	**79.72** ± 1.492*#	357,9/2,554
**BVK2**	100 ± 2.293	96.79 ± 1.517	92.26 ± 3.351	90.05 ± 2.675	88.73 ± 2.584	**79.56** ± 1.986*	**69.64** ± 1.669*#	208,9/2,32
**BVK3**	100 ± 3.131	94.67 ± 1.576	98.31 ± 3.52	93.17 ± 3.791	100.2 ± 3.516	93.3 ± 3.229	94.31 ± 3.687	—
**BVK4**	100 ± 3.131	94.67 ± 1.576	93.27 ± 3.928	80.73 ± 3.427	80.31 ± 2.622	**70.75** ± 3.446*#	**69.43** ± 2.313*#	154/2,188
**BVK5**	100 ± 4.153	92.81 ± 1.074	103.4 ± 3.975	92.18 ± 1.916	84.19 ± 2.491	84.69 ± 2.821	**82.82** ± 2.011*	343,2/2,536
**BVK6**	100 ± 4.153	92.81 ± 1.074	93.14 ± 3.354	93.33 ± 2.658	85.55 ± 2.398	83.48 ± 2.864	**71.74** ± 1.582*#	232,9/2,367
**K4**	100 ± 2.633	91.46 ± 2.86	74.19 ± 5.906	65.48 ± 3.073	**58.39** ± 2.907*	**57.4** ± 3.817*#	**51.43** ± 0.957*#	125,8/2,100
**K6**	100 ± 1.955	98.11 ± 3.245	99.6 ± 3.3	100.7 ± 2.613	100.6 ± 2.943	97.84 ± 3.393	97.88 ± 2.998	—
**TX**	100 ± 2.313	94.4 ± 3.335	99.35 ± 3.156	100.4 ± 1.889	87.95 ± 3.035	88.74 ± 3.925	**62.95** ± 2.716*#	212,9/2,328
**DX**	100 ± 2.633	91.46 ± 2.86	80.59 ± 3.015	**73.52** ± 2.057*	**74.2** ± 3.526*	**74.53** ± 3.455*	**73.63** ± 3.681*	165,8/2,22

*Note:* The significant doses as a result of statistical analysis are indicated in bold.

**Figure 6:**
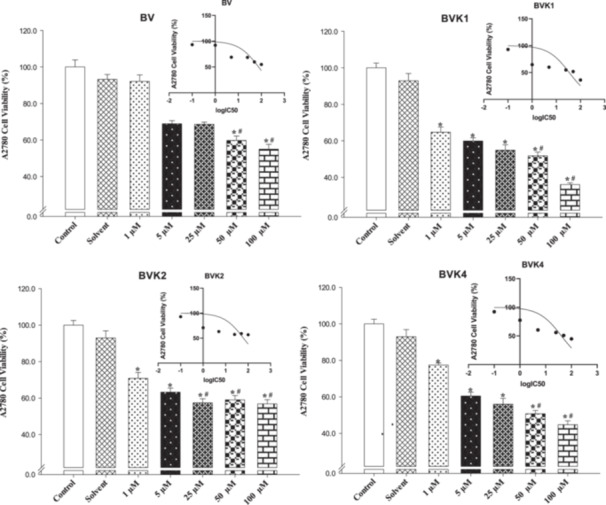
The cell viability (%) and LogIC_50_ values of compounds lines with high activity against A2780 cell lines. The changes in the cell viability (%) caused by compounds are compared with the control data. Each data point has an average of 10 viability (*p < 0.05).

In A2780 cells, high doses (50–100 µM) of BV, BVK1, BVK2, BVK4 compounds decreased the viability levels by approximately 30% to 65%. In addition, VPA compound showed a partially cytotoxic effect and inhibited the viability level by approximately 25% to 15% compared to the control groups (Table 2, Figure 6).

**Figure 7:**
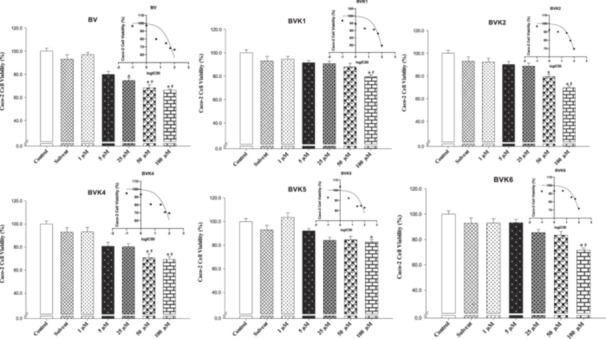
The cell viability (%) and LogIC_50_ values of compounds lines with high activity against Caco‐2 cell lines. The changes in the cell viability (%) caused by compounds are compared with the control data. Each data point has an average of 10 viability (*p < 0.05).

In Caco‐2 cells, BV, BVK1, BVK2, BVK4, BVK5, BVK6 compounds showed partially cytotoxic effect and inhibited the viability level by 10% to 35% compared to control groups (Table 2, Figure 7). The newly synthesized compounds were found to cause variable levels of cytotoxicity in different cancer cell lines. This difference in effect between cell lines is normal considering the heterogeneity of cancer cells. Following the MTT results, substances with common effects were identified for use in the other stages of the study. Phosphazene compounds BV, BVK2, which are effective in cancer cell lines, were investigated in other biological effect studies. Standard agents DX and TX were also included in these studies.

#### 2.3.2 In Vitro Genotoxicity

**Figure 8:**
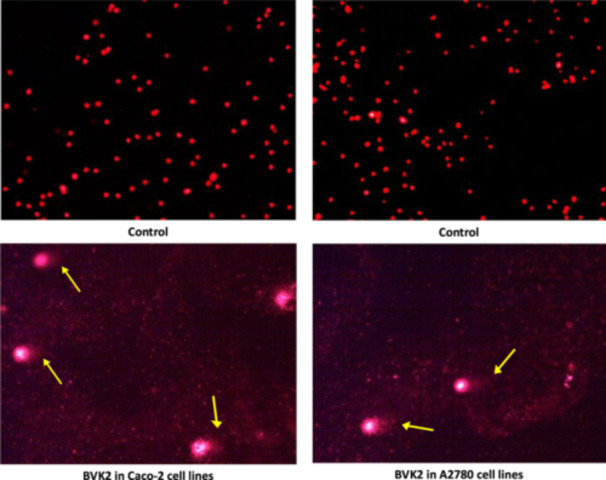
Sample images of BVK2 from cancer cells within the scope of the Comet Assay trials experiments (Yellow arrows indicate cells with DNA damage and comet image obtained; X10).

To investigate whether the decrease in cell viability was due to DNA damage mechanism, tail length (TL), tail intensity (TI), olive tail intensity (OTI), head length (HL) and head intensity (HI) parameters were examined by comet assay analysis (Figure 8). The presence and rate of DNA damage with the changes in these parameters were determined and presented in Table 3. It was determined that the compounds caused changes in these parameters, which were statistically significant.

**Table 3:** Changes in TL, TI, OTI, HD and HI values 24 h after application of cytotoxic compounds to A2780, Caco‐2 cell cultures (*p < 0.05 compared to control group, #*p* < 0.05 compared to solvent group).

	Tail length (TL)	Tail intensity (TI)	Olive Tail İntensity (OTI)	Head Length (HL)	Head Intensity (HI)
**DNA Damage in A2780 Cell Lines**					
**Control**	16 ± 5.29	67.19 ± 31.12	2.23 ± 1.10	112.24 ± 12.05	1941.62 ± 426.35
**Solvent**	17.92 ± 4.80	73.04 ± 32.1713	2.29 ± 1.04	118.24 ± 15.63	2166.84 ± 590.47
**K4**	147.8 ± 190.80*^#^	1791.95 ± 3426.43*^#^	63 ± 103.61*^#^	92.08 ± 15.97*^#^	1375.77 ± 520.06*^#^
**K6**	42.16 ± 14.27*^#^	220.03 ± 84.91*^#^	9.97 ± 3.98*^#^	89.44 ± 13.38*^#^	1291.45 ± 398.97*^#^
**BV**	53.72 ± 129.73*^#^	455.57 ± 1496.61*^#^	19.01 ± 64.83*^#^	98 ± 12.67*	1553.62 ± 466.37*^#^
**BVK2**	30.12 ± 56.67	151.12 ± 246.37	8.12 ± 21.52	94.96 ± 13.57*^#^	1454.32 ± 419.15*^#^
**TX**	98.48 ± 137.14*^#^	1076.3 ± 2528.41*^#^	38.02 ± 76.30*^#^	92.24 ± 10.89*^#^	1386.10 ± 375.08*^#^
**DX**	89.2 ± 113.84*^#^	600.67 ± 1146.99*^#^	33.45 ± 54.76*^#^	81.28 ± 14.71*^#^	965.34 ± 380.77*^#^
**DNA Damage in Caco‐2 Cell Lines**					
**Control**	32.64 ± 8.73	239.71 ± 94.89	8.88 ± 4.99	104.96 ± 22.88	1827.41 ± 806.06
**Solvent**	36 ± 14.38	219.78 ± 110.27	6.07 ± 3.05	141.04 ± 26.30	3144.05 ± 1211.22
**K4**	183.88 ± 147.90*^#^	2062.63 ± 2356.65*^#^	80.41 ± 83.20*^#^	106.4 ± 19.14^#^	1730.73 ± 736.58^#^
**K6**	101.72 ± 98.75*^#^	953.54 ± 1515.27*^#^	36.37 ± 48.53*^#^	104.24 ± 20.58^#^	1750.47 ± 659.57^#^
**BV**	132.88 ± 133.57*^#^	1301.15 ± 1985.37*^#^	46.16 ± 57.64*^#^	110.32 ± 27.05^#^	1963.98 ± 1172.05^#^
**BVK2**	114.6 ± 107.27*^#^	1297.44 ± 1585.79*^#^	43.49 ± 50.23*^#^	122.24 ± 45.24*^#^	2513.51 ± 1837.70^#^
**TX**	96.052 ± 110.42*	998.46 ± 1815.29	35.05 ± 56.10	113.32 ± 30.95*^#^	2124.68 ± 1406.53^#^
**DX**	113.96 ± 135.13*^#^	985.92 ± 1496.85	43.11 ± 71.79^#^	108.56 ± 23.22^#^	1922.69 ± 868.12^#^

DNA damage studies of the compounds in human ovarian (A2780) and human colon cancer (Caco‐2) cell lines were carried out. Tail length (TL), Tail intensity (TI), Olive Tail Intensity (OTI), Head length (HL), and Head intensity (HI) parameters were analysed at the dose that provided 60–70% viability.

The compounds treated with A2780 cells caused significant changes in DNA damage parameters compared to the control and solvent groups (Table 3; *p* < 0.05). The test compounds increased the tail length, tail density, tail moment parameters, which are considered as DNA damage indicators, compared to the control groups. In addition, head diameter and density decreased. These results revealed that DNA damage in A2780 cells increased after treatment with compounds.

The level of DNA damage in Caco‐2 cells after treatment with the compounds is summarized in Table 3. Accordingly, all test compounds except **K4** caused an increase in tail length (*p* < 0.05). All test compounds decreased the head diameter and density parameters compared to the solvent group (*p* < 0.05).

#### 2.3.3 Tunel and ROS Results

**Figure 9:**
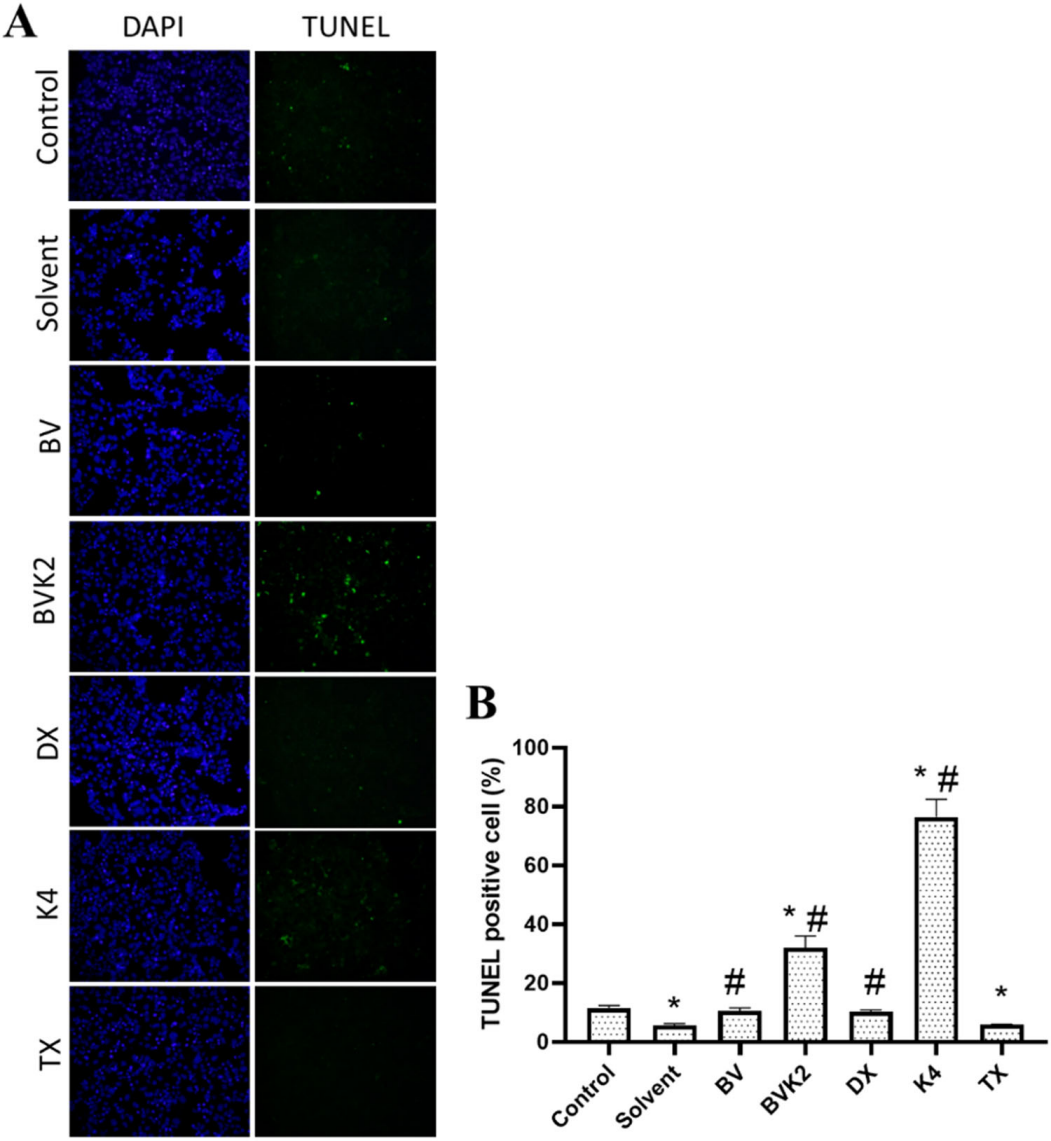
TUNEL analysis results in Caco‐2 cells after treatments. A; After treatment with the compounds, the samples were visualized under fluorescence microscope. Cell nuclei were stained with DAPI. TUNEL staining (green fluorescence) was performed for apoptotic cells. B; The percentage of TUNEL‐positive cells was also calculated and shown in graphs. *p < 0.05 compared to control group, ^#^ *p* < 0.05 compared to solvent group. (100X).

The effects of compounds treated with Caco‐2 cells on apoptotic cell death are shown in Figure 9. Accordingly, BVK2 and K4 compounds increased apoptotic cell death compared to control and solvent group (*p* < 0.05). The number of apoptotic cells was higher in BV and DX treated groups compared to the solvent group.

**Figure 10:**
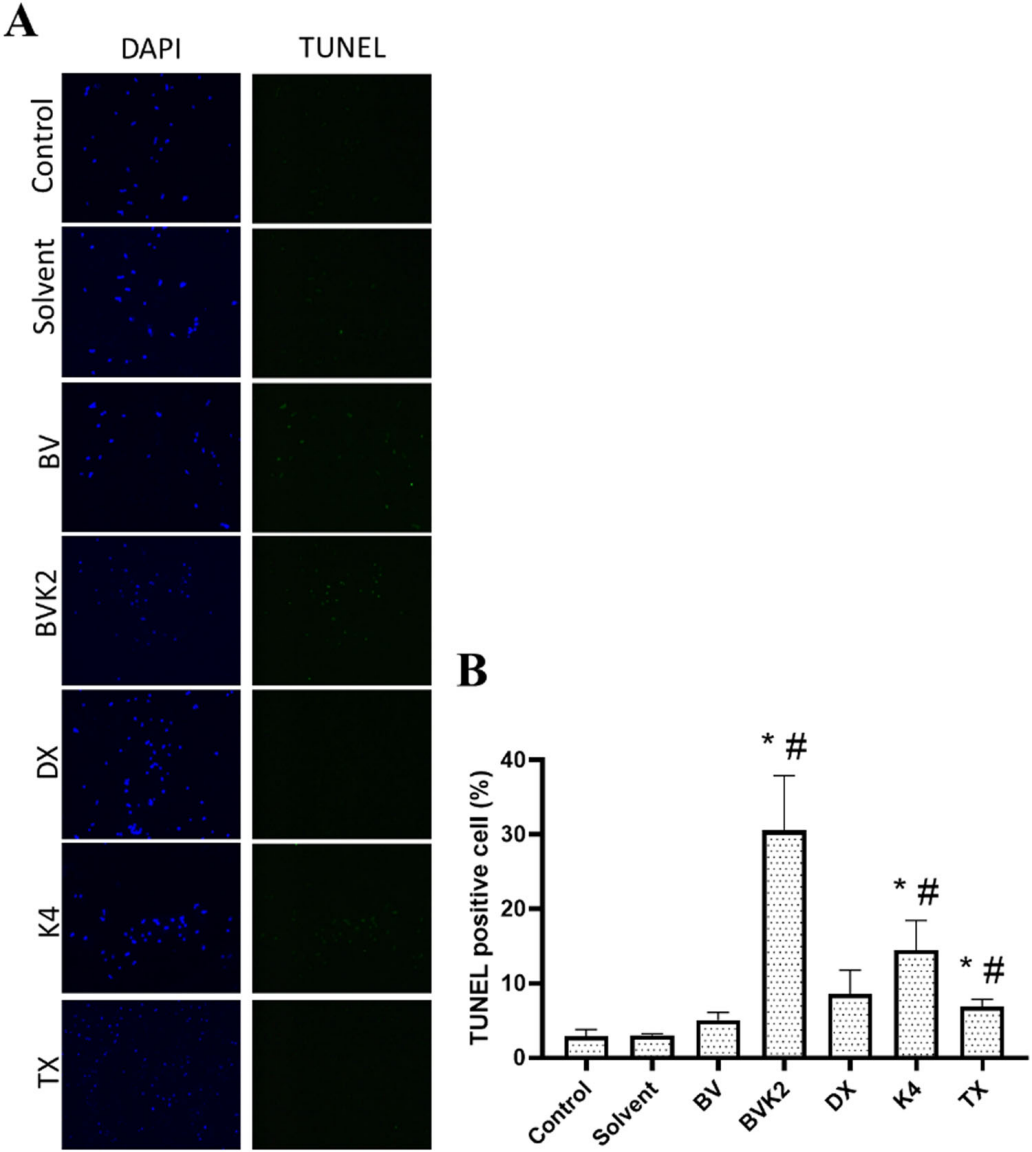
TUNEL analysis results in A2780 cells after treatments. A After treatment with compounds, the samples were visualized under fluorescence microscope. Cell nuclei were stained with DAPI. TUNEL staining (green fluorescence) was performed for apoptotic cells. B The percentage of TUNEL‐positive cells was also calculated and shown in graphs. *p < 0.05 compared to control group, ^#^ *p* < 0.05 compared to solvent group. (100X).

BVK2, K4 compounds applied to A2780 cells significantly increased the number of TUNEL‐positive cells compared to the solvent group (*p* < 0.05). In addition, TX treatment caused an increase in the number of apoptotic cells compared to the control groups (*p* < 0.05). No significant change was observed in the number of TUNEL‐positive cells after the application of other compounds. Visualizations of the treatments and group comparisons are presented in Figure 10.

**Figure 11:**
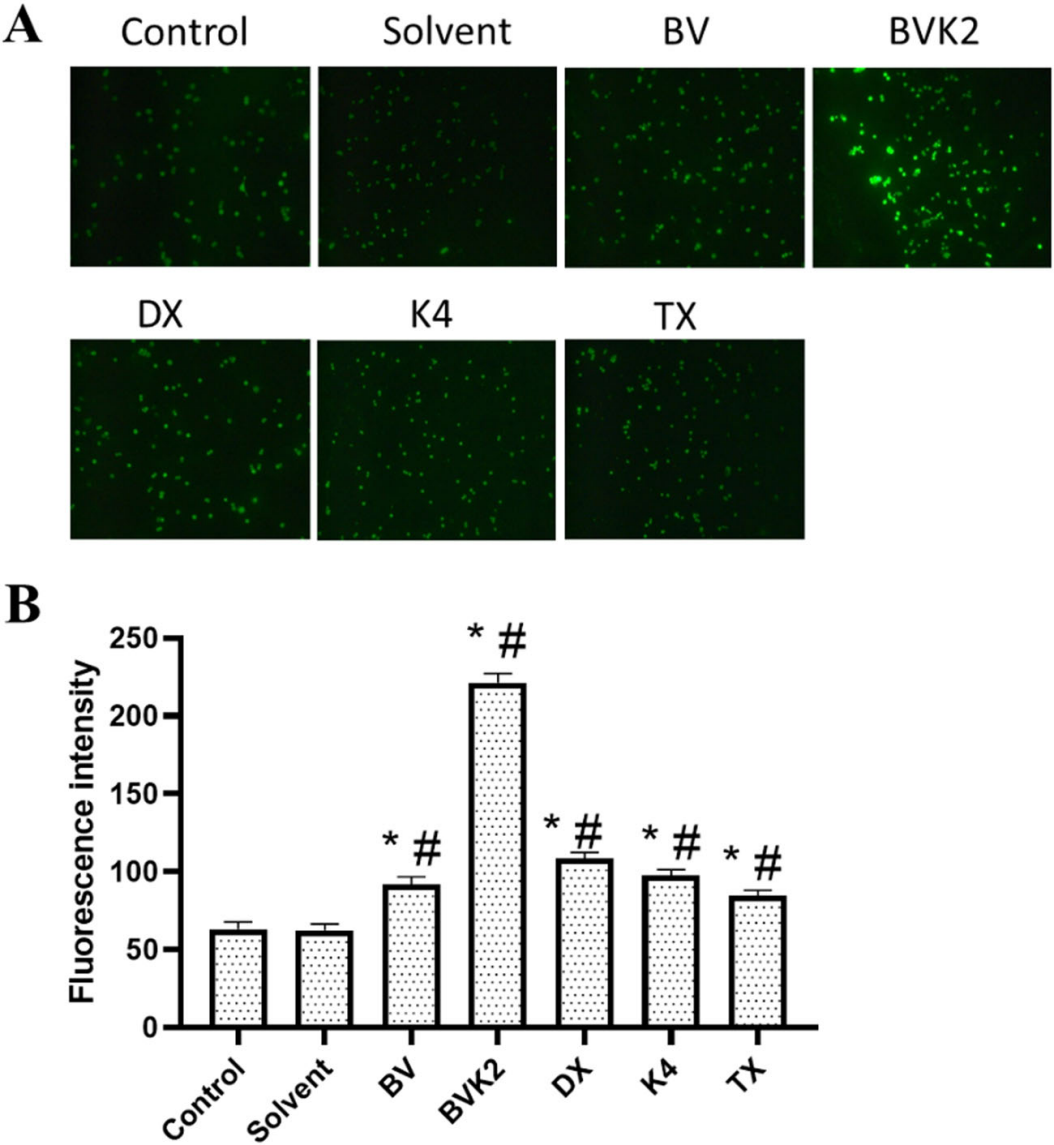
ROS level in A2780 cells after application of test compounds. A Intracellular ROS level in A2780 cells was determined by DCFDA staining (100X). B Fluorescence intensity of ROS levels summarized by bar graph. Values are given as mean ± SEM. *p < 0.05 vs control group; ^#^ *p* < 0.05 vs solvent group.

**Figure 12:**
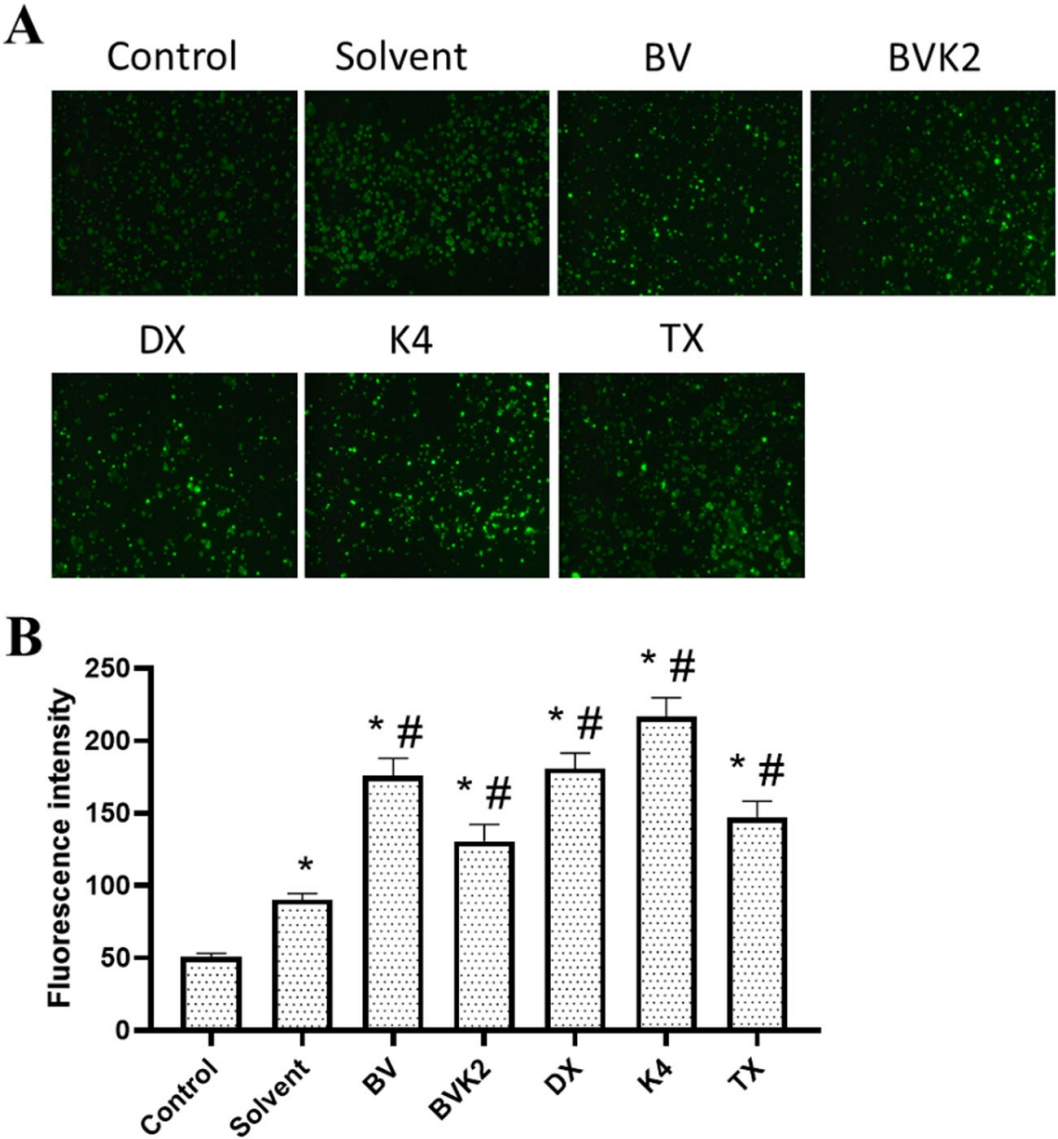
ROS level in Caco2 cells after application of test compounds. A Intracellular ROS level in Caco2 cells was determined by DCFDA staining (100X). B Fluorescence intensity of ROS levels summarized by bar graph. Values are given as mean ± SEM. *p < 0.05 vs control group; ^#^ *p* < 0.05 vs solvent group.

Cells continuously produce ROS during aerobic metabolism. Increased intracellular ROS levels can lead to oxidative damage of cell components, disruption of cellular hemostasis and subsequent cell death. In our study, the effects of the synthesized compounds on the level of reactive oxygen species in cancer and healthy cell lines were determined. Accordingly, solvent application did not cause a significant change in ROS level except for Caco‐2 cells. Among the applied compounds for A2780 cells, only BVK2 displayed significant increases in intracellular ROS levels compared to the control and solvent group (*p* < 0.05) (Figure 11). The applied compounds resulted in significant increases in intracellular ROS levels compared to the control and solvent group (*p* < 0.05) (Figure 12).

## 3 Conclusion

In this study, the cytotoxic effects of novel amino acid‐chalcone conjugated cyclotriphosphazene derivatives in different cancer cell lines and their effects on DNA damage, apoptosis and reactive oxygen species (ROS) were studied. In A2780 and Caco‐2 cell lines, compounds such as BV, BVK1, BVK2, BVK4, K4 significantly decreased cell viability at high doses. Standard chemotherapeutic agents DX and TX similarly decreased cell viability, but their effects varied according to the compounds. When DNA damage parameters were examined, it was found that the compounds caused significant increases in DNA damage indicators such as tail length, tail density and tail moment in A2780 cells, whereas head diameter and density decreased. Similarly, it was observed that these compounds increased DNA damage in Caco‐2 cells. The findings regarding apoptotic cell death revealed that BVK2 and K4 compounds increased the apoptosis level in A2780 and Caco‐2 cells. In addition, it was determined that the applied compounds caused significant increases in ROS levels. The results indicate that these newly synthesized compounds show significant cytotoxic and genotoxic effects in cancer cell lines and these effects may vary depending on the cell type. The obtained findings indicate that the newly synthesized compounds may be potential anticancer drug candidates in line with the pronounced cytotoxic, genotoxic and apoptotic effects of the newly synthesized compounds in cancer cell lines.

## Author Contributions

**Yunus Yücel:** methodology, investigation. **Ferhan Sultan Şeker:** methodology, investigation. **Büşra Aksoy Erden:** investigation, validation, software. **Çiğdem Tekin:** formal analysis, software. **Mücahit Özdemir:** validation, software. **Eray Çalışkan:** resources, methodology, visualization. **Suat Tekin:** methodology, investigation, validation. **Kenan Koran:** writing – review and editing, data curation, conceptualization. **Fatih Biryan:** project administration, funding acquisition, conceptualization.

## Conflicts of Interest

The authors declare no conflicts of interest.

## Supporting information

Supporting information.

## Data Availability

The data that support the findings of this study are available from the corresponding author upon reasonable request. The data is available upon request from corresponding author.
