# Supplementary material for: The First Chalcone Derivatives of Valine‐Based Spiro‐Cyclotriphosphazenes: In Vitro Cytotoxic Properties, Molecular Docking and DNA Damage Mechanism Studies
Source: J Biochem Mol Toxicol. 2025 Mar 24;39(4):e70233. doi: 10.1002/jbt.70233 (PMC11932576; doi:10.1002/jbt.70233)
Supplement: Supplementary file 1 — Supporting information. [file JBT-39-e70233-s001.pdf]

Supporting Information

for

The First Chalcone Derivatives of Valine-Based Spiro-cyclotriphosphazenes: In vitro Cytotoxic Properties, Molecular Docking and DNA Damage Mechanism Studies

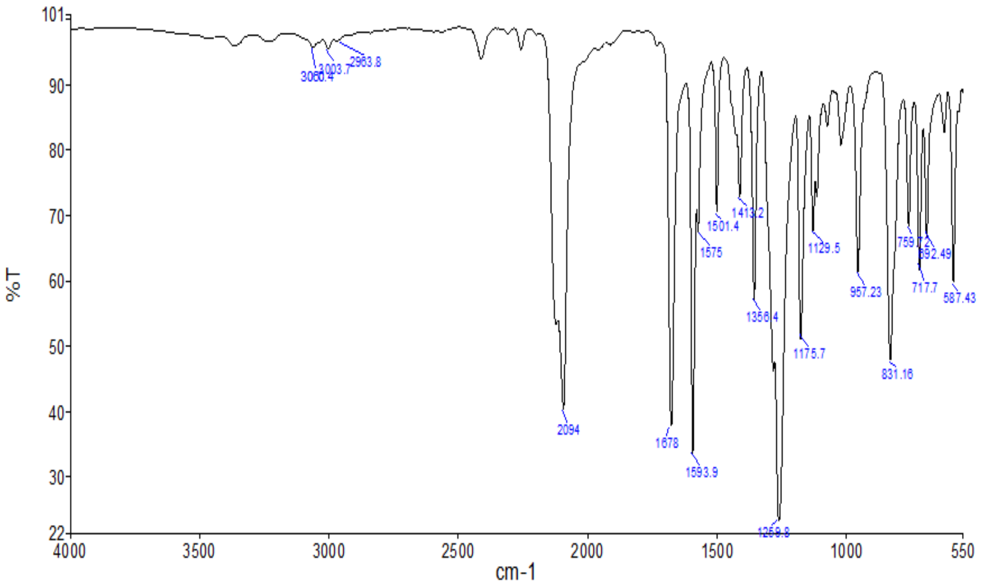

Figure S1. FT-IR (ATR) spectra of **N_3-AF**

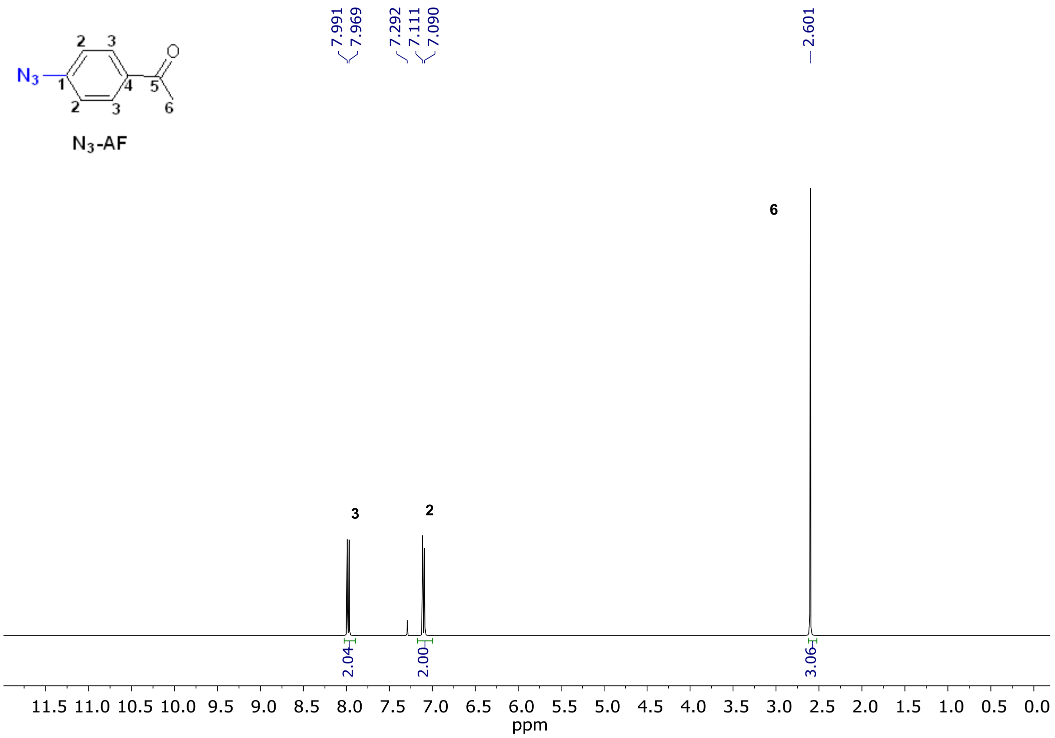

Figure S2. ^{1}H-NMR spectra of **N_3-AF** (DMSO-d_6)

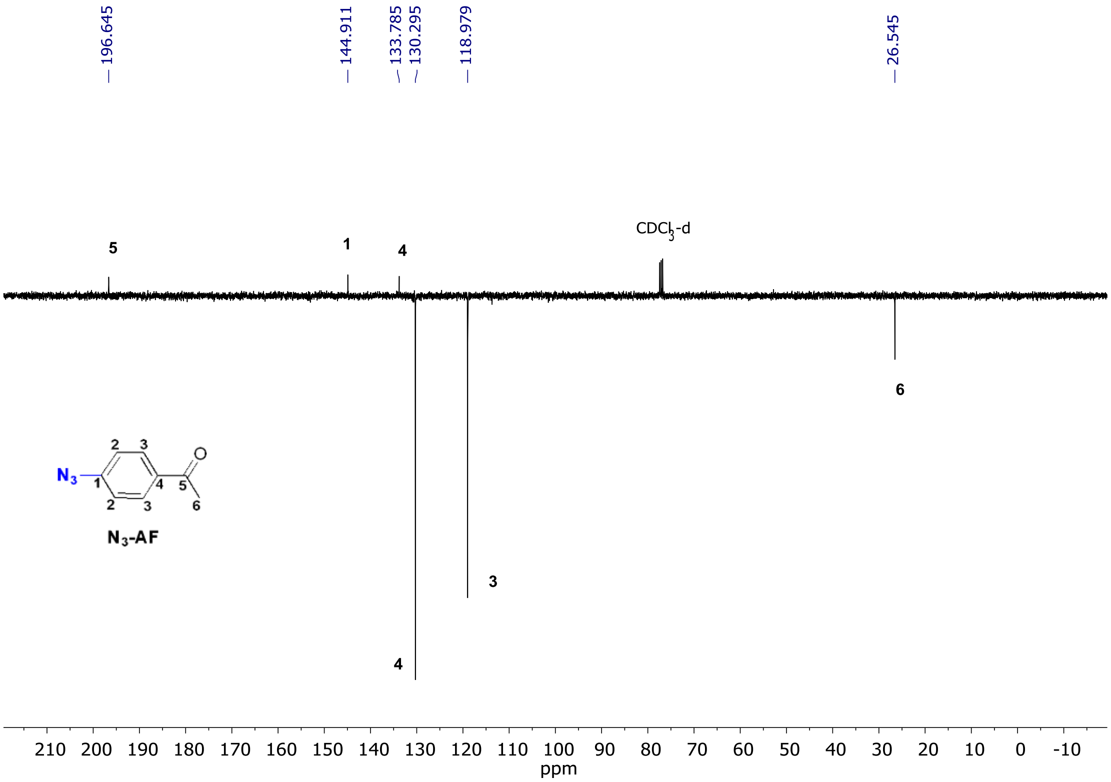

Figure S3. ^{13}C-NMR spectra of **N_3-AF** (DMSO-d_6)

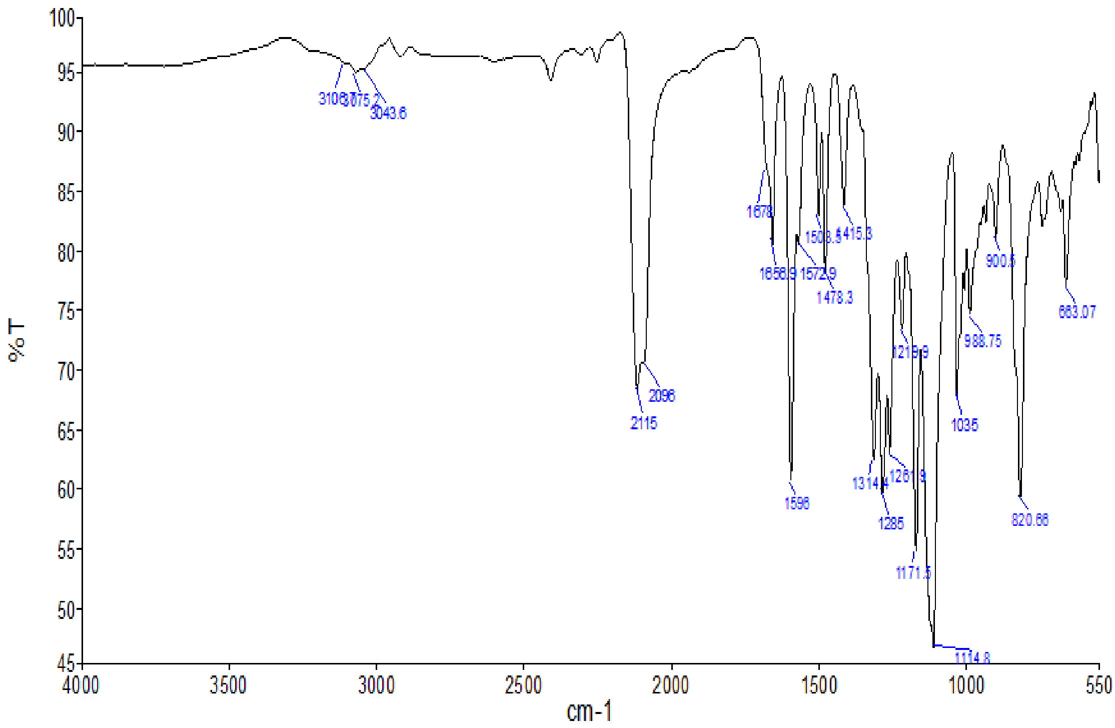

Figure S4. FT-IR (ATR) spectra of **4-Cl-3-CF_3-chalcone-N_3 (K1)**

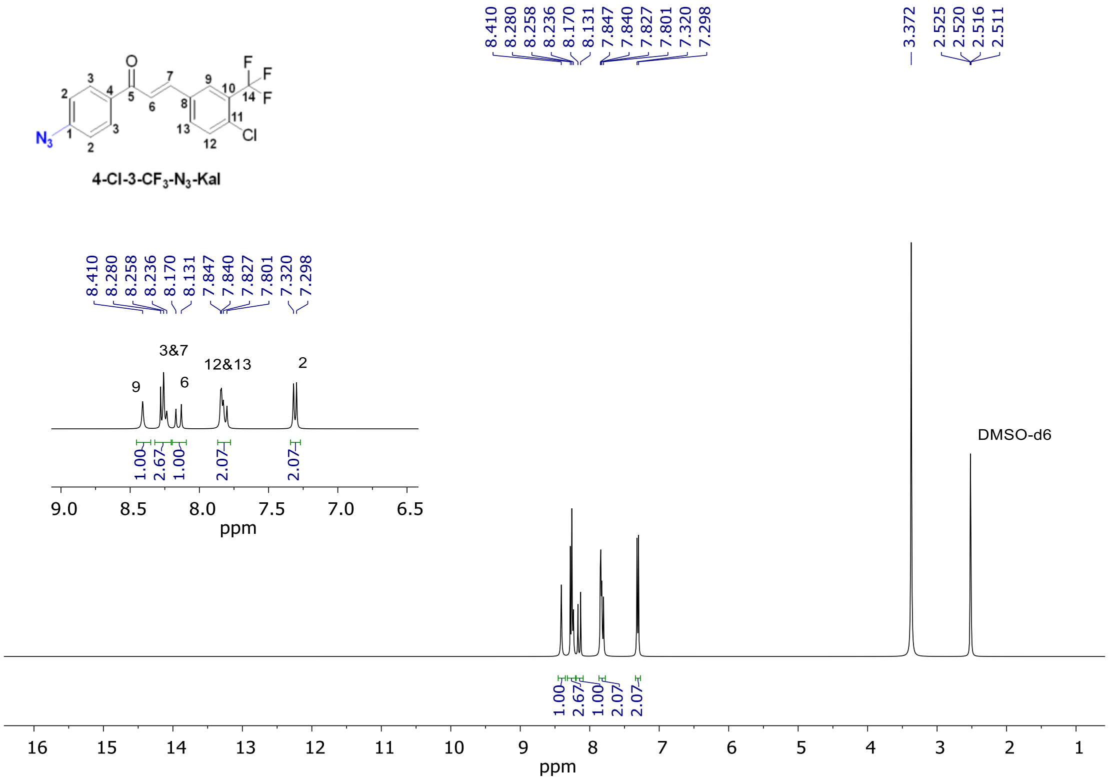

Figure S5. ^{1}H-NMR spectra of **4-Cl-3-CF$_3$-chalcone-N$_3$** (DMSO-d$_6$) (**K1**)

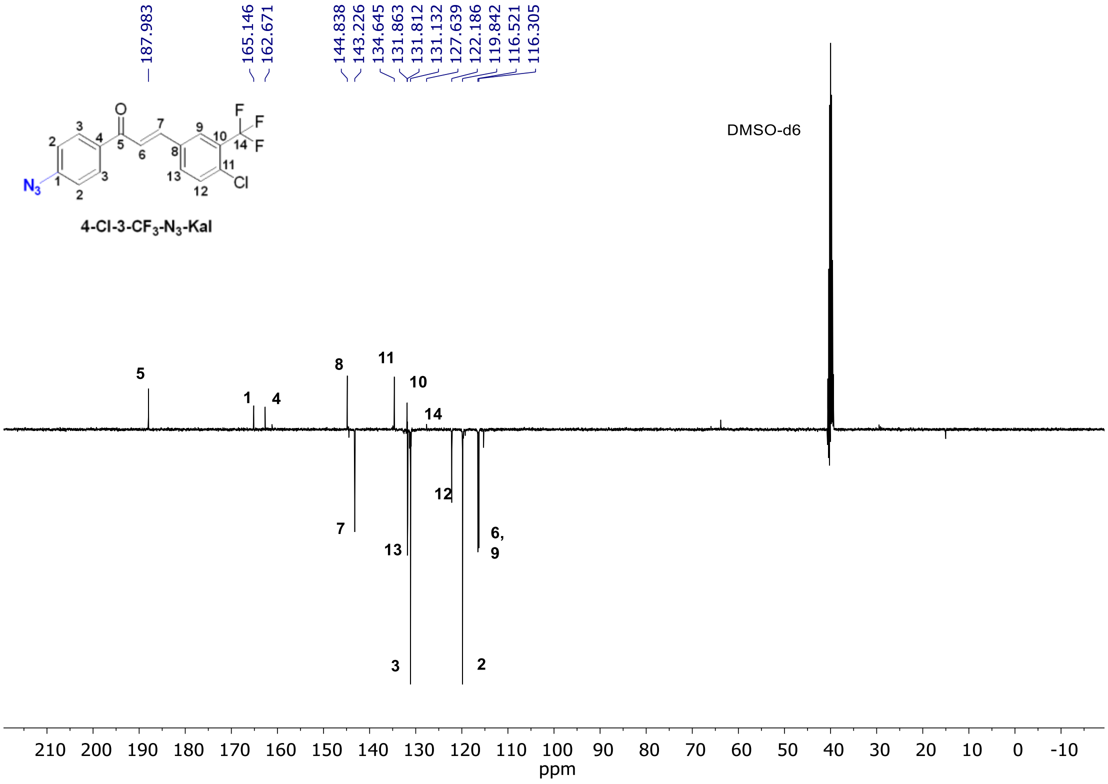

Figure S5. ^{13}C-APT NMR spectra of **4-Cl-3-CF$_3$-chalcone-N$_3$** (DMSO-d$_6$) (**K1**)

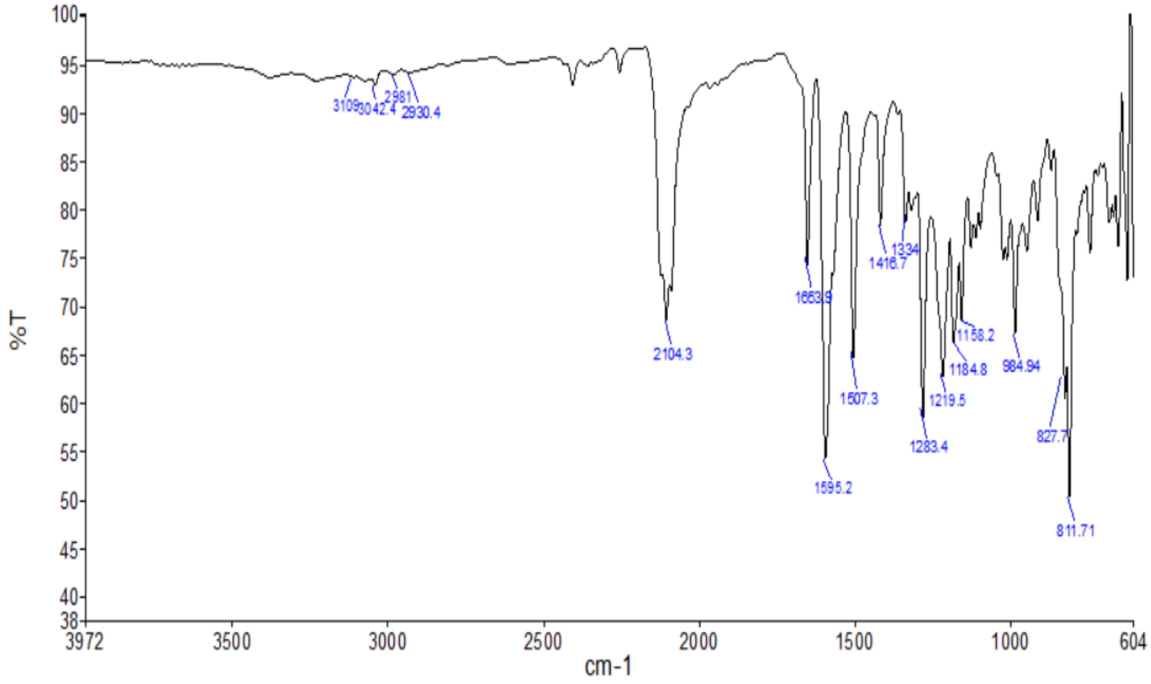

Figure S6. FT-IR (ATR) spectra of **4-F-chalcone-N$_3$-Kal (K2)**

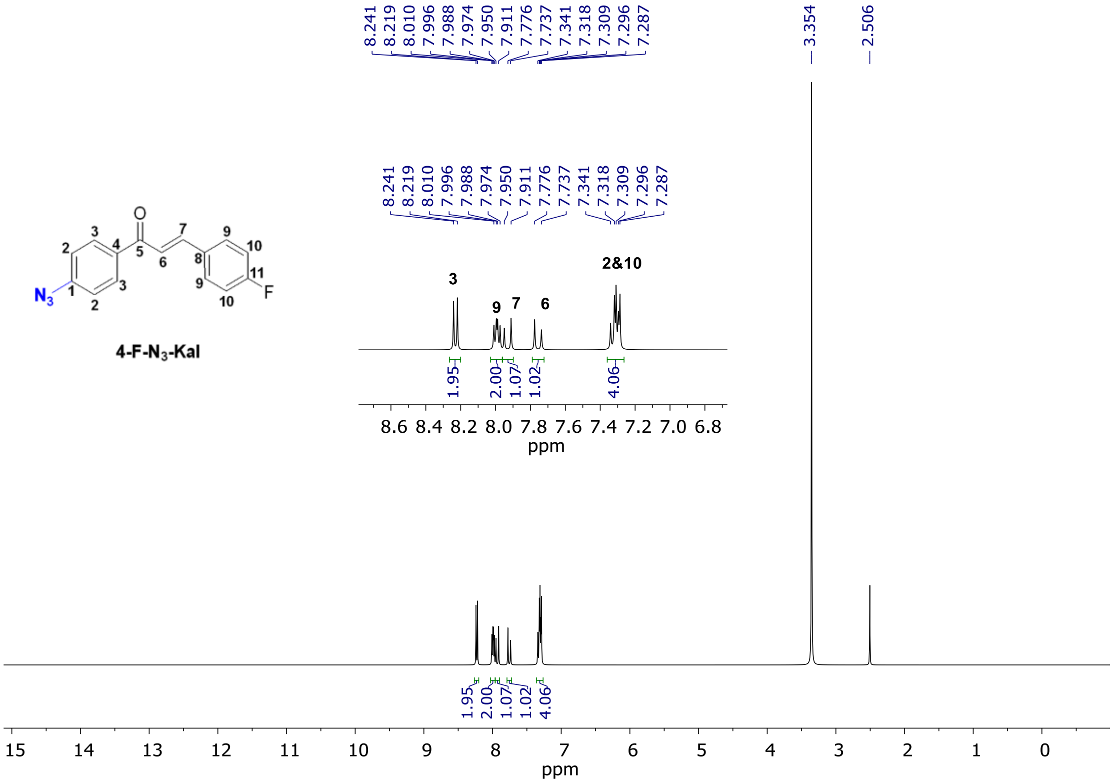

Figure S7. ^{1}H-NMR spectra of **4-F-chalcone-N$_3$** (**K2**) (DMSO-d$_6$)

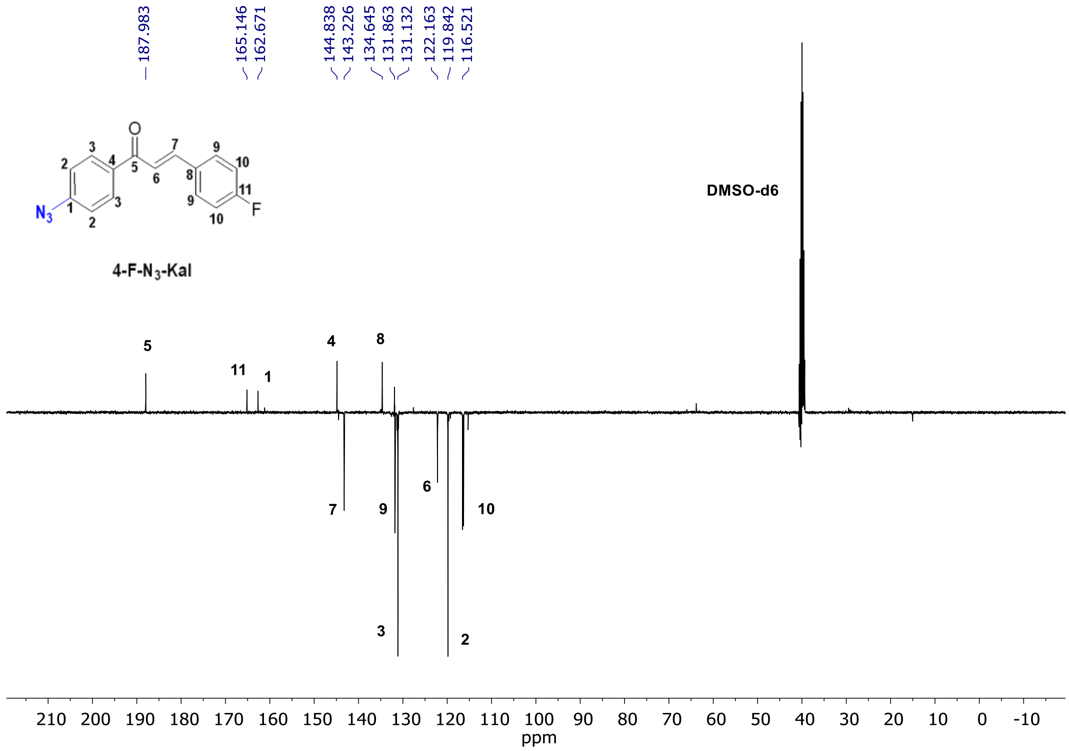

Figure S8. ^{13}C-APT NMR spectra of **4-F-N$_3$-Kal** (DMSO-d$_6$) (**K2**)

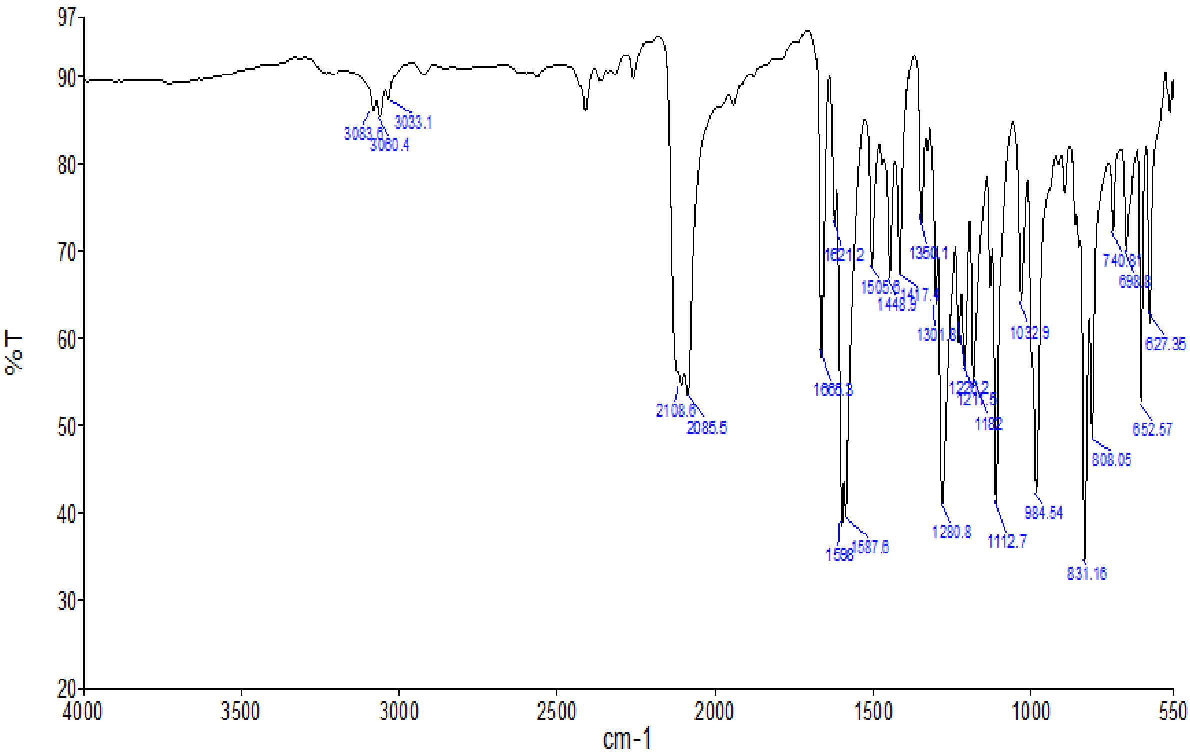

Figure S9. FT-IR (ATR) spectra of **3,5-DiF-chalcone-N$_3$** (**K3**)

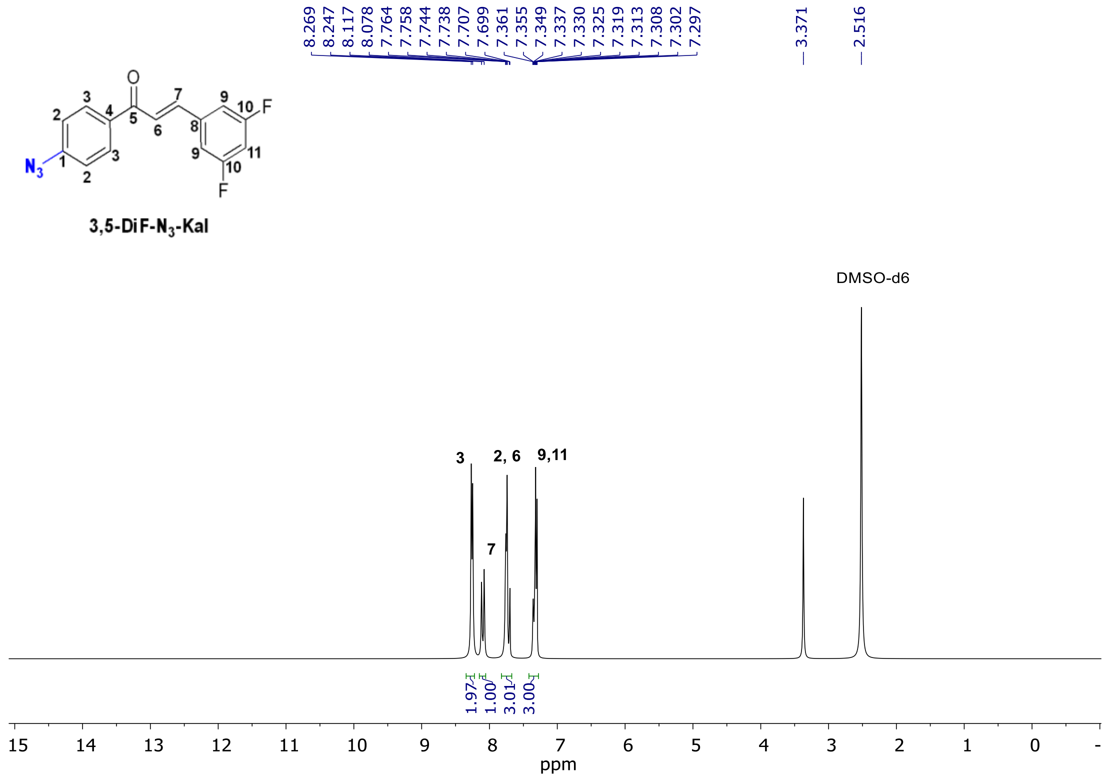

Figure S10. ^{1}H-NMR **spectra of 3,5-DiF-chalcone-N_3 (K3)** (DMSO-d_6)

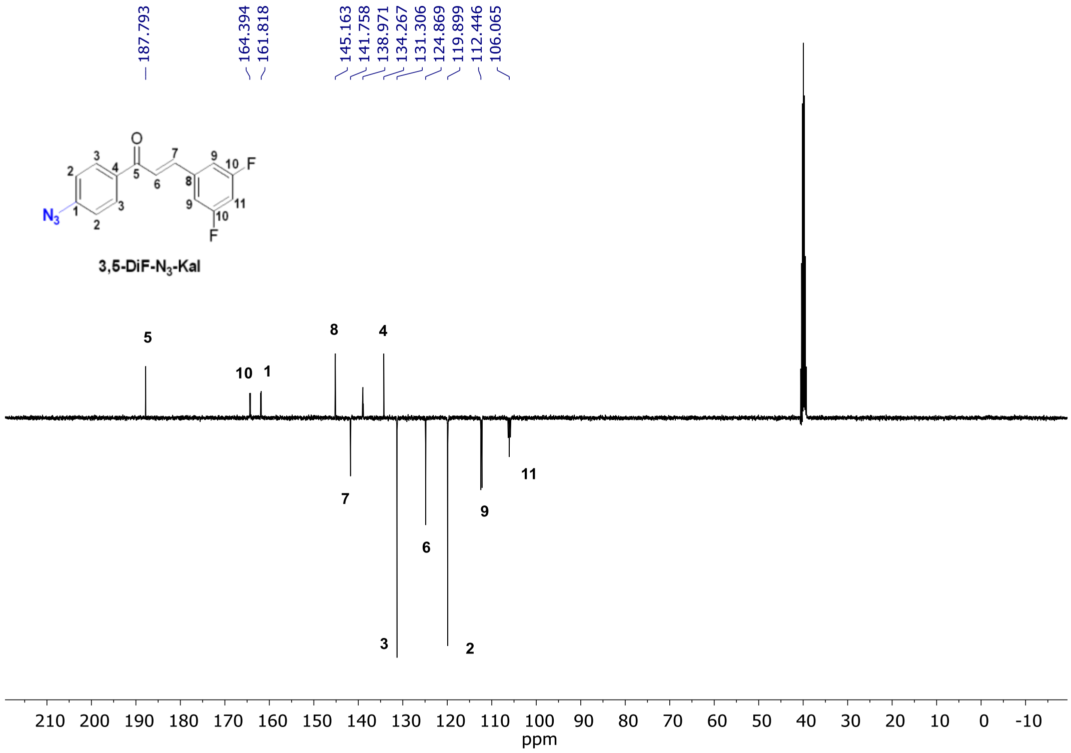

Figure S11. ^{13}C-APT NMR spectra of **3,5-DiF-chalcone-N_3 (K3)** (DMSO-d_6)

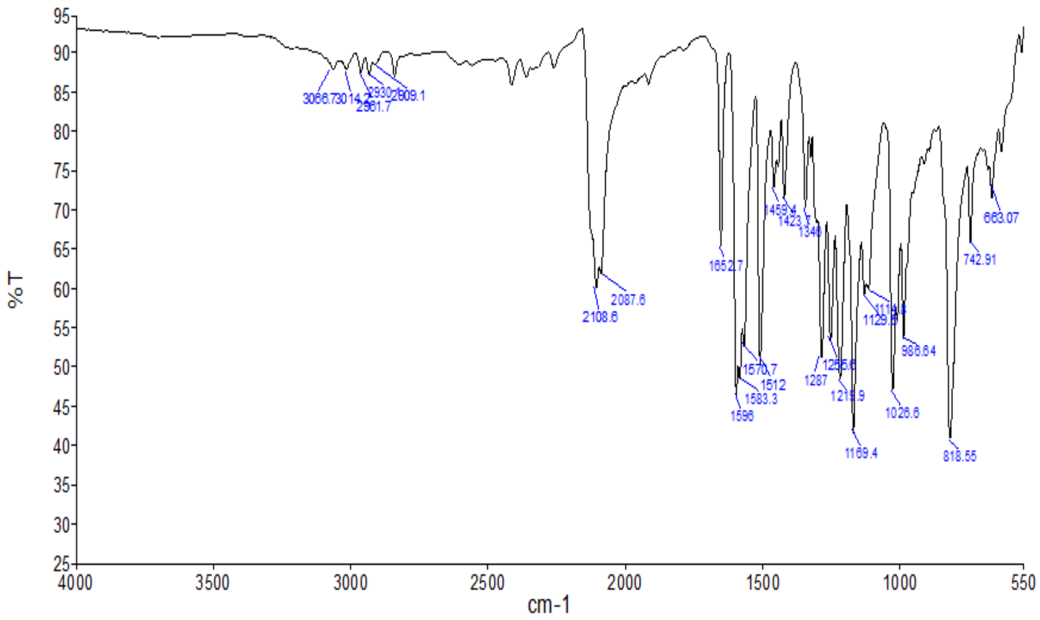

Figure S12. FT-IR (ATR) spectra of ***4-OCH$_3$-chalcone-N$_3$ (K4)***

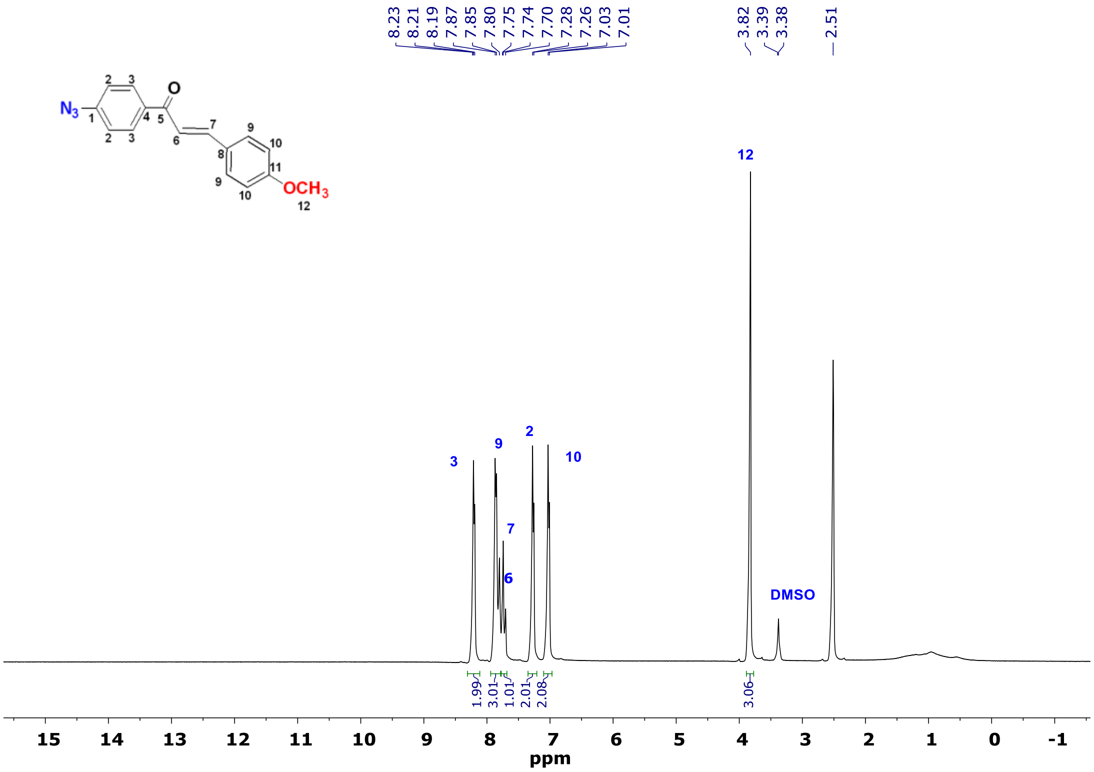

Figure S13. ^{1}H-NMR spectra of ***4-OCH$_3$-chalcone-N$_3$ (K4)*** (DMSO-d$_6$)

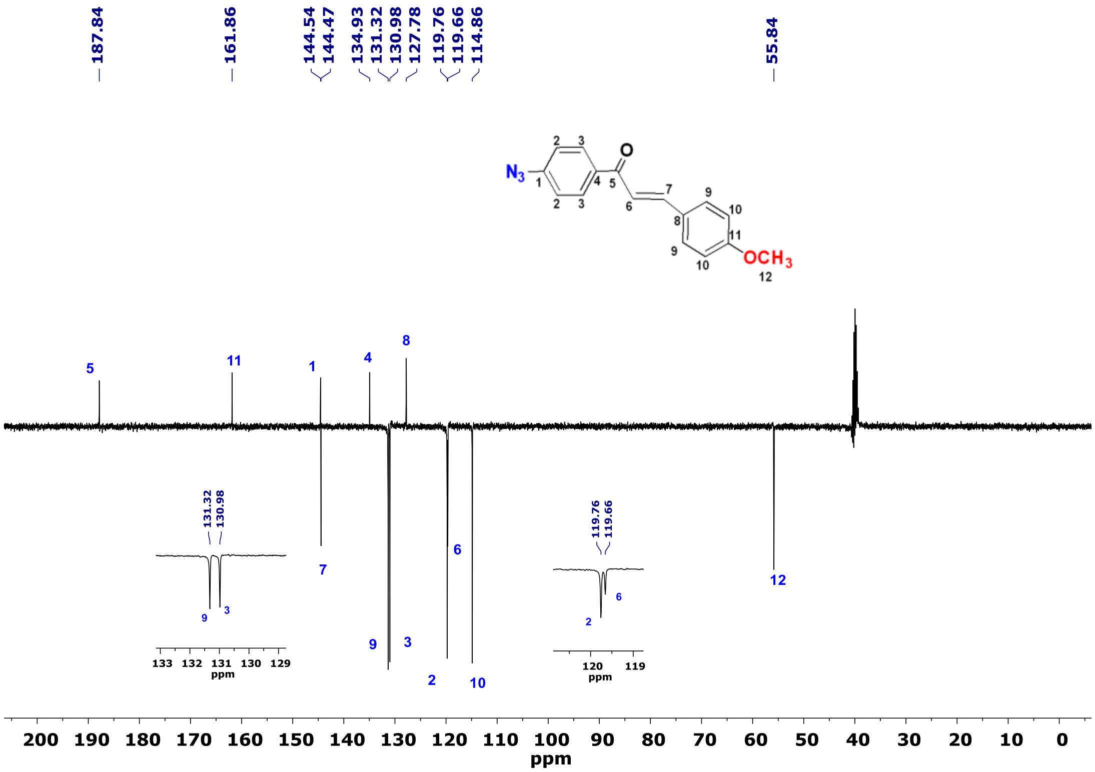

Figure S14. ^{13}C-NMR spectra of ***4-OCH$_3$-chalcone-N$_3$ (K4)*** (DMSO-d$_6$)

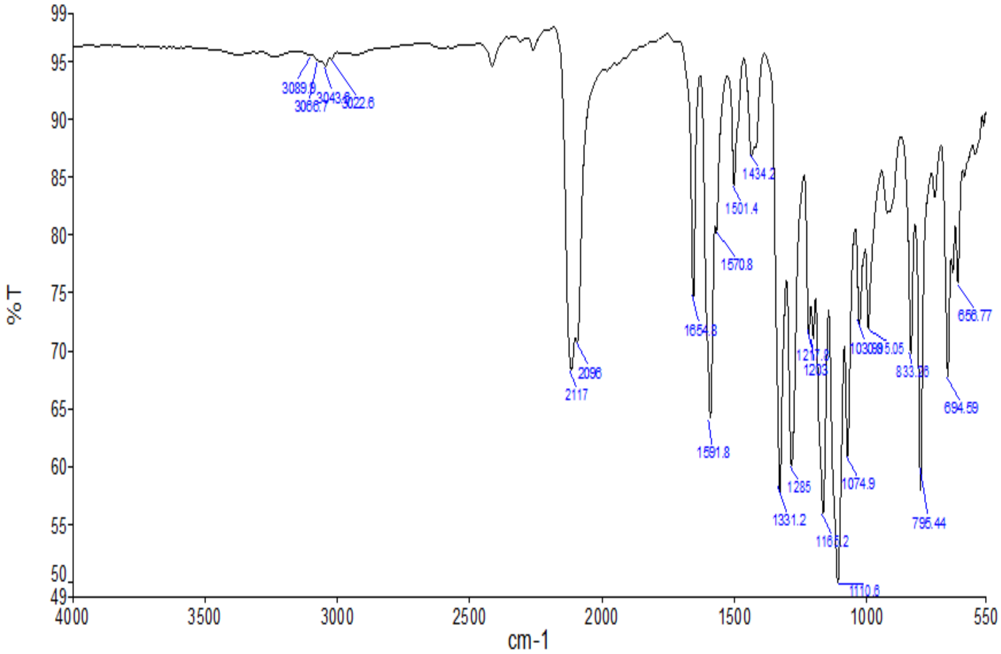

Figure S15. FT-IR (ATR) spectra of **3-CF$_3$*-chalcone-N$_3$ (K5)***

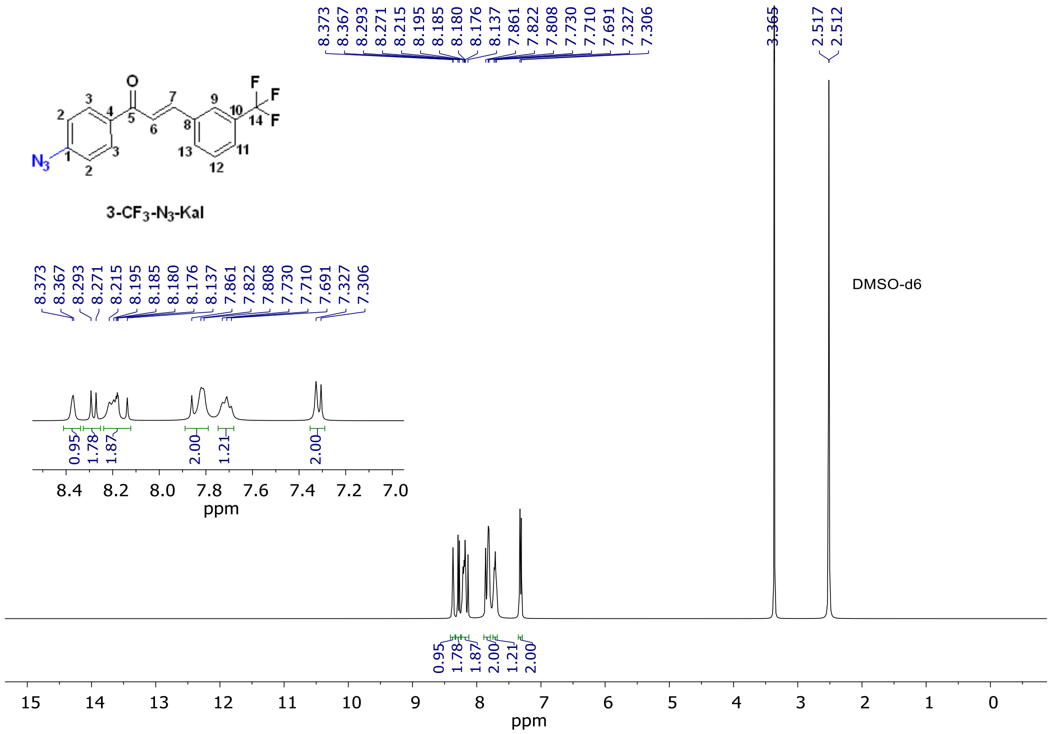

Figure S16. ^{1}H-NMR spectra of **3-CF$_3$-*chalcone-N$_3$ (K5)*** (DMSO-d$_6$)

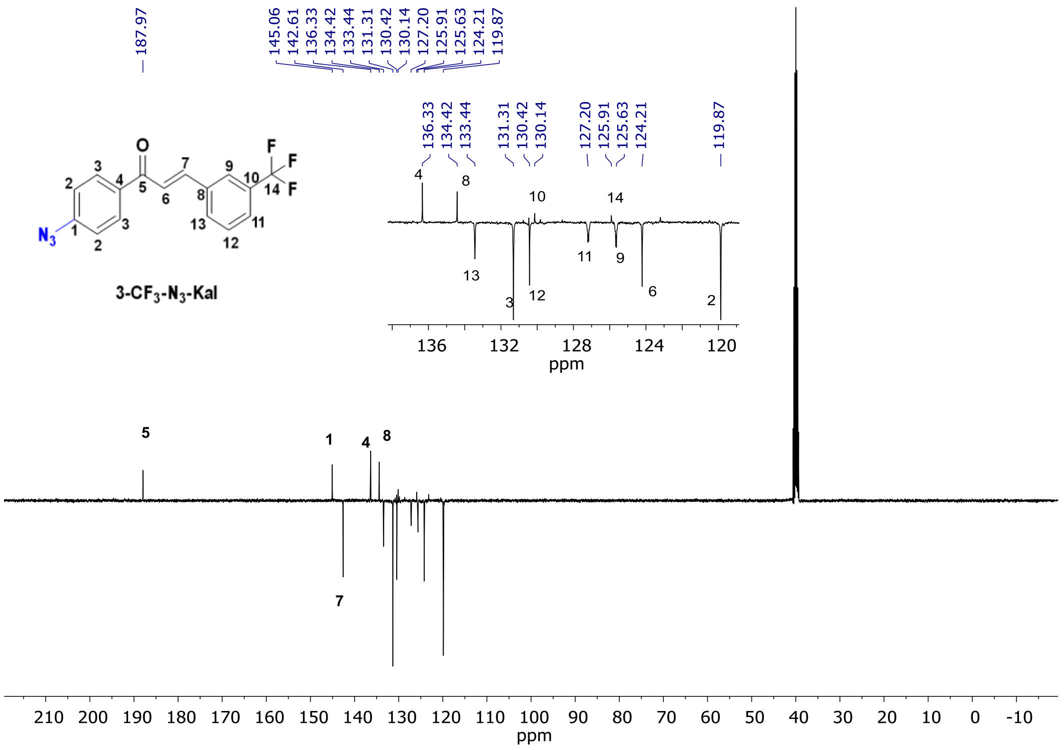

Figure S17. ^{13}C-NMR spectra of **3-CF$_3$-*chalcone-N$_3$ (K5)*** (DMSO-d$_6$)

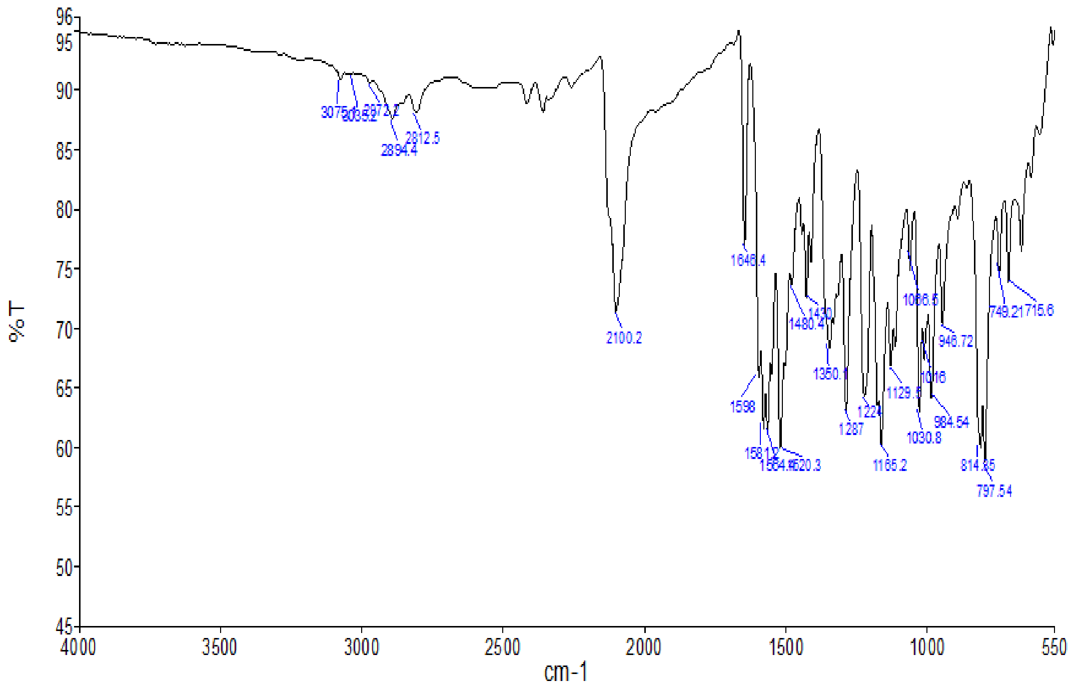

Figure S18. FT-IR (ATR) spectra of ***4-N(CH$_3$)$_2$-chalcone-N$_3$ (K6)***

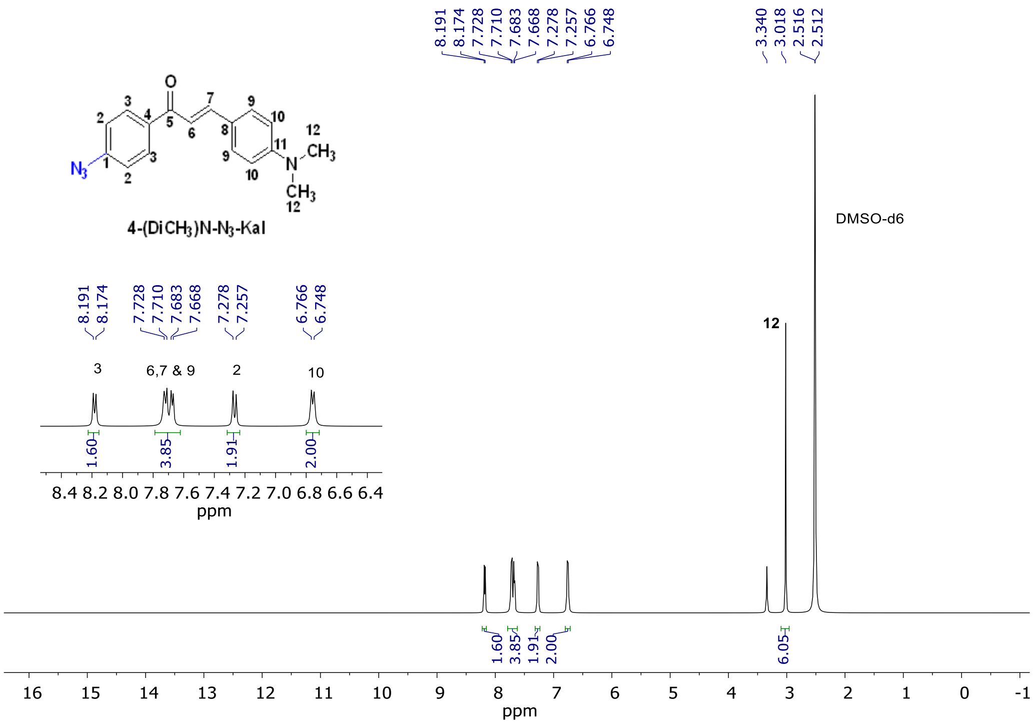

Figure S19. ^{1}H-NMR spectra of ***4-N(CH$_3$)$_2$-chalcone-N$_3$ (K6)*** (DMSO-d$_6$)

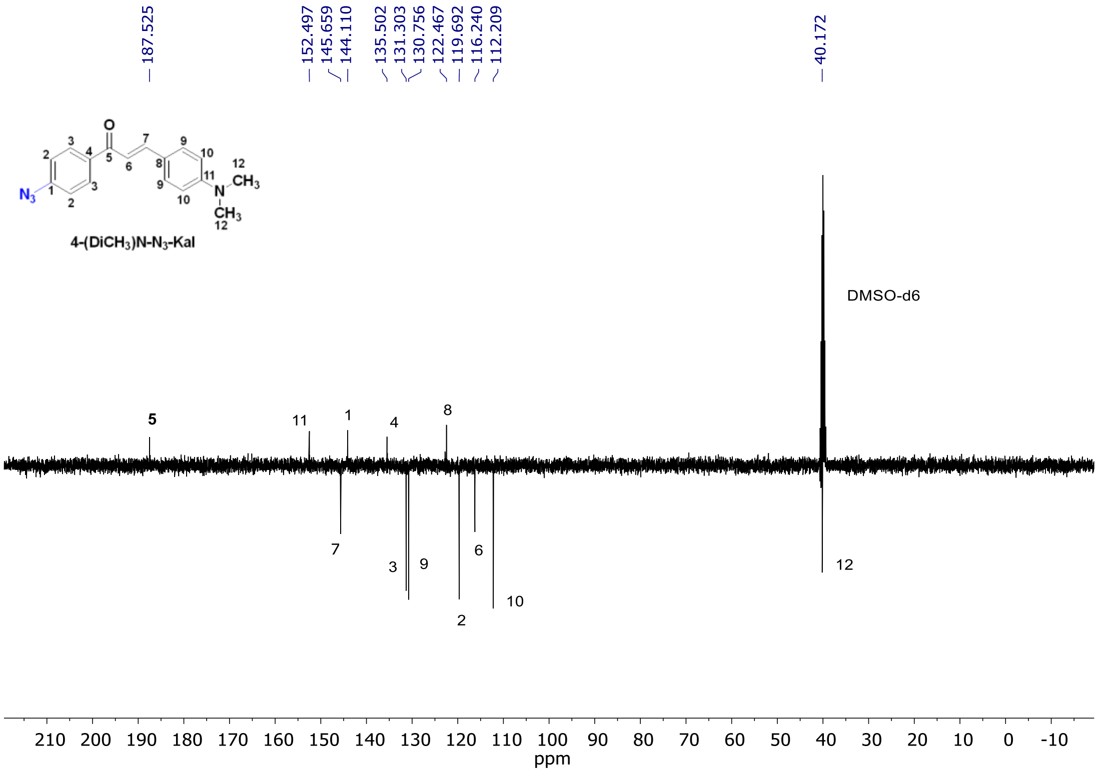

Figure S20. ^{13}C-NMR spectra of ***4-N(CH$_3$)$_2$-chalcone-N$_3$ (K6)*** (DMSO-d$_6$)

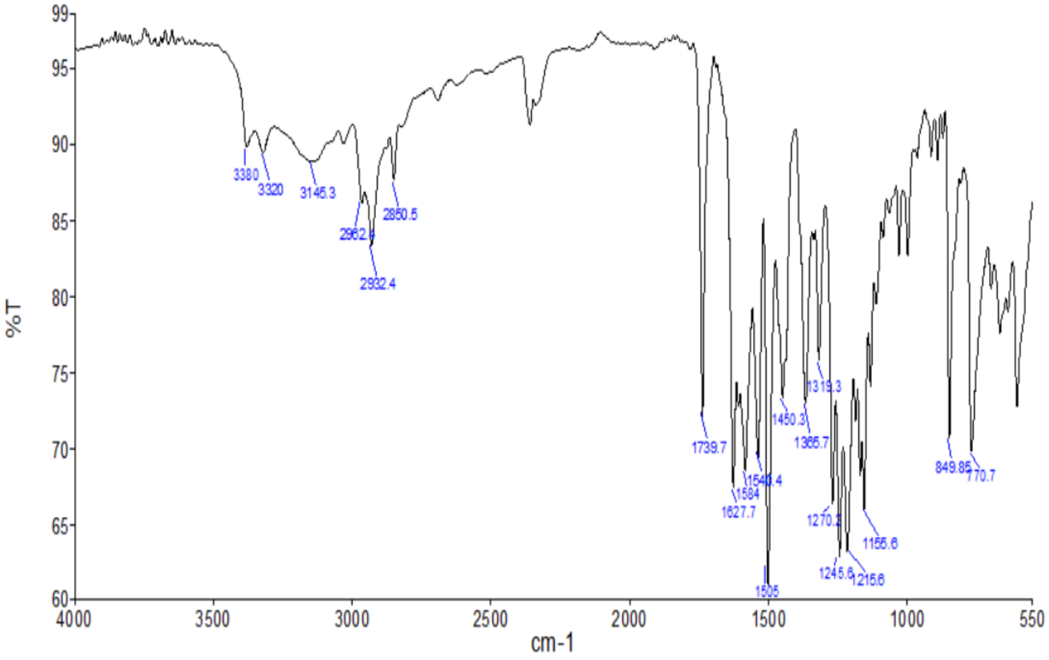

Figure S21. FT-IR (ATR) spectra of **OH-Bnz-Val-OCH$_3$**

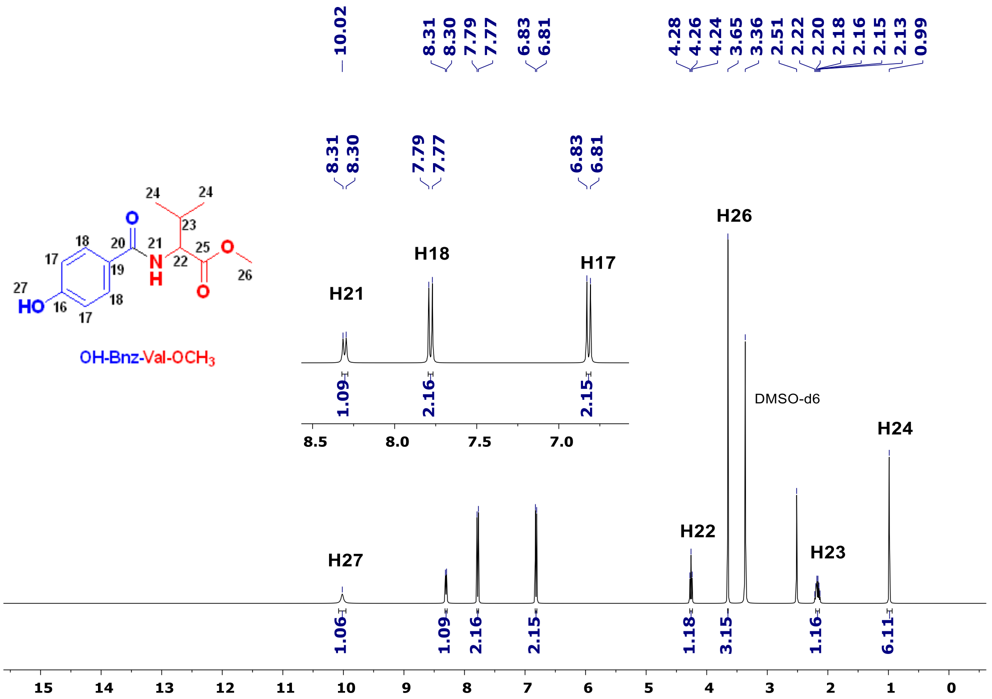

Figure S22. ^{1}H-NMR spectra of **OH-Bnz-Val-OCH$_3$** (DMSO-d$_6$)

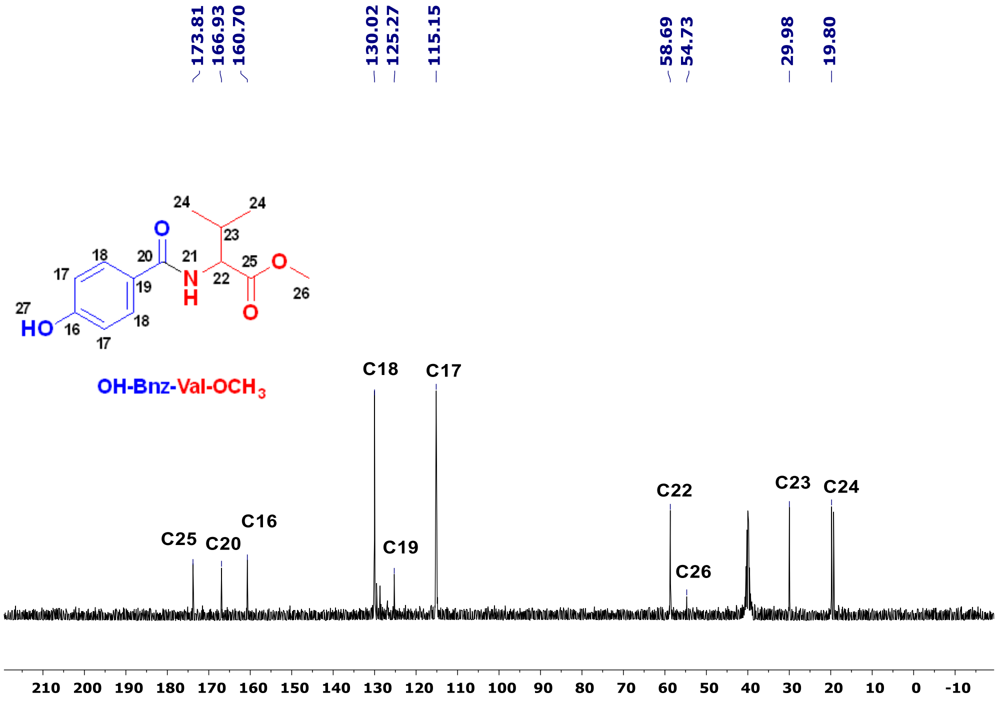

Figure S23. ^{13}C-NMR spectra of **OH-Bnz-Val-OCH$_3$** (DMSO-d$_6$)

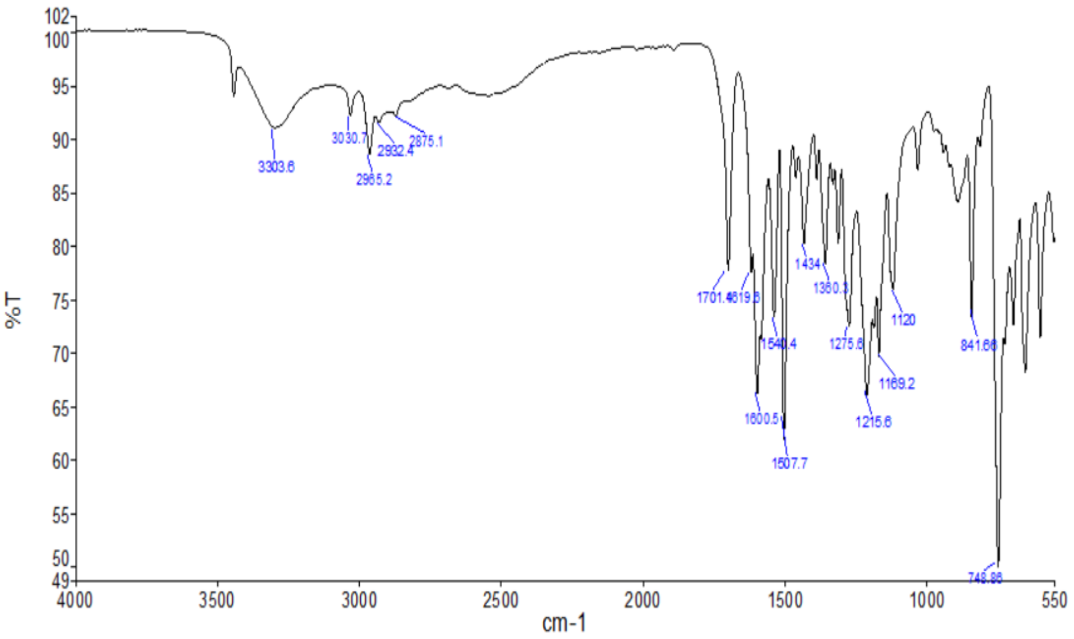

Figure S4. FT-IR (ATR) spectra of **OH-Bnz-Val-OH**

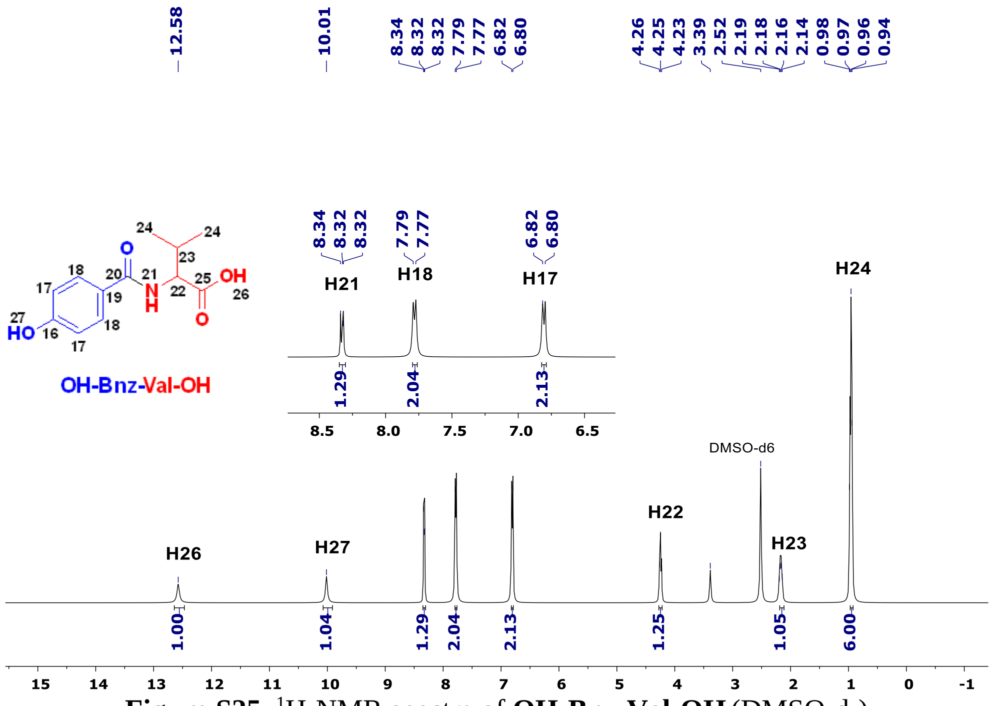

Figure S25. ^{1}H-NMR spectra of **OH-Bnz-Val-OH** (DMSO-d_6)

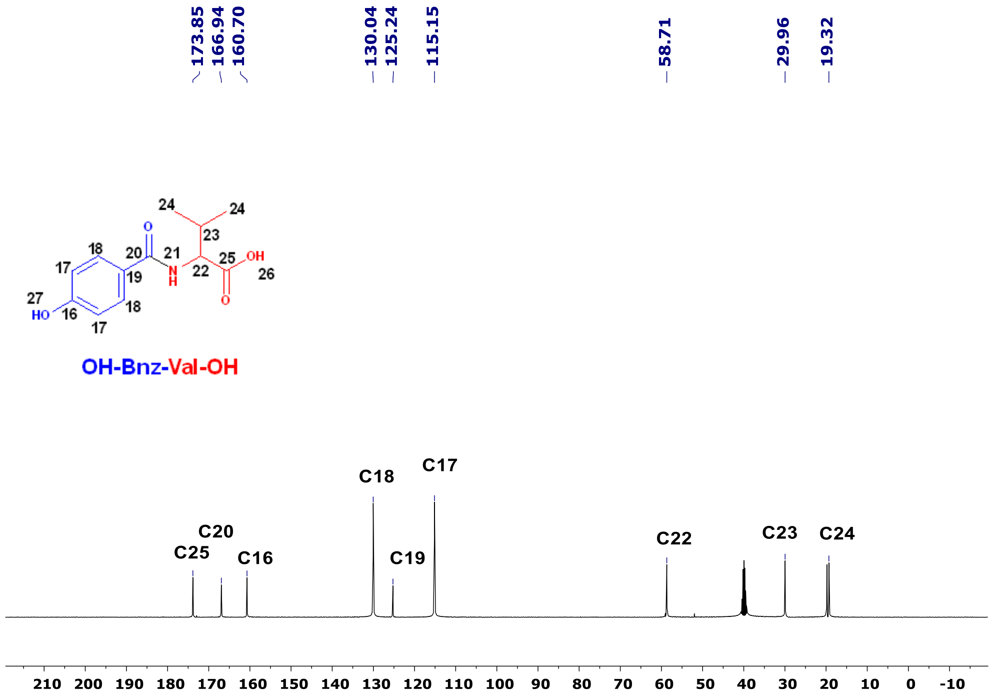

Figure S26. ^{13}C-NMR spectra of **OH-Bnz-Val-OH** (DMSO-d_6)

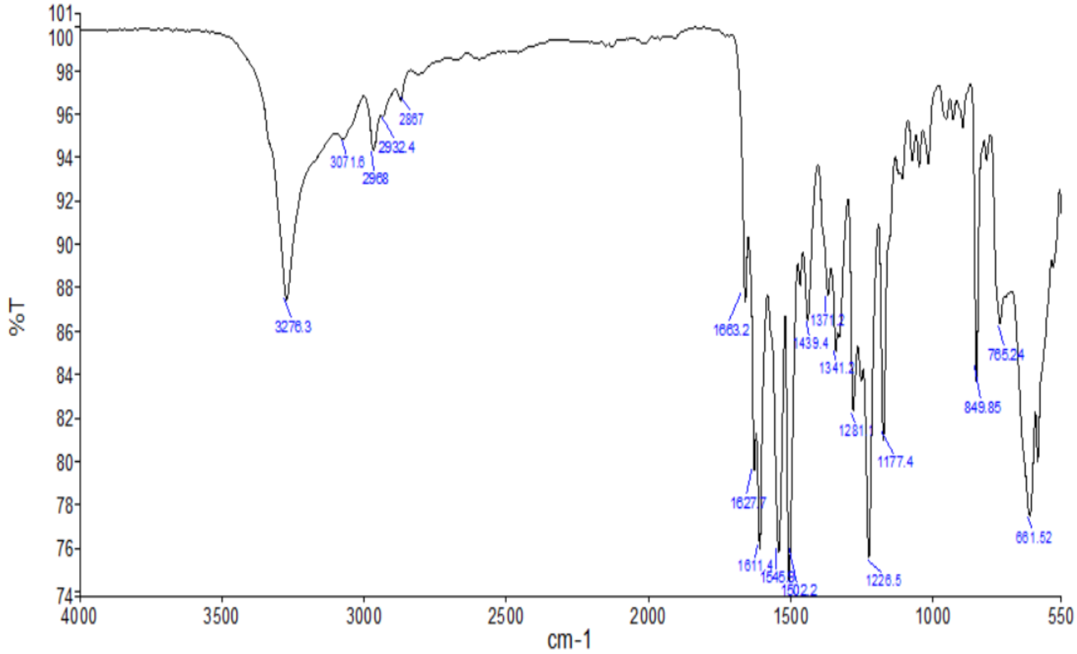

Figure S27. FT-IR (ATR) spectra of **OH-Bnz-Val-PA (VPA)**

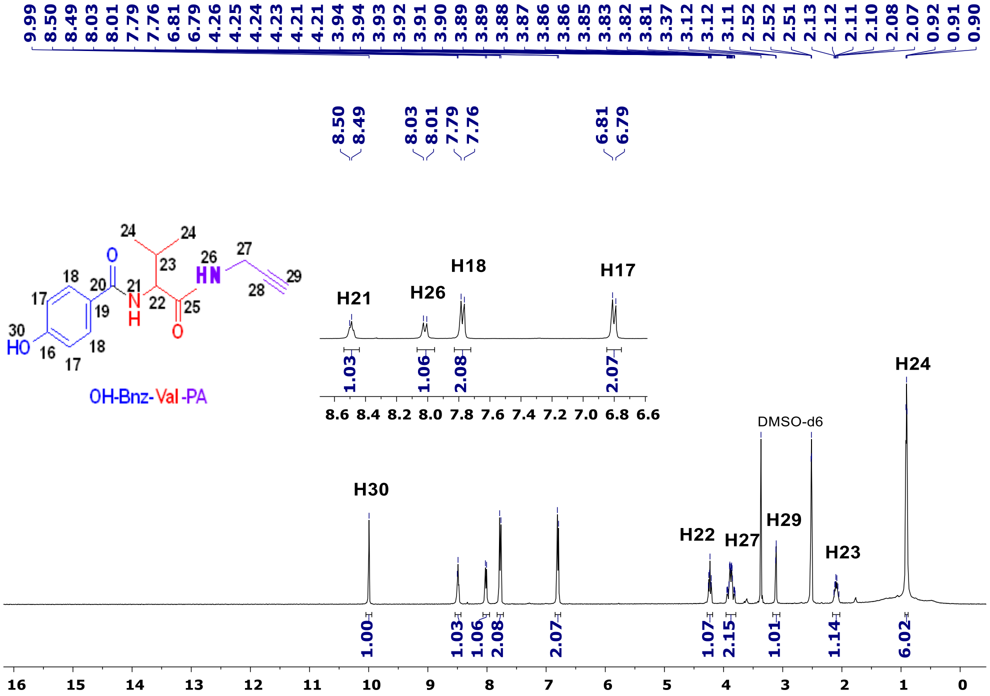

Figure S28. ^{1}H-NMR spectra of **OH-Bnz-Val-PA (VPA)** (DMSO-d_6)

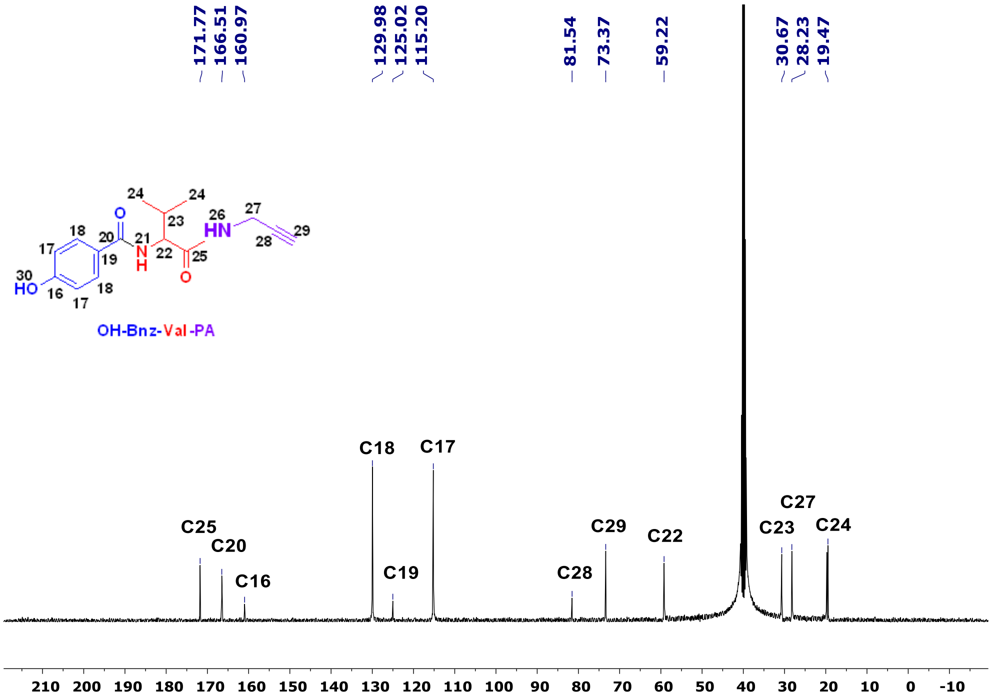

Figure S29. ^{13}C-NMR spectra of **OH-Bnz-Val-PA (VPA)** (DMSO-d_6)

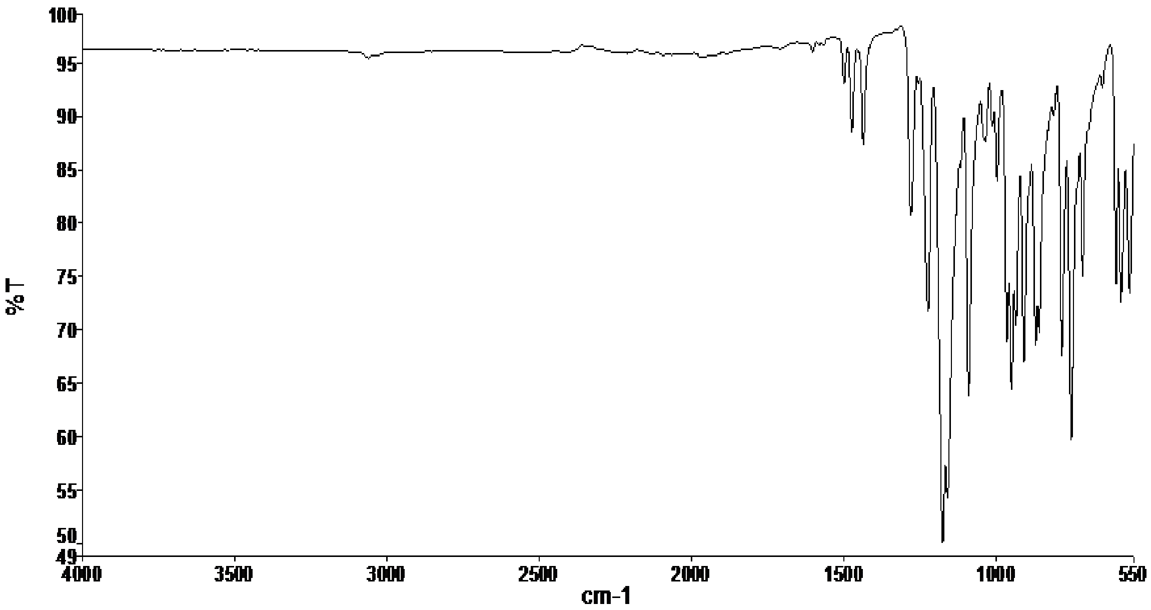

Figure S30. FT-IR (ATR) spectra of **DPP**

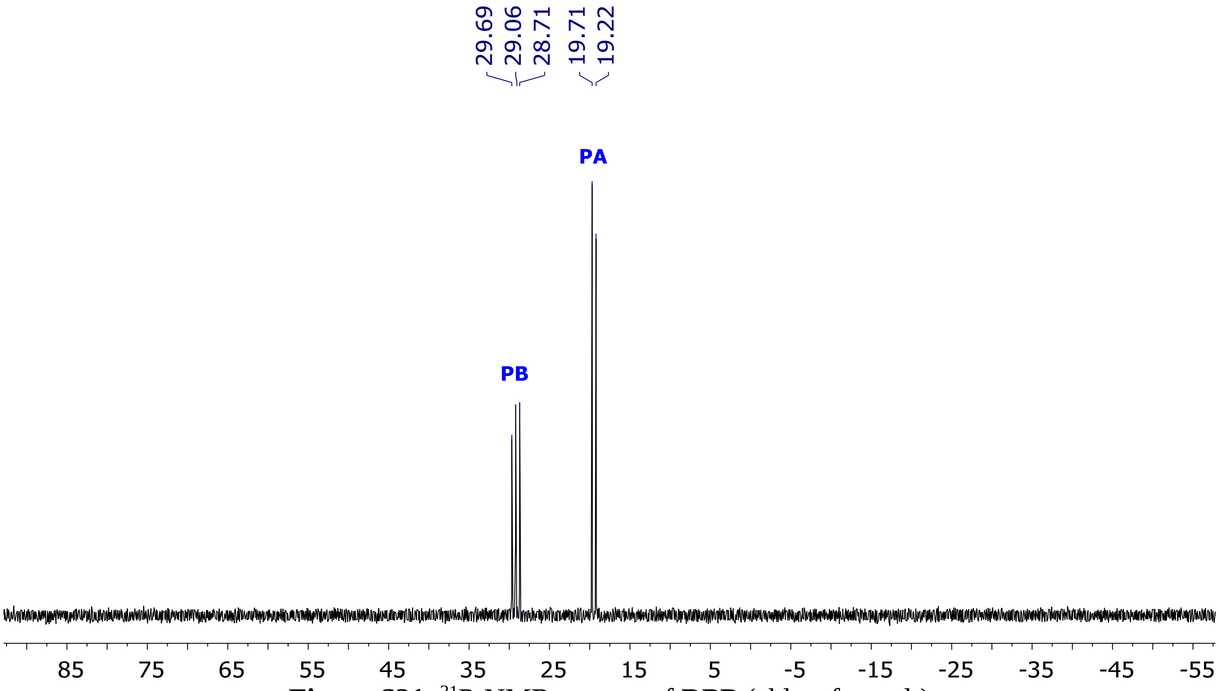

Figure S31. ^{31}P-NMR spectra of **DPP** (chloroform-d_1)

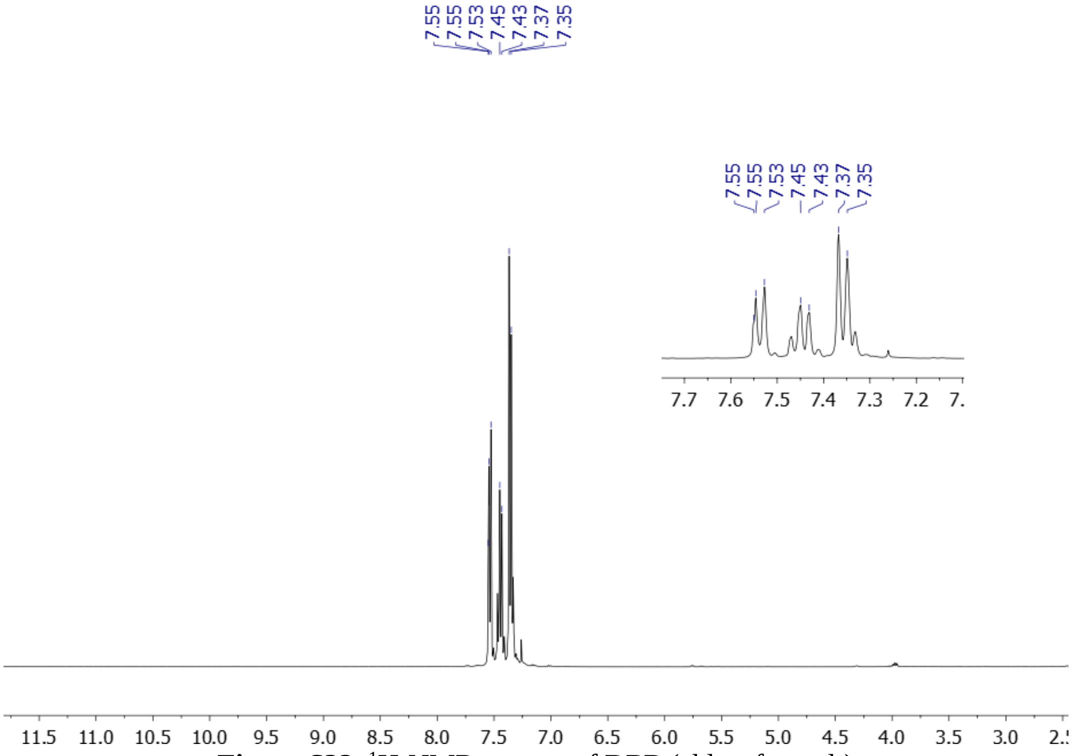

Figure S32. ^{1}H-NMR spectra of **DPP** (chloroform-d_1)

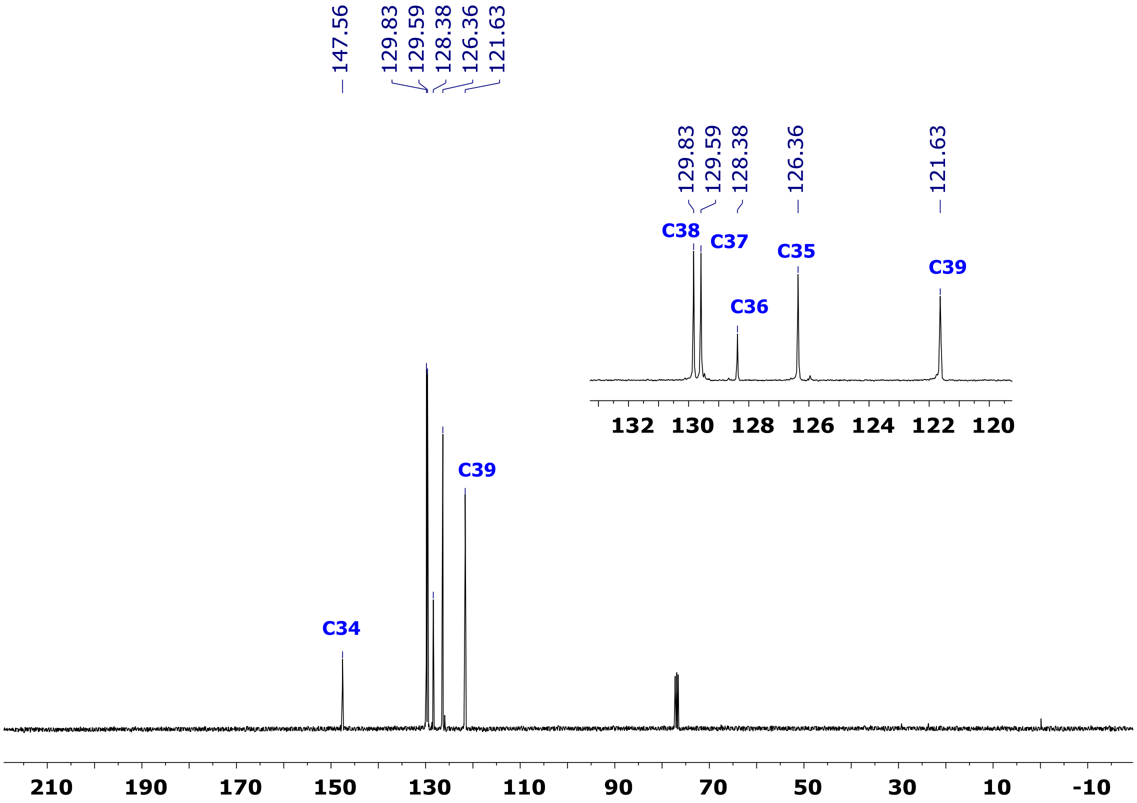

Figure S33. ^{13}C-NMR spectra of **DPP** (chloroform-d_1)

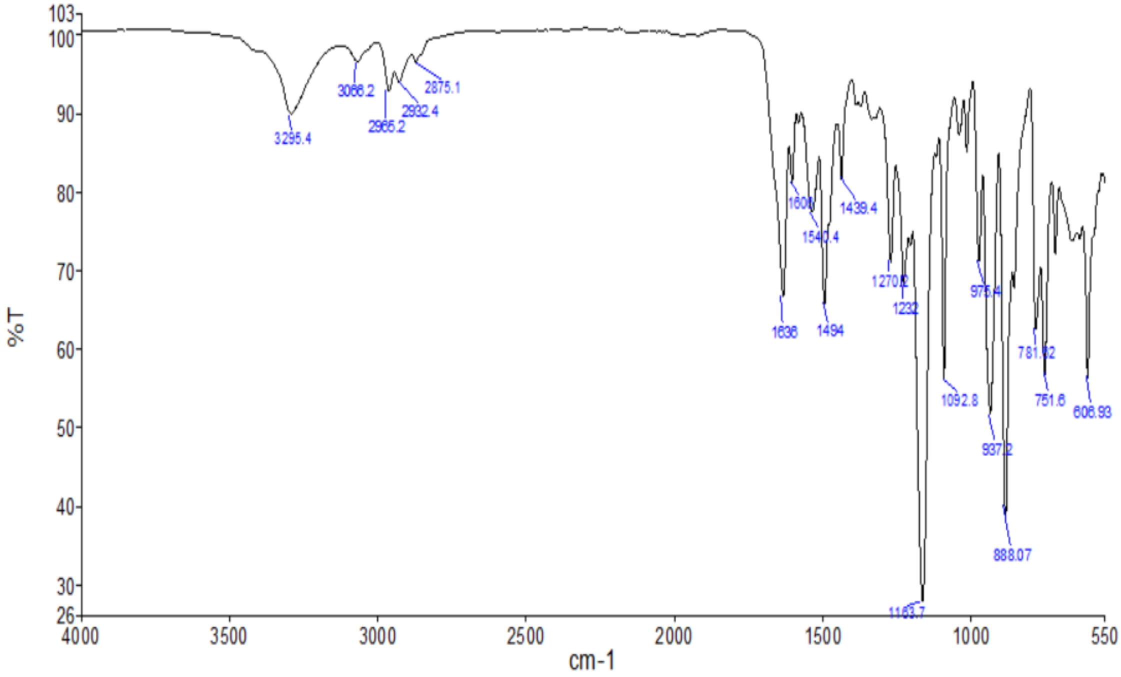

Figure S34. FT-IR (ATR) spectra of **DPP-Bnz-Val-PA (BV)**

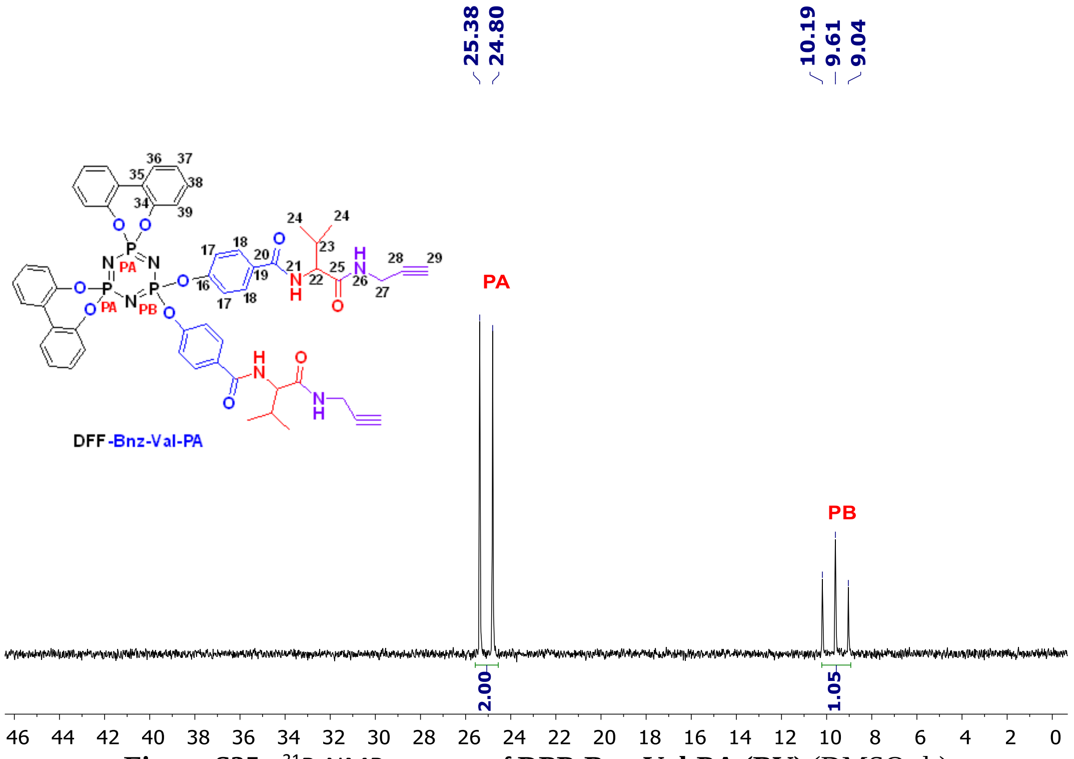

Figure S35. ^{31}P-NMR spectra of **DPP-Bnz-Val-PA (BV)** (DMSO-d_6)

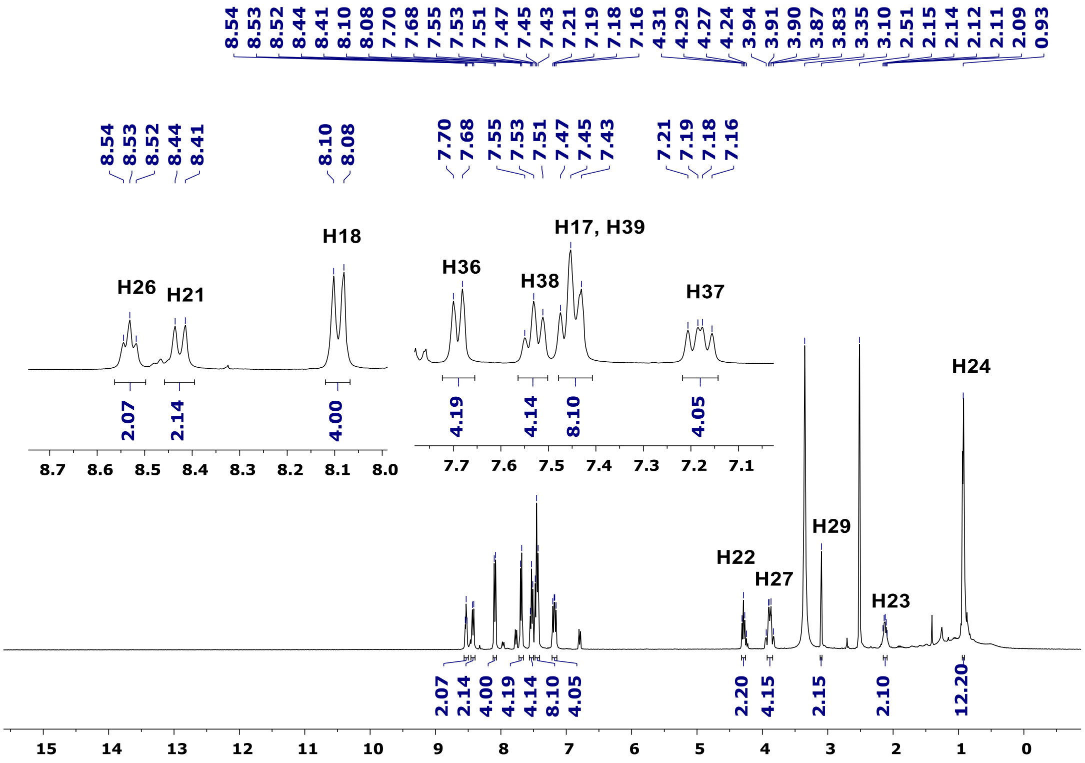

Figure S36. ^{1}H-NMR spectra of **DPP-Bnz-Val-PA (BV)** (DMSO-d_6)

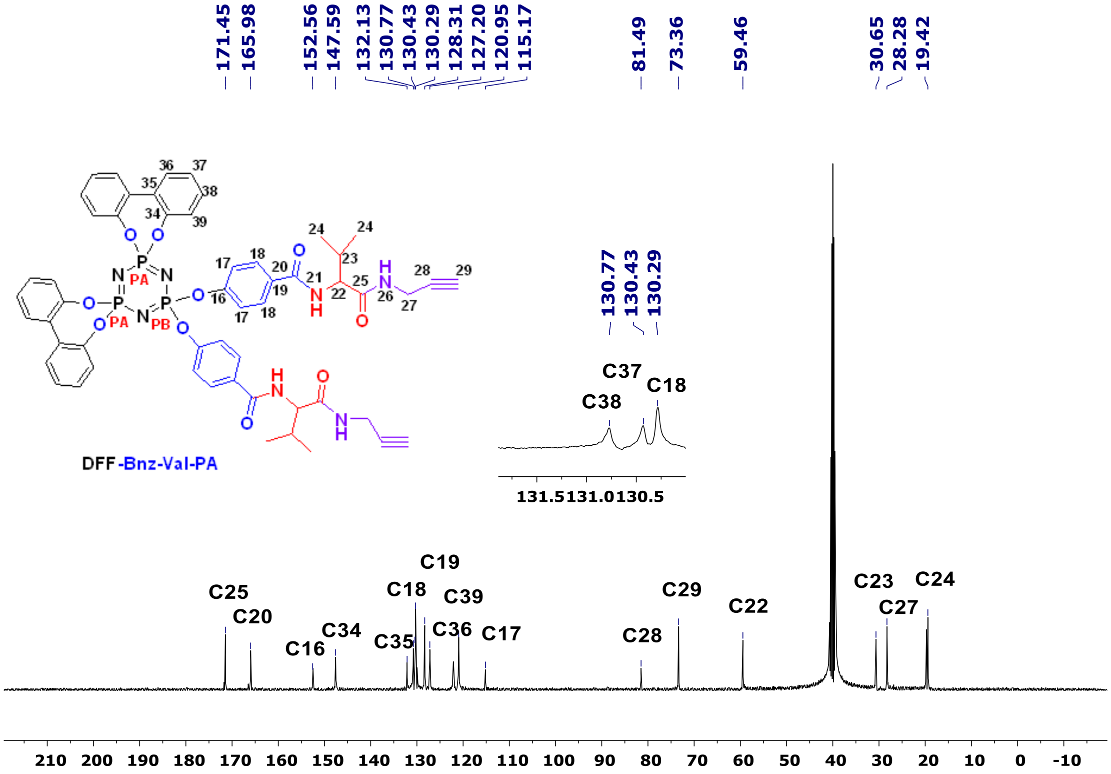

Figure S37. ^{13}C-NMR spectra of **DPP-Bnz-Val-PA (BV)** (DMSO-d_6)

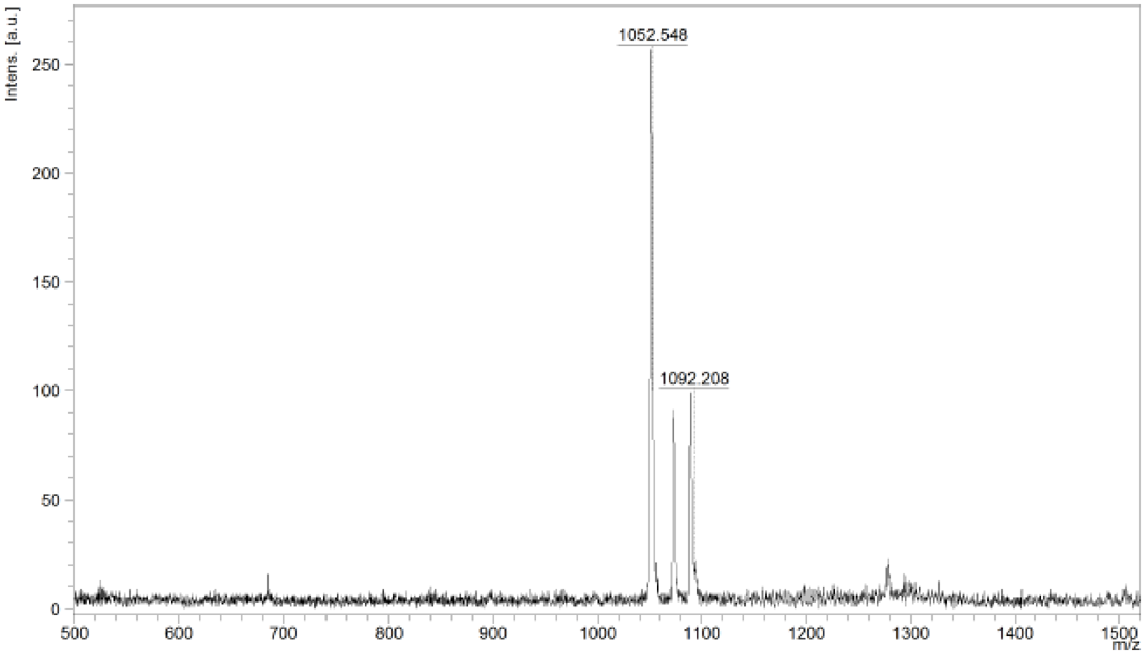

Figure S38. MALDI-TOF MS spectra of **DPP-Bnz-Val-PA (BV)**

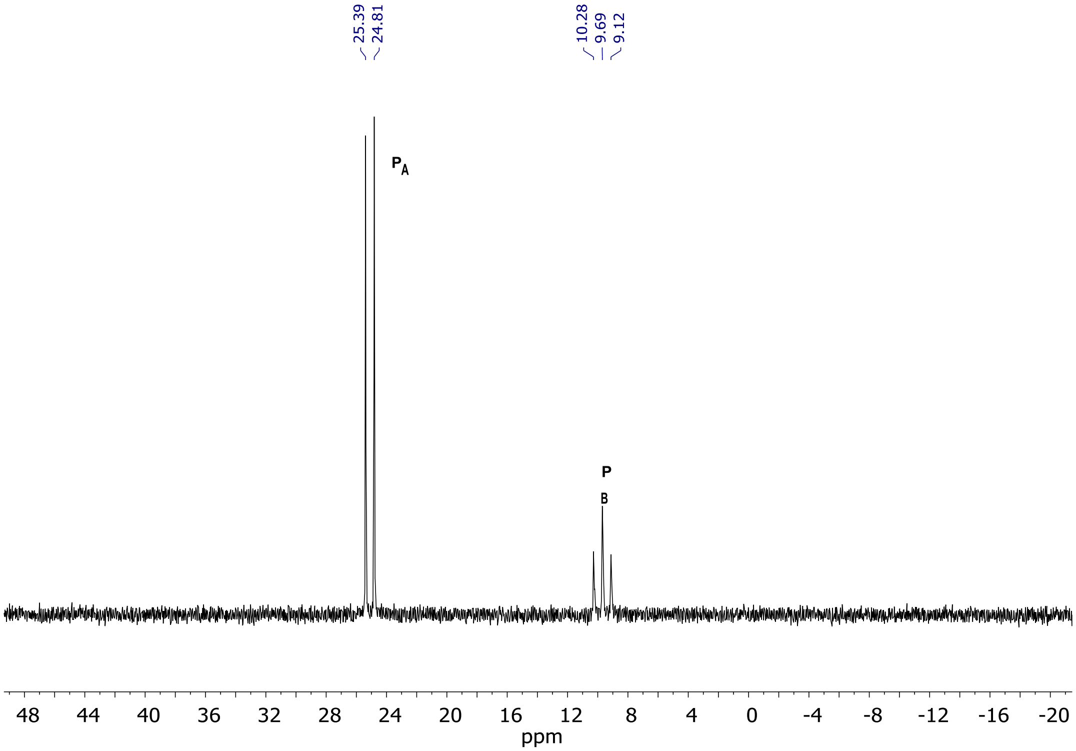

Figure S39. ^{31}P-NMR spectra of ***DPP-Bnz-Val-click-4-Cl-3-CF$_3$-chalcone (BVK1)***

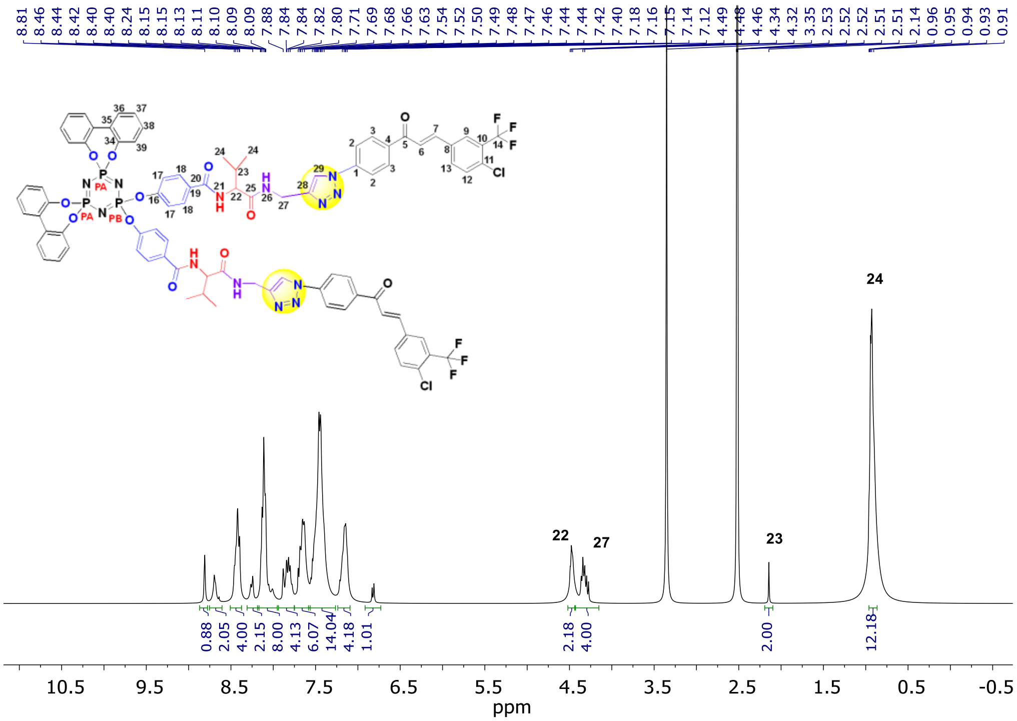

Figure S40. ^{1}H-NMR spectra of ***DPP-Bnz-Val-click-4-Cl-3-CF$_3$-chalcone (BVK1)***

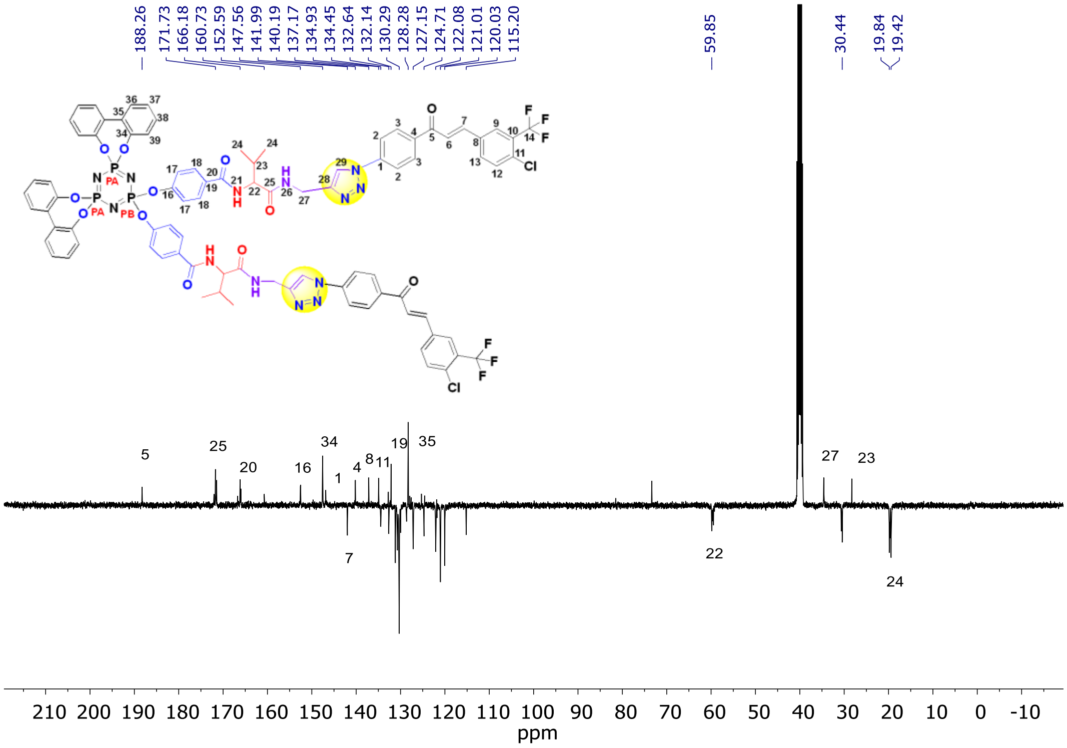

Figure S41. ^{13}C-NMR spectra of ***DPP-Bnz-Val-click-4-Cl-3-CF$_3$-chalcone (BVK1)***

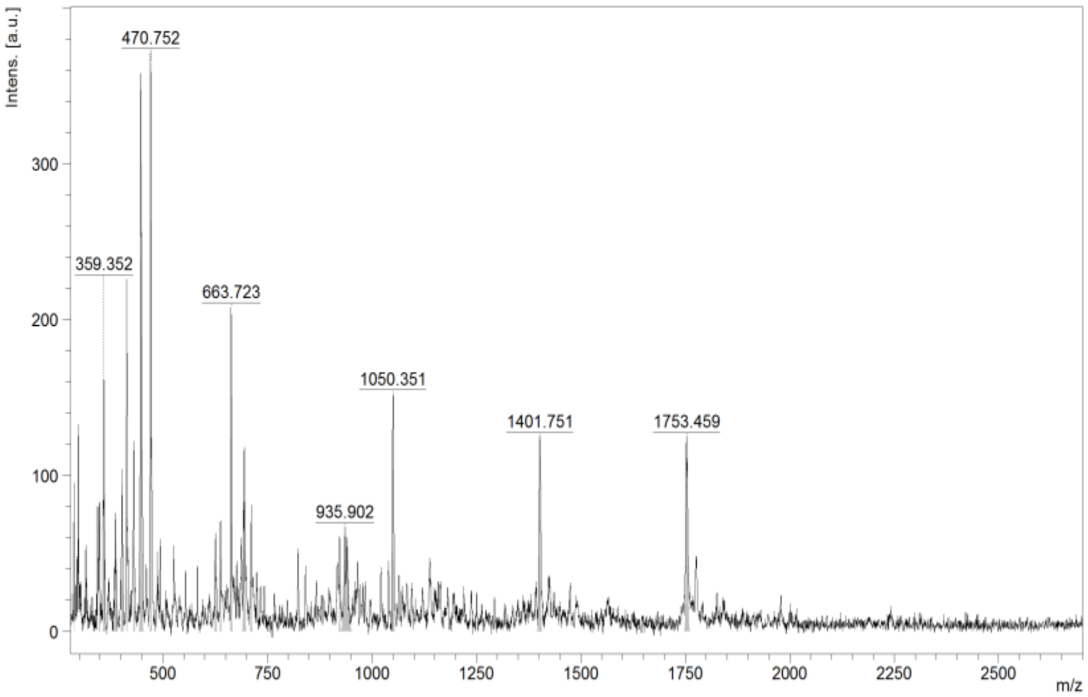

Figure S42. **MALDI-TOF MS spectra of** ***DPP-Bnz-Val-click-4-Cl-3-CF$_3$-chalcone*** ***(****BVK1****)***

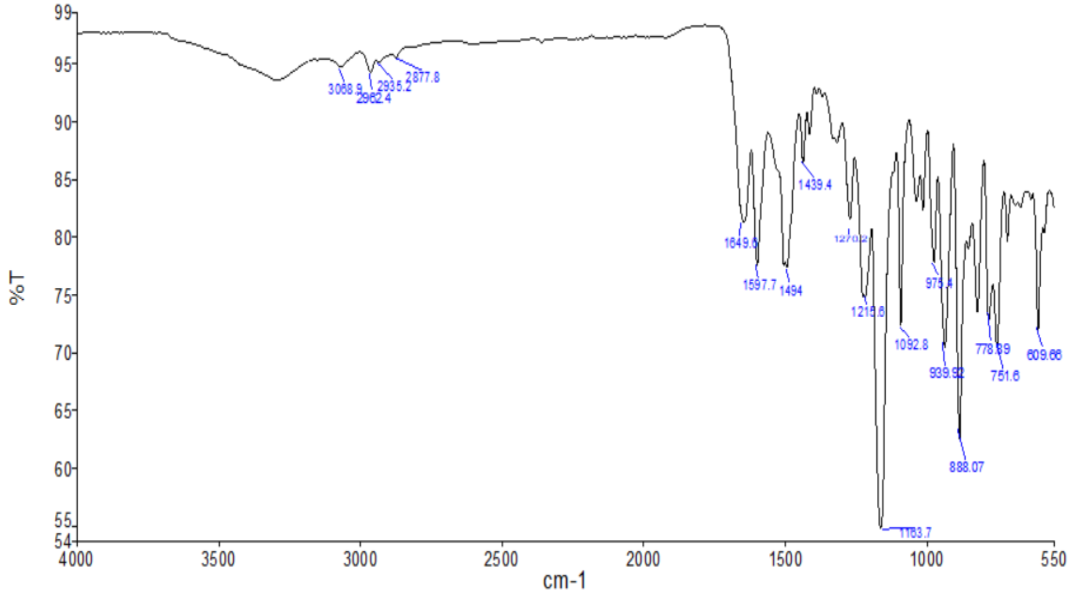

Figure S43. FT-IR (ATR) spectra of ***DPP-Bnz-Val-click-4-F-Chalcone (BVK2)***

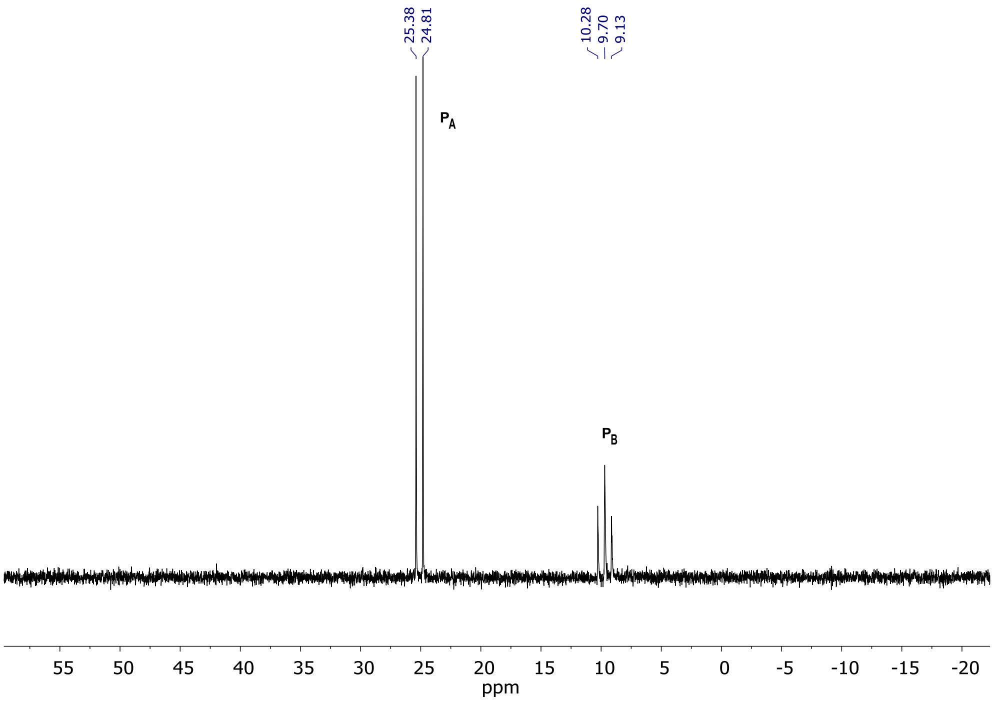

Figure S44. ^{31}P-NMR spectra of ***DPP-Bnz-Val-click-4-F-Chalcone (BVK2)***

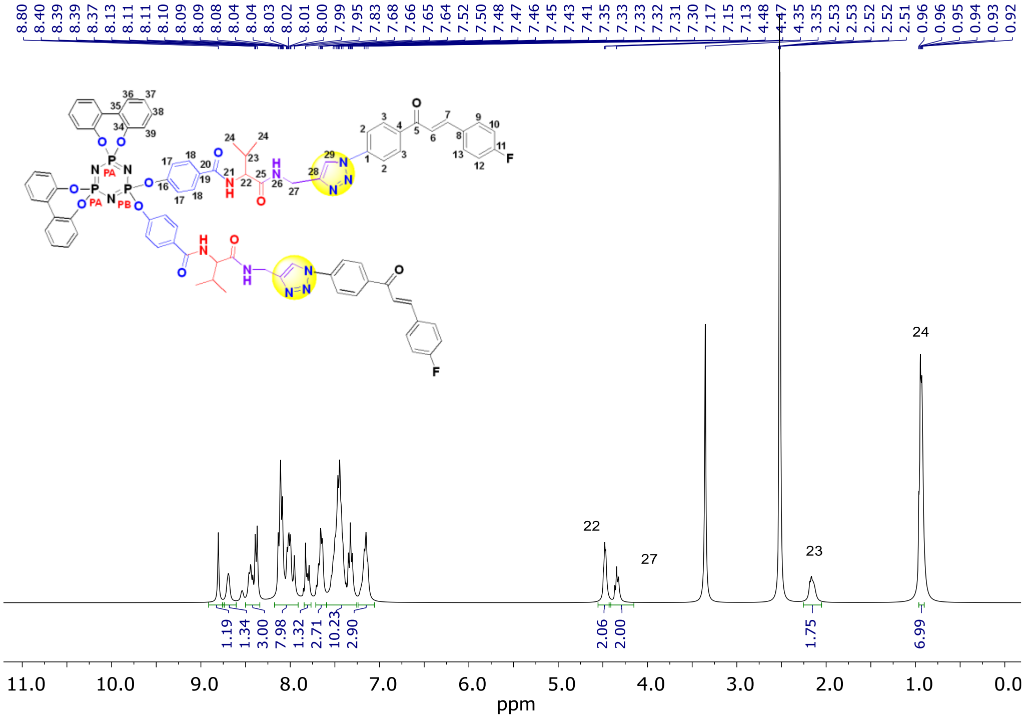

Figure S45. ^{1}H-NMR spectra of ***DPP-Bnz-Val-click-4-F-Chalcone (BVK2)***

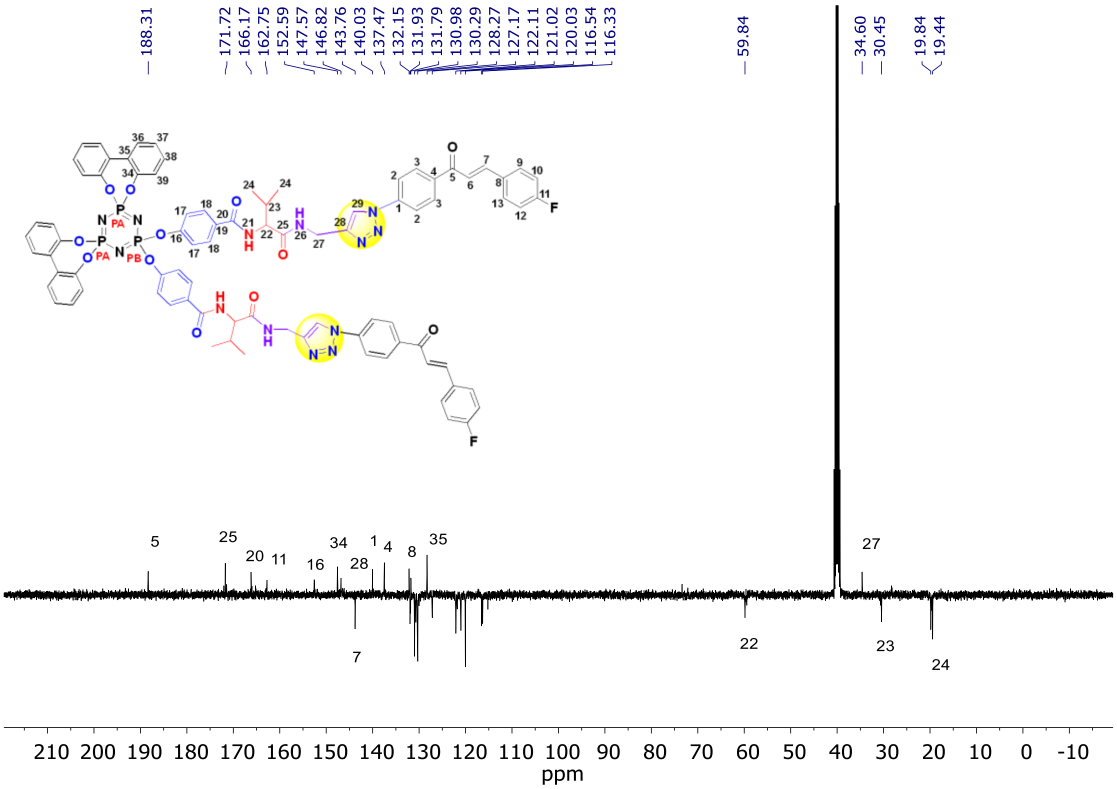

Figure S46. ^{13}C-NMR spectra of ***DPP-Bnz-Val-click-4-F-Chalcone (BVK2)***

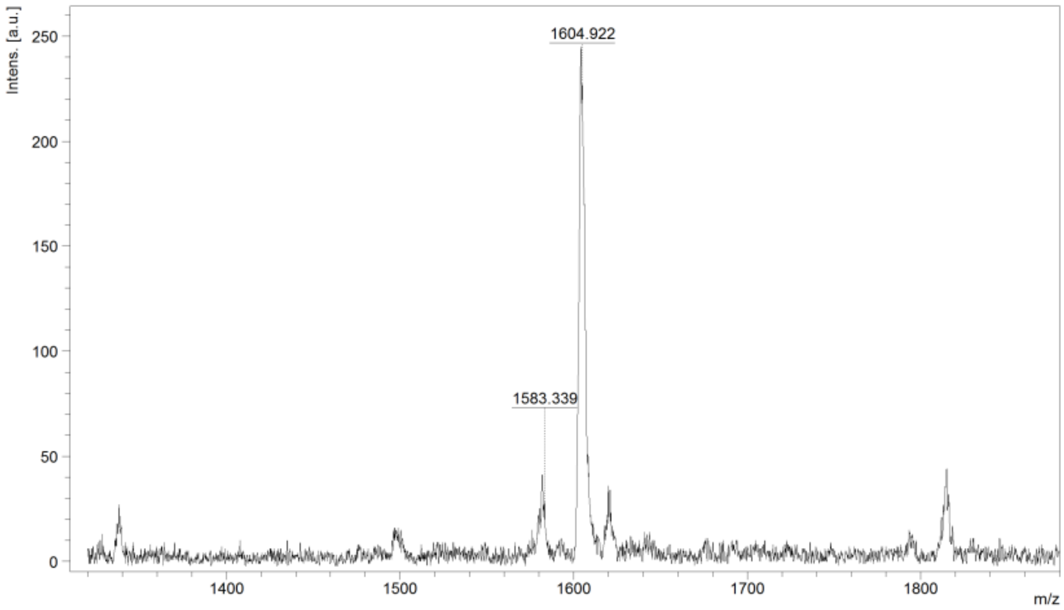

Figure S47. MALDI-TOF MS spectra of ***DPP-Bnz-Val-click-4-F-Chalcone (BVK2)***

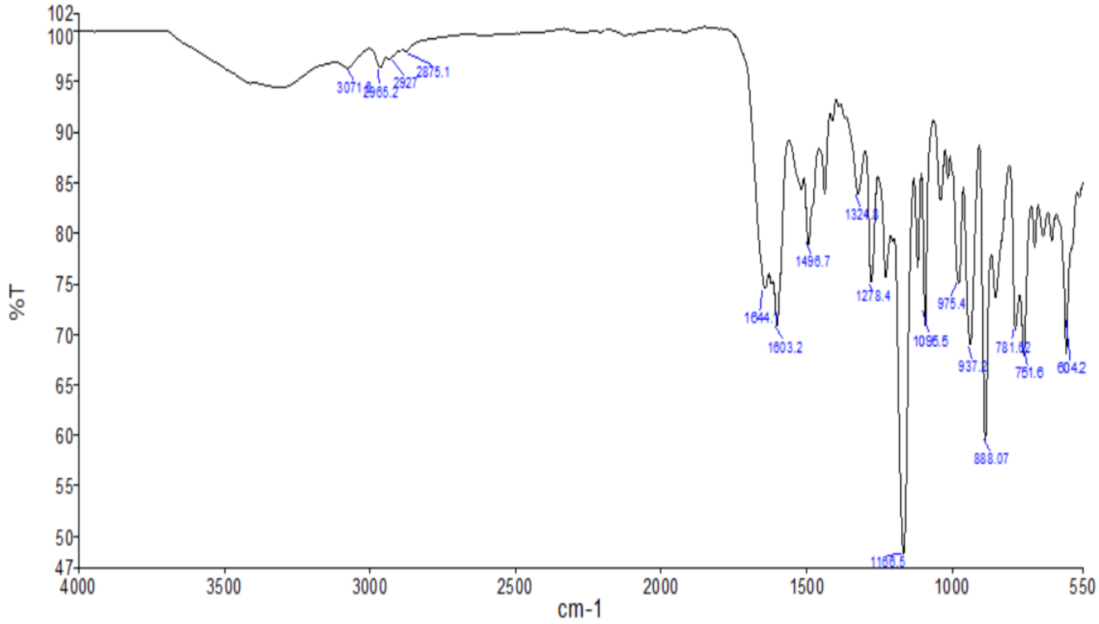

Figure S48. FT-IR (ATR) spectra of ***DPP-Bnz-Val-click-3,5-difluoro-chalcone (BVK3)*** (ATR)

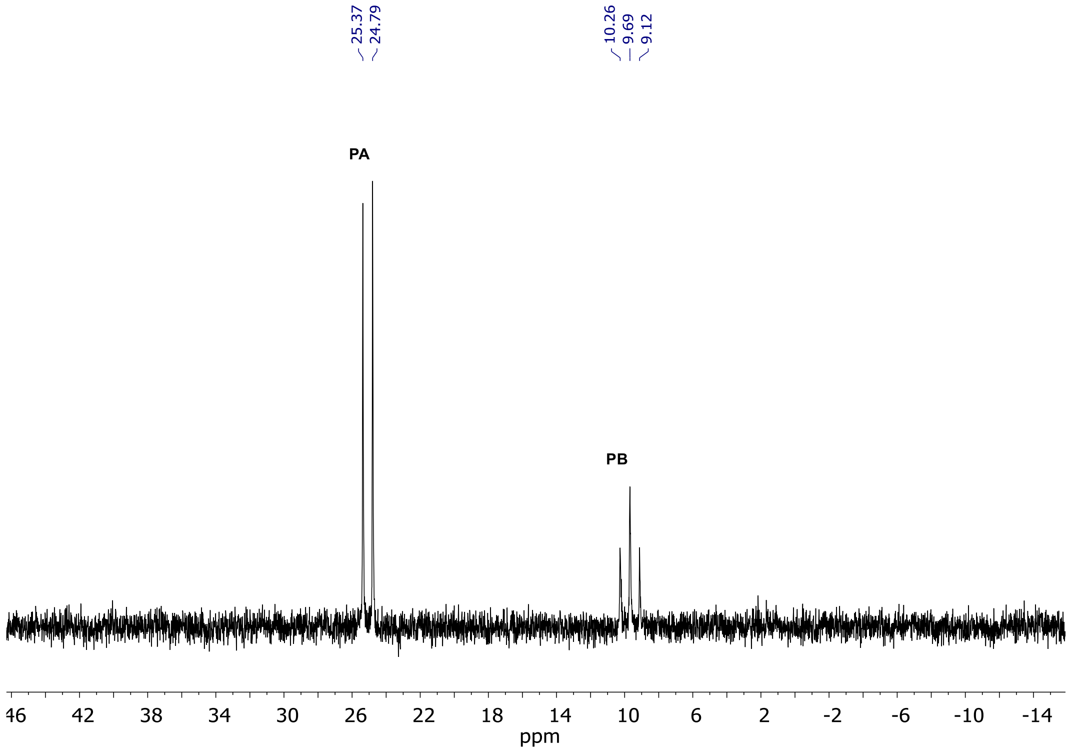

Figure S49. ^{31}P-NMR spectra of ***DPP-Bnz-Val-click-3,5-difluoro-chalcone (BVK3)***

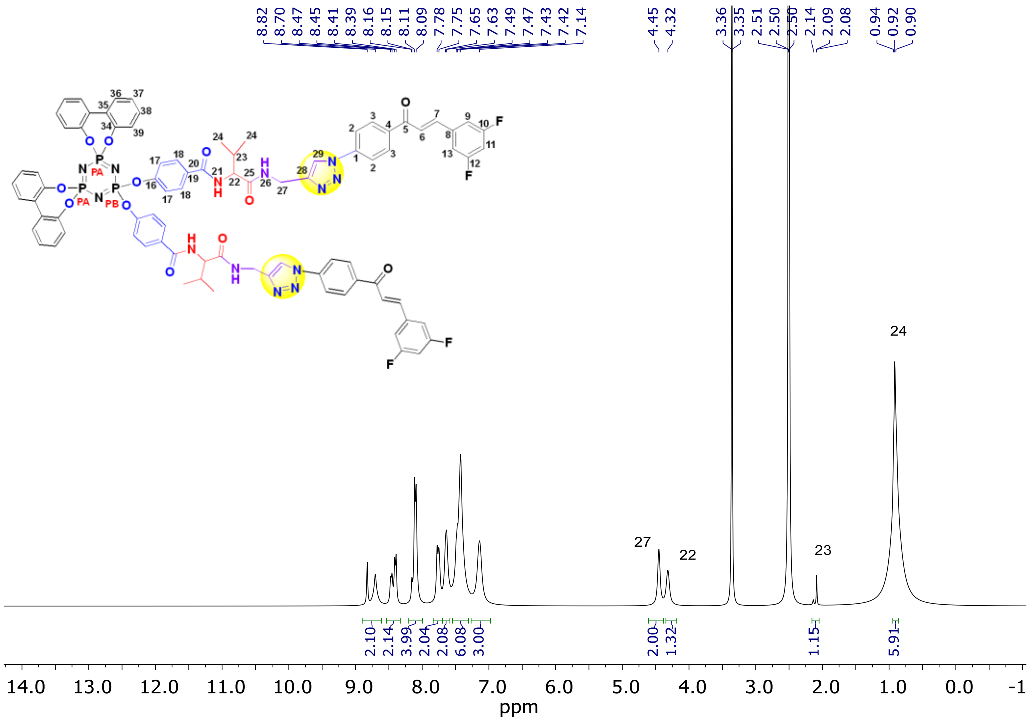

Figure S50. ^{1}H-NMR spectra of ***DPP-Bnz-Val-click-3,5-difluoro-chalcone (BVK3)***

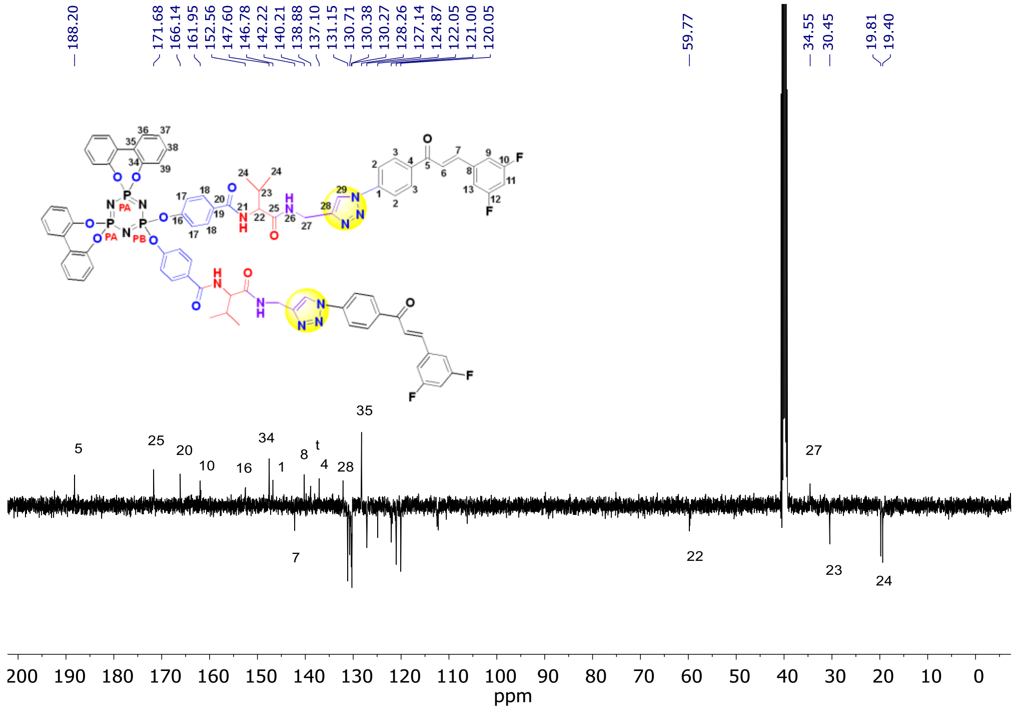

Figure S51. ^{13}C-NMR spectra of ***DPP-Bnz-Val-click-3,5-difluoro-chalcone (BVK3)***

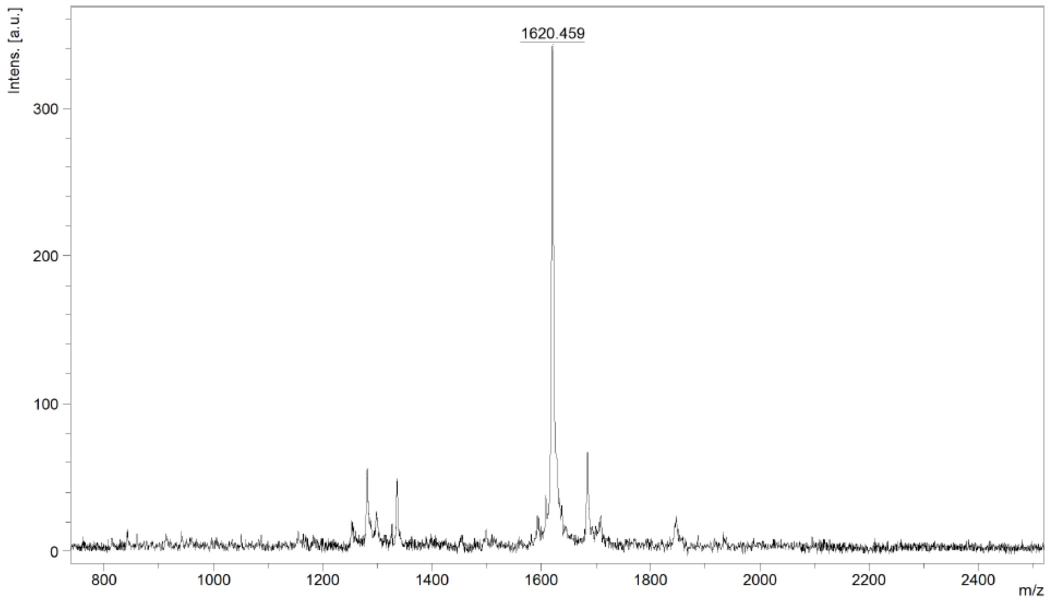

Figure S52. MALDI-TOF MS spectra of ***DPP-Bnz-Val-click-3,5-difluoro-chalcone (BVK3)***

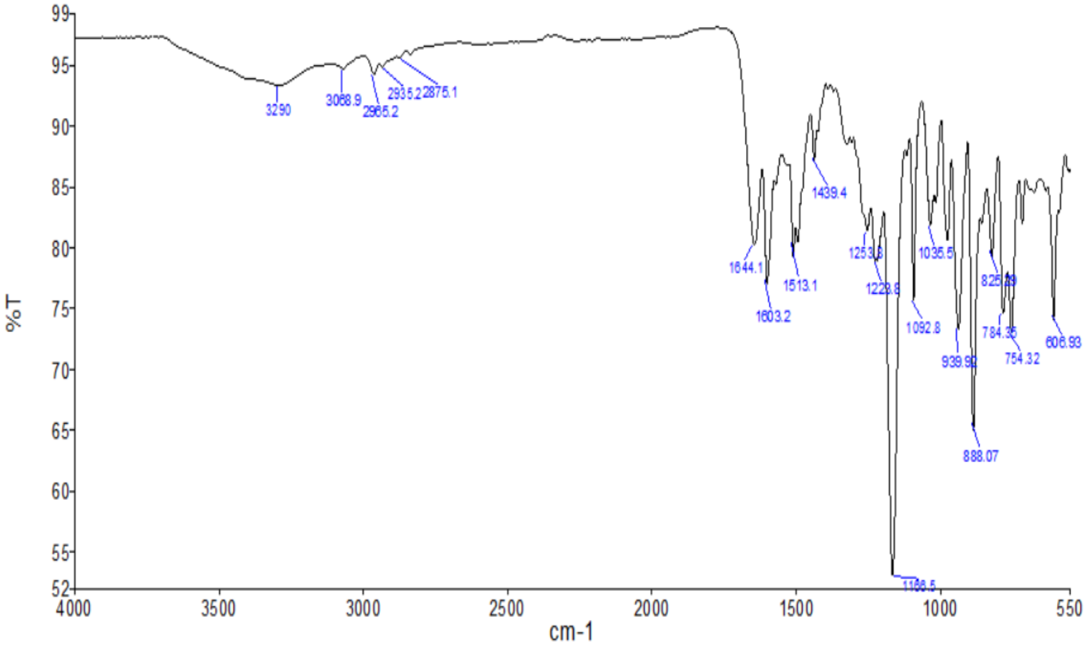

Figure S53. FT-IR (ATR) spectra of ***DPP-Bnz-Val-click-4-OCH$_3$-chalcone (BVK4)***

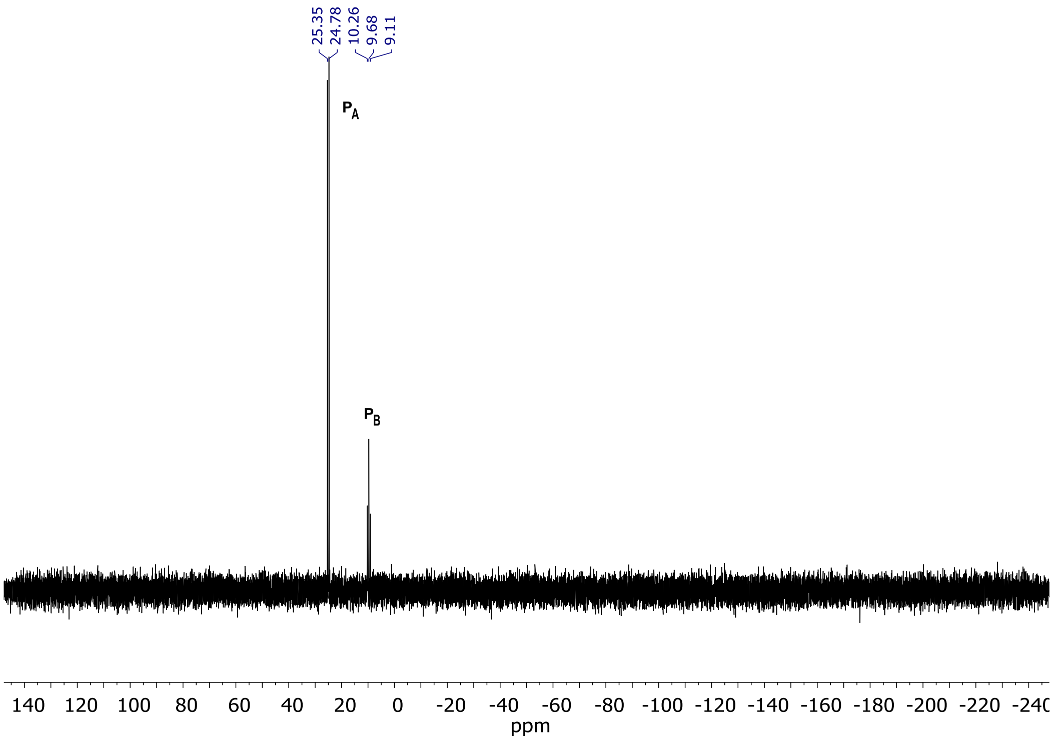

Figure S54. ^{31}P-NMR spectra of ***DPP-Bnz-Val-click-4-OCH$_3$-chalcone (BVK4)***

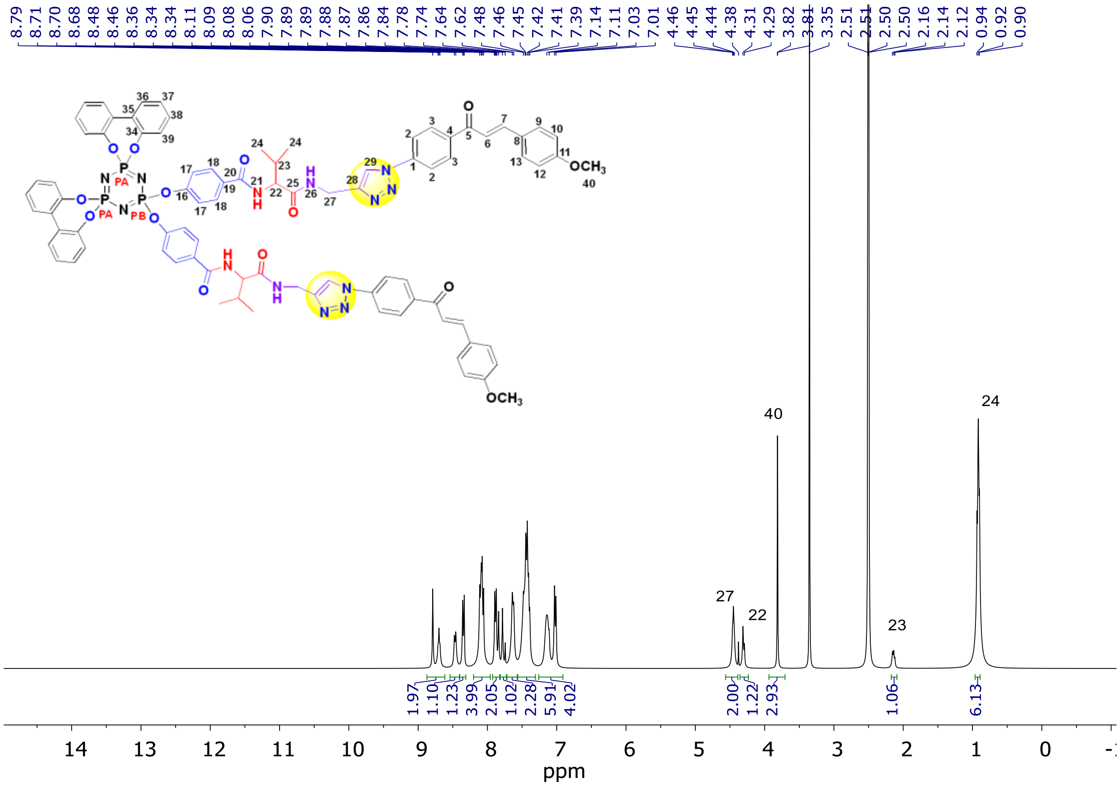

Figure S55. ^{1}H-NMR spectra of ***DPP-Bnz-Val-click-4-OCH$_3$-chalcone (BVK4)***

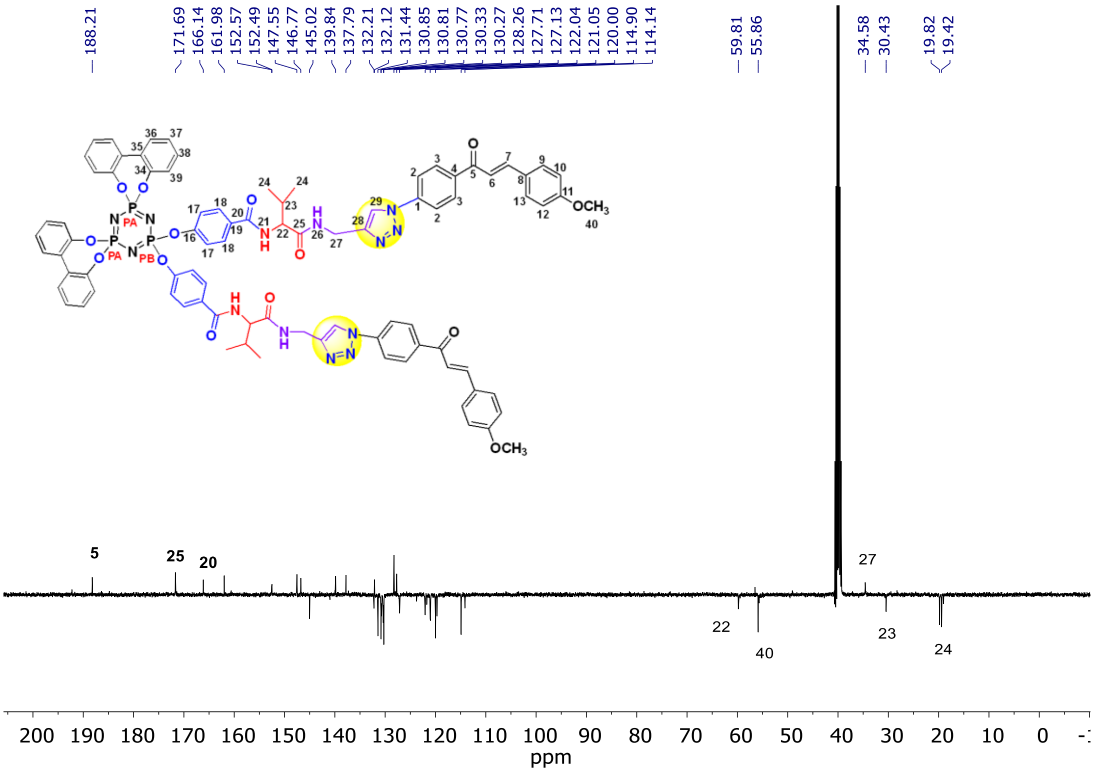

Figure S56. ^{13}C-NMR spectra of ***DPP-Bnz-Val-click-4-OCH$_3$-chalcone (BVK4)***

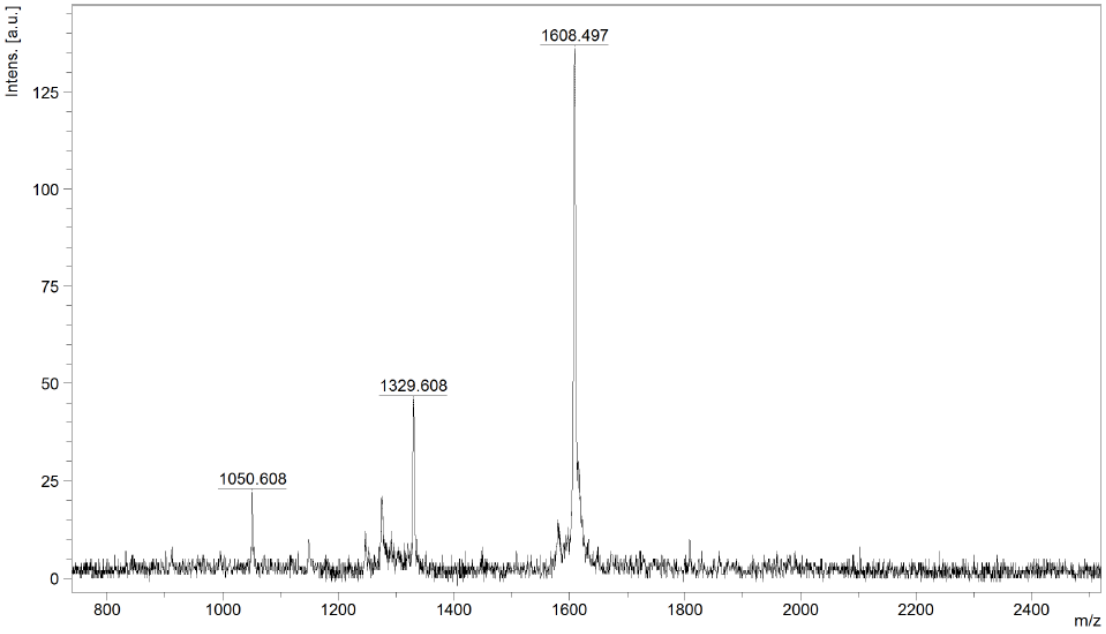

Figure S57. MALDI-TOF MS spectra of ***DPP-Bnz-Val-click-4-OCH$_3$-chalcone (BVK4)***

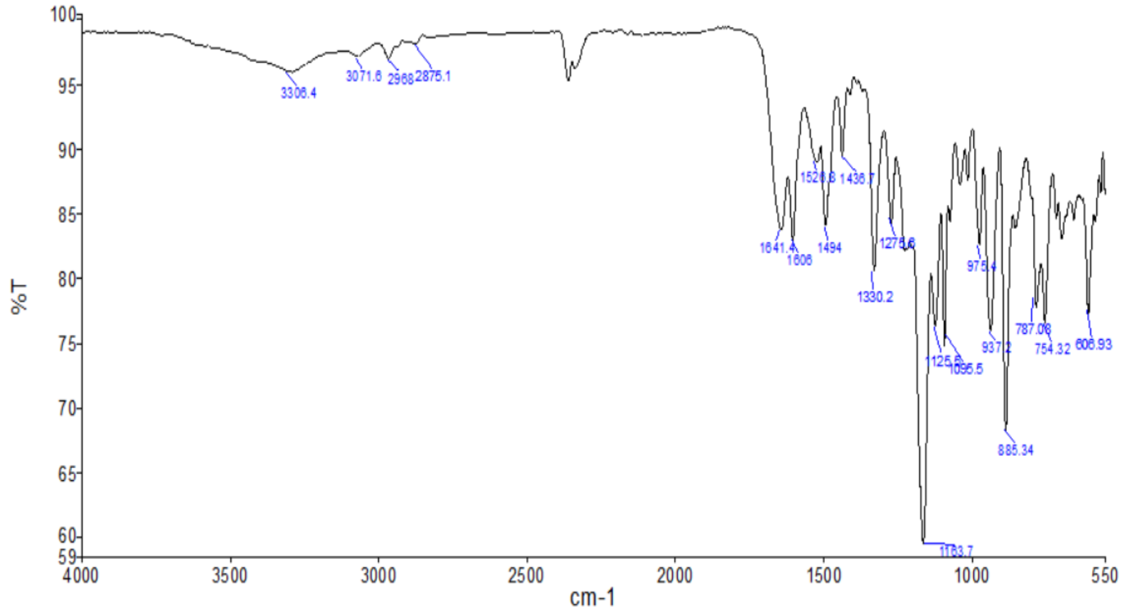

Figure S58. FT-IR (ATR) spectra of ***DPP-Bnz-Val-click-3-CF_3-chalcone (BVK5)***

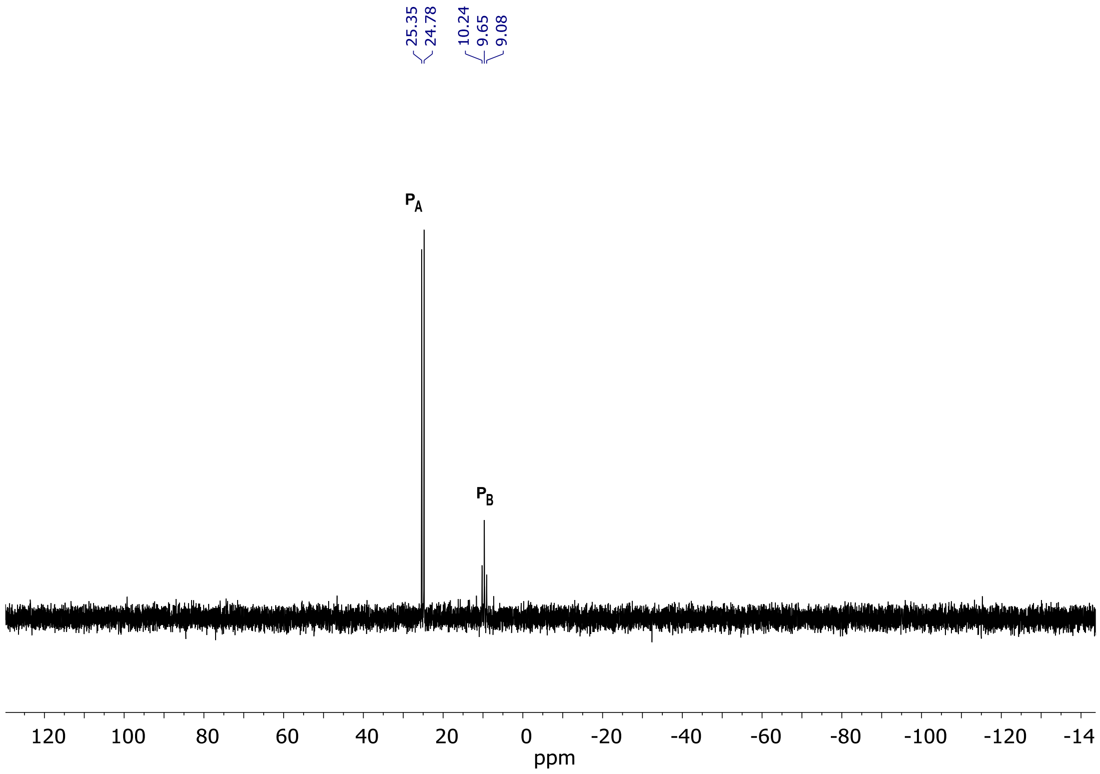

Figure S59. ^{31}P-NMR spectra of ***DPP-Bnz-Val-click-3-CF_3-chalcone (BVK5)***

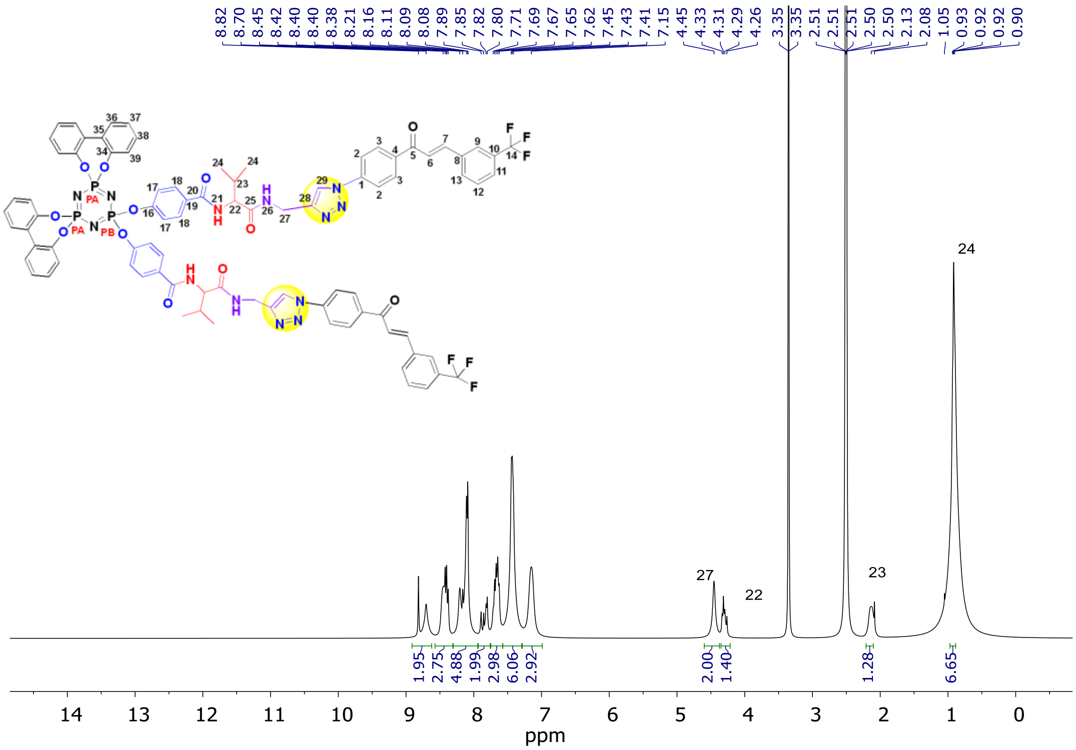

Figure S60. ^{1}H-NMR spectra of ***DPP-Bnz-Val-click-3-CF$_3$-chalcone (BVK5)***

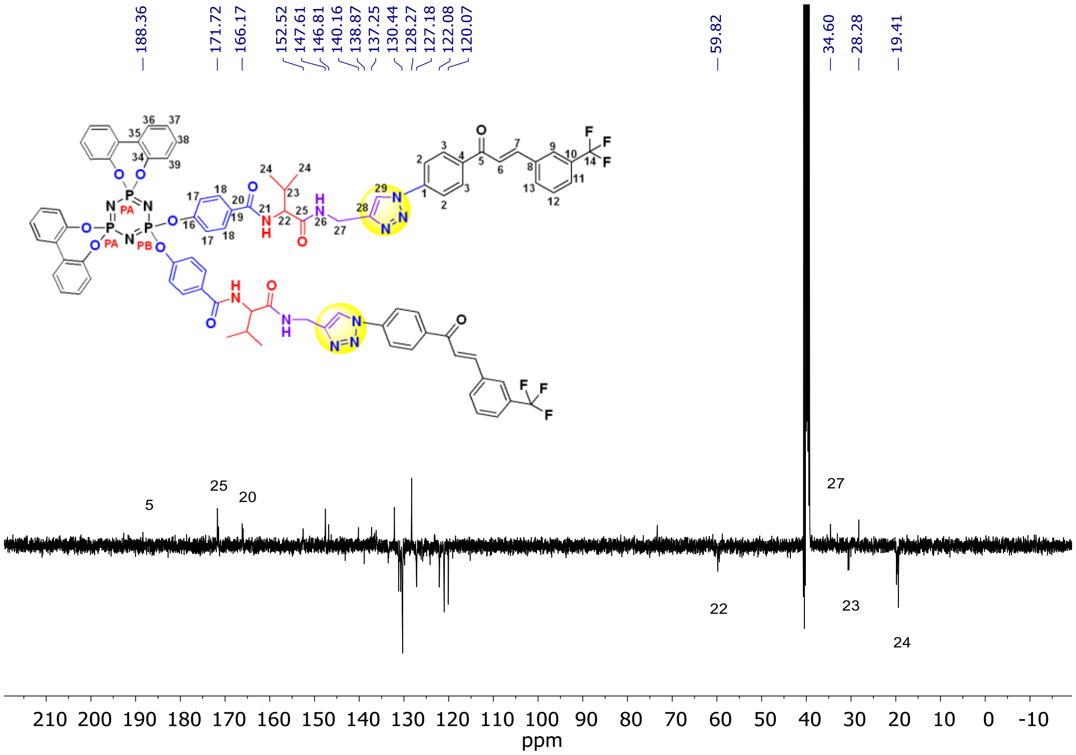

Figure S61. ^{13}C-NMR spectra of ***DPP-Bnz-Val-click-3-CF$_3$-chalcone (BVK5)***

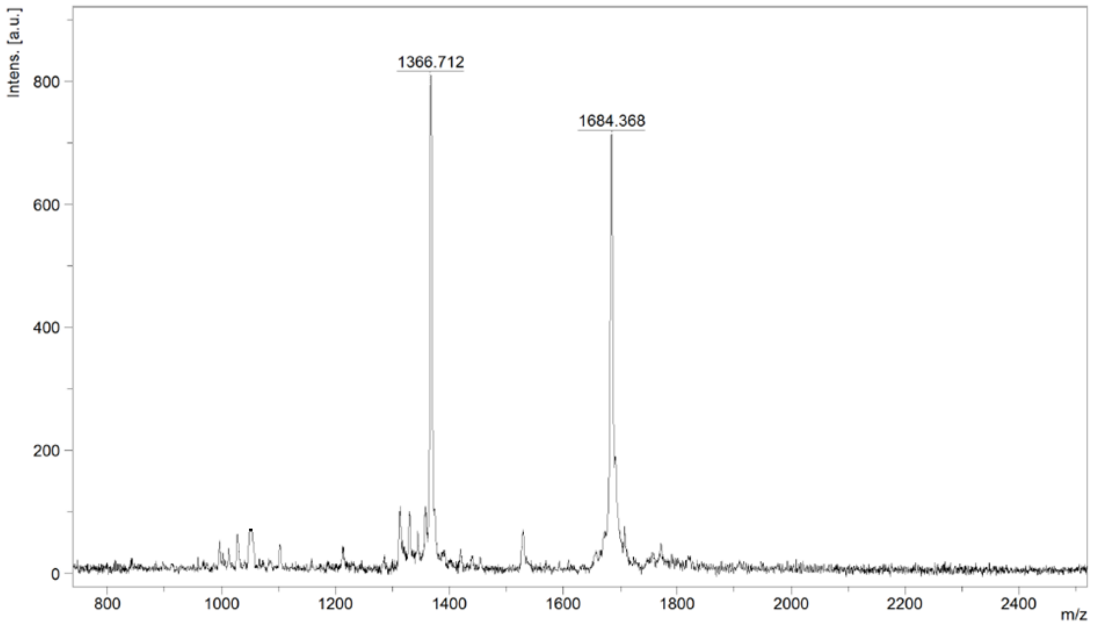

Figure S62. MALDI-TOF MS spectra of ***DPP-Bnz-Val-click-3-CF_3-chalcone (BVK5)***

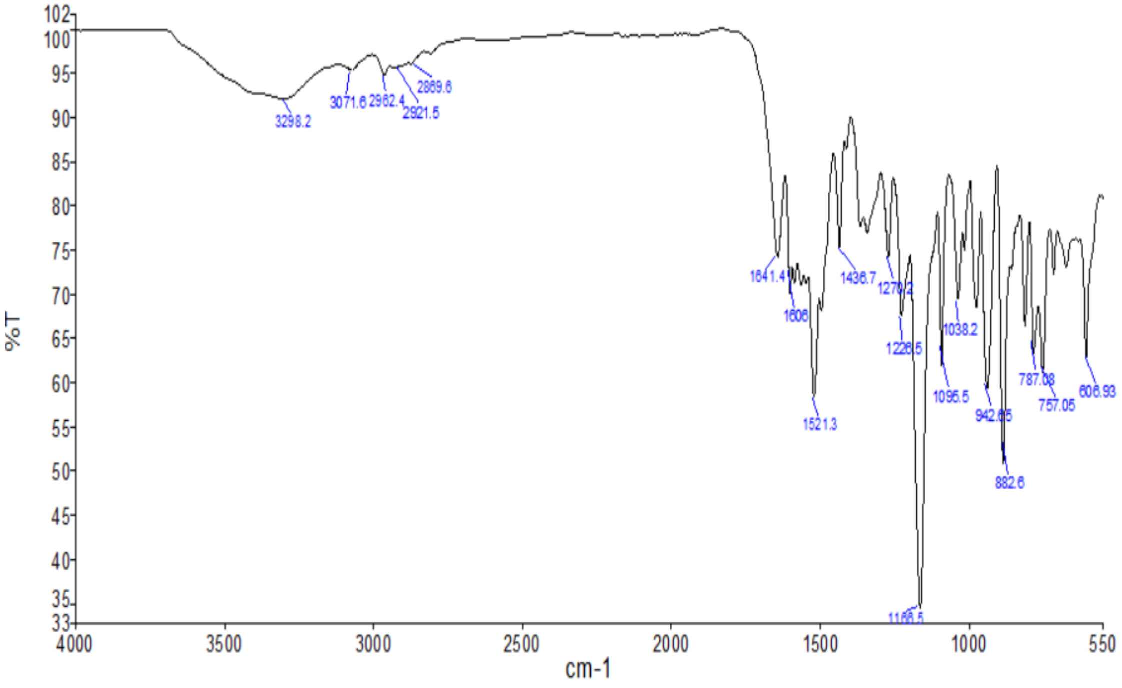

Figure S63. FT-IR (ATR) spectra of ***DPP-Bnz-Val-click-4-$N(CH_3)_2$-chalcone (BVK6)***

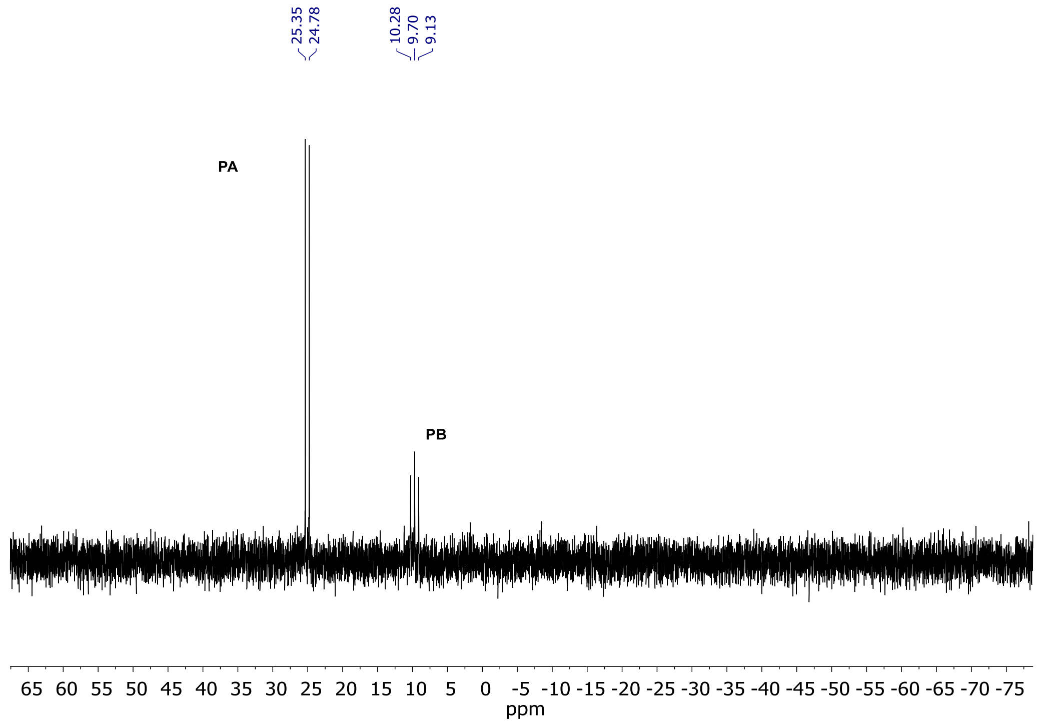

Figure S64. ^{31}P-NMR spectra of ***DPP-Bnz-Val-click-4-N(CH$_3$)$_2$-chalcone (BVK6)***

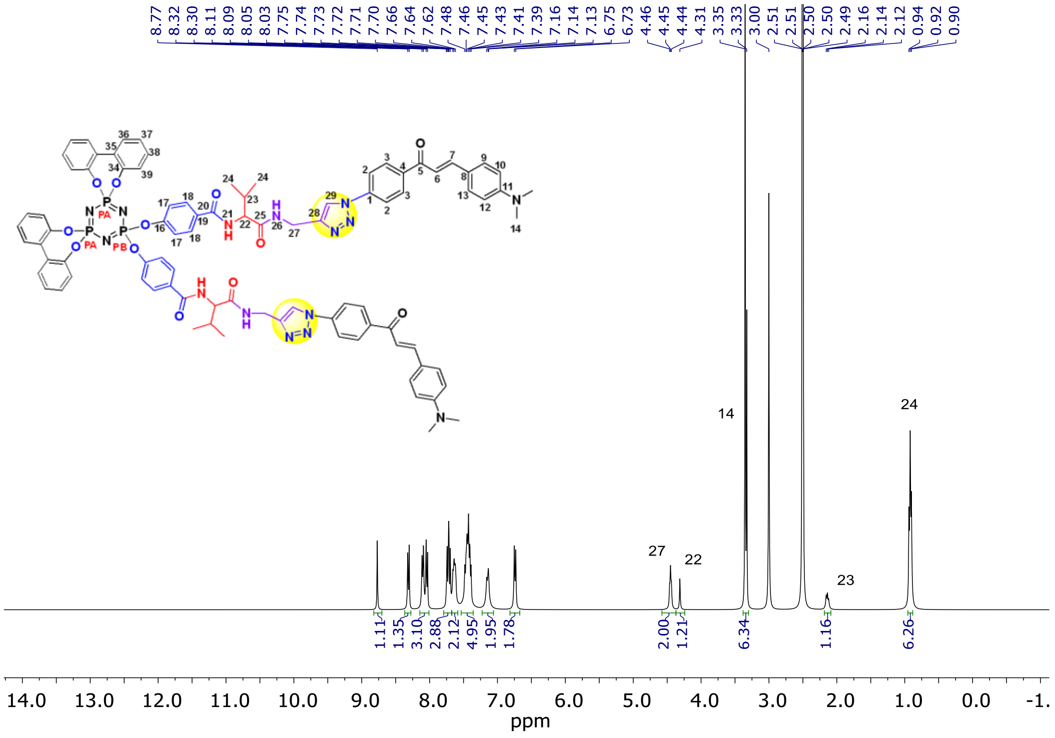

Figure S65. ^{1}H-NMR spectra of ***DPP-Bnz-Val-click-4-N(CH$_3$)$_2$-chalcone (BVK6)***

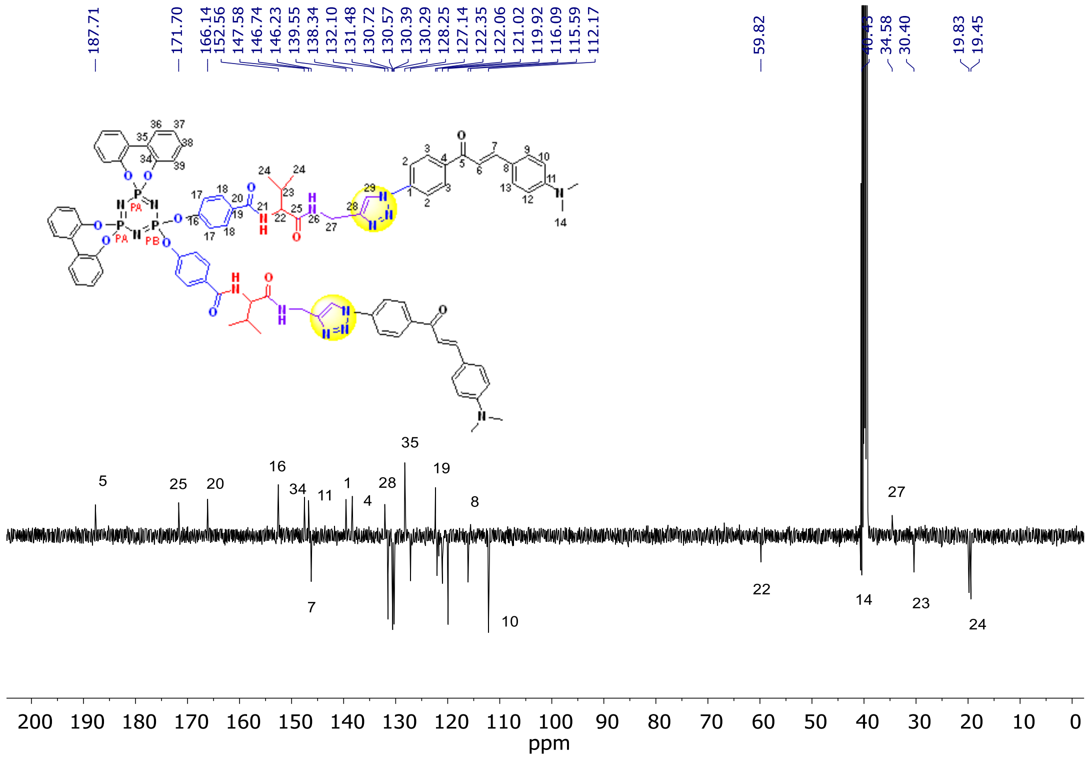

Figure S66. ^{13}C-NMR spectra of ***DPP-Bnz-Val-click-4-N(CH$_3$)$_2$-chalcone (BVK6)***

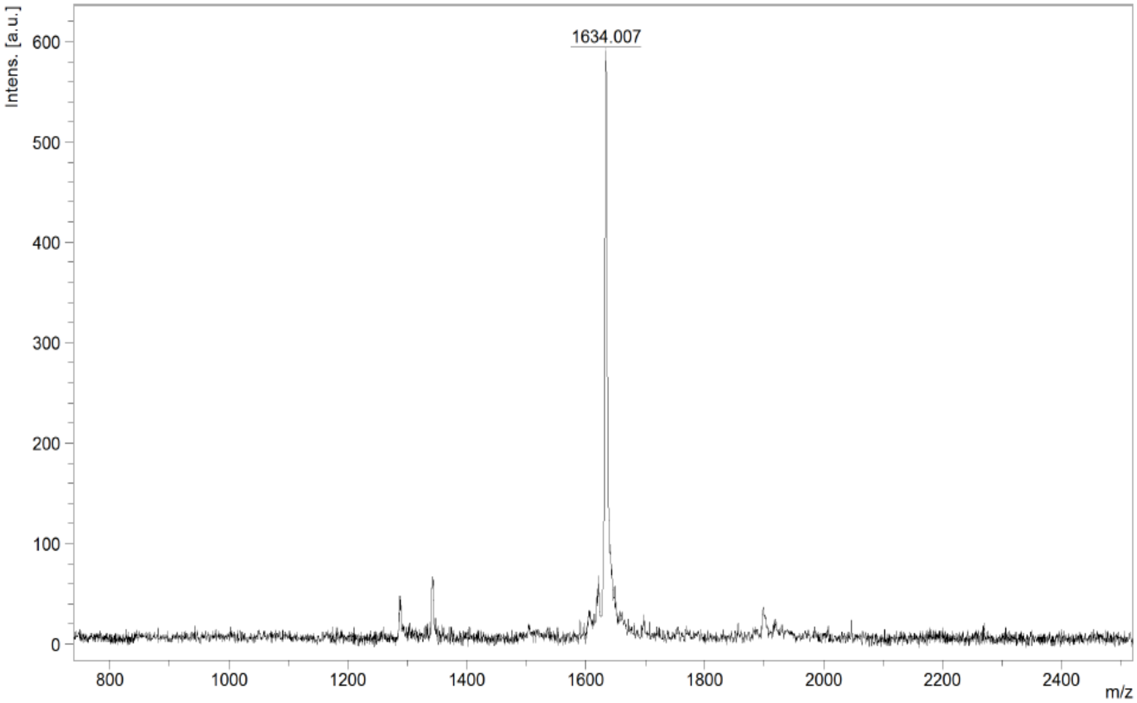

Figure S67. MALDI-TOF MS spectra of ***DPP-Bnz-Val-click-4-N(CH$_3$)$_2$-chalcone (BVK6)***
